# Derivatization agents for LC-MS analysis of amino acids: effects of core structure and functional groups

**DOI:** 10.1007/s00216-026-06366-9

**Published:** 2026-02-10

**Authors:** Tereza Hofmanova, Rudolf Andrys, Miroslav Lisa

**Affiliations:** Department of Chemistry, Faculty of Science, University of Hradec Kralove, Rokitanskeho 62, 50003 Hradec Kralove, Czech Republic

**Keywords:** Amino acids, LC-MS, Derivatization, NHS ester, Isoquinoline

## Abstract

The analysis of amino acids in biological samples is challenged by their high polarity, low ionization efficiency, and lack of chromophores, which limit detection by LC-UV or LC-MS. Derivatization is widely used to improve retention, sensitivity, and detection. In this study, derivatization agents based on pyridine, quinoline, and isoquinoline positional isomers scaffolds with COCl, SO₂Cl, and NHS ester reactive groups were systematically evaluated using deuterium-labeled amino acids to assess stability, derivatization efficiency, chromatographic behavior, and MS response. NHS ester-based agents were found to exhibit superior stability, maintaining activity for over 1 year, whereas carbonyl chlorides and sulfonyl chloride–based agents were highly reactive but less stable. NHS ester of isoquinoline-6-carboxylic acid (6-CiQ-NHS) was identified as the most effective agent, combining rapid derivatization, strong MS signal, and stability. LC-MS analysis demonstrated excellent linearity (*R*^2^ ≥ 0.995), low nanomolar detection limits (0.23–6.33 nM), and separation of isomeric amino acids (e.g., isoleucine/leucine). 6-CiQ-NHS is proposed as a practical derivatization approach for amino acid quantification and provides a framework for the rational design of future agents.

**Graphical Abstract:**

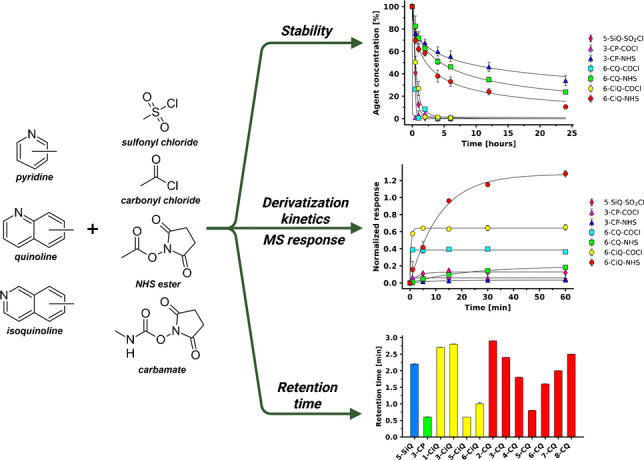

**Supplementary Information:**

The online version contains supplementary material available at 10.1007/s00216-026-06366-9.

## Introduction

Amino acids are fundamental biomolecules involved in protein biosynthesis, metabolism, and cell signalling [1]. For instance, glutamate contributes to hepatic pH regulation [2] and γ-aminobutyric acid (GABA) or d-serine acts as an important neurotransmitter in the central nervous system [3, 4]. Alterations in amino acid levels are closely linked to physiological and pathological states, such as phenylketonuria or neurodegenerative diseases. This correlation makes them potentially valuable clinical biomarkers [5, 6]. Beyond diagnostic, amino acids are also explored for therapeutic applications. As an example, l-serine has been studied as a protective agent against cyanobacterial neurotoxins [7].

Liquid chromatography (LC) is the primary technique for amino acid analysis [8]. However, the direct determination of intact amino acids presents significant analytical challenges. The majority of them lack chromophores or fluorophores, limiting their detection by conventional UV and fluorescence (FLD) detectors. LC coupled with tandem mass spectrometry (LC-MS) is thus often required to achieve sufficient sensitivity [9]. The high polarity of intact amino acids, however, results in insufficient retention on reversed-phase (RP) columns [10]. Similarly, alternative separation by hydrophilic interaction liquid chromatography (HILIC) often lacks comparable reliability. The absence of structural features also compromises the chromatographic resolution of critical isomeric pairs, particularly leucine and isoleucine [11]. Moreover, intact amino acids typically exhibit poor electrospray ionization (ESI) efficiency, which can reduce MS response, especially in complex biological samples [12].

To overcome these limitations, chemical derivatization is widely employed to modify the amino acid structure with moieties that enhance retention, ionization efficiency, or optical detection [12]. A variety of derivatization agents, including dansyl chloride (DNS) [13, 14], o-phthalaldehyde (OPA) [15], 6-aminoquinolyl-N-hydroxysuccinimidyl carbamate (AQC) [16, 17], 9-fluorenylmethoxycarbonyl chloride (Fmoc-Cl) [18, 19], and 4-(phenylazo)benzoic acid N-succinimidyl ester (AzoC) [9], have been developed. These agents typically utilize diverse reactive groups, most commonly carbonyl chlorides (e.g., Fmoc-Cl), carbamates (e.g., AQC), sulfonyl chlorides (e.g., DNS), or *N*-hydroxysuccinimide (NHS) esters (e.g., AzoC).

Despite their widespread use, existing agents possess notable drawbacks, starting with limited storage stability. Certain agents, such as AQC, degrade rapidly once opened. Further challenges arise during use in solution where, for example, sulfonyl and carbonyl chlorides are prone to rapid hydrolysis. This degradation often produces poorly soluble byproducts—such as sulfonic acids or alcohols (e.g., 9-fluorenylmethanol (Fmoc-OH) from Fmoc-Cl)—that can precipitate and accumulate within the LC-MS system, impairing analysis performance [20, 21]. Furthermore, MS/MS fragmentation of certain derivatives (e.g., those from Fmoc-Cl) often leads to the loss of the ionizable moiety, preventing the achievement of required detection limits [18].

This study focused on the systematic evaluation of a derivatization agent based on a series of nitrogen-containing heterocyclic cores (pyridine, quinoline, and isoquinoline) modified with various reactive groups (carbonyl chlorides, sulfonyl chlorides, NHS esters, and carbamates) at different positions. This approach allowed for a comprehensive assessment of how the aromatic structure and reactive groups impact key physicochemical and analytical properties. The study evaluated the synthetic feasibility, stability, spectroscopic properties (UV, FLD), and MS performance of the prepared agents. The most suitable candidate was subsequently applied to the development and validation of an LC-MS method for the analysis of the 20 proteinogenic amino acids.

## Materials and methods

### Materials

Pyridine-, quinoline-, and isoquinoline-carboxylic/sulfonic acids were purchased from Activate Scientific (Germany). N,N′-Disuccinimidyl carbonate (DSC), pyridine-3-amine, isoquinoline-5-amine, thionyl chloride, dimethylformamide (DMF), formic acid, acetic acid, ammonium formate, ammonium acetate, and Deuterated amino acid standard mixture (l-His-[d_5_] (50 μM), l-Asp-[d_3_] (48 μM), l-Ser-[d_3_] (60 μM), l-Gln-[d_5_] (222 μM), l-Arg-[d_7_] (48 μM), l-Gly-[d_2_] (152 μM), l-Asp-[d_3_] (20 μM), l-Glu-[d_5_] (64 μM), l-Thr-[d_5_] (81 μM), l-Ala-[d_4_] (164 μM), l-Pro-[d_7_] (84 μM), l-Lys-[d_8_] (70 μM), l-Tyr-[d_7_] (45 μM), l-Met-[d_8_] (24 μM), l-Val-[d_8_] (156 μM), l-Ile-[d_10_] (39 μM), l-Leu-[d_10_] (85 μM), l-Phe-[d_8_] (44 μM), l-Trp-[d_8_] (31 μM)) were purchased from Merck (USA). Triethylamine, dichloromethane, chloroform, and ethyl acetate were purchased from Penta Chemicals (Czech Republic). Amino acid standard mixture of 17 l-amino acids (1 nmol/μL in 0.1 M HCl) was obtained from Agilent Technologies (USA). Additional l-amino acid standards (asparagine, glutamine, tryptophan, and norvaline) were purchased from Merck (USA). Certified reference material NIST SRM 1950 plasma was obtained from the National Institute of Standards and Technology (USA). LC-MS grade methanol (MeOH) and acetonitrile (ACN) were purchased from Honeywell (USA). Ultrapure water was prepared using a Merck Millipore Simplicity UV system.

### Synthesis of derivatization agents

#### General procedure for the preparation of sulfonyl chloride derivatization agents

The *pyridine-3-sulfonic acid* (**3-SP**) or *isoquinoline-5-sulfonic acid* (**5-SiQ**) (150 mg) was dissolved in thionyl chloride (0.63 mL), followed by the catalytic addition of DMF (50 μL). The reaction mixture was heated at 75 °C under a nitrogen atmosphere for 1.5 h. After completion, the excess thionyl chloride was evaporated. The crude product was partitioned between dichloromethane (3 × 5 mL) and saturated aqueous NaHCO₃ solution (5 mL). The combined organic phases were dried over Na₂SO₄, and the solvent was evaporated. Further details on the products’ NMR spectra are provided in the Supporting Information (Chapter 1.2, Figures S1–S2).

#### General procedure for the preparation of carbonyl chloride derivatization agents

The *pyridine-3-carboxylic acid* (**3-CP**), *isoquinoline-5-carboxylic acid* (**5-CiQ**), *isoquinoline-6-carboxylic acid* (**6-CiQ**), or *quinoline-6-carboxylic acid* (**6-CQ**) (30 mg) was dissolved in thionyl chloride (0.5 mL). The reaction mixture was heated at 70 °C with a nitrogen atmosphere overnight. The excess thionyl chloride was evaporated. Further details on the NMR spectra and the product mass are provided in the Supporting Information (Chapter 1.2, Figures S3–S6).

#### General procedure for the preparation of NHS derivatization agents

The **3-CP**, *isoquinoline-1-carboxylic acid* (**1-CiQ**), *isoquinoline-3-carboxylic acid* (**3-CiQ**), **5-CiQ**, **6-CiQ**, *quinoline-2-carboxylic acid* (**2-CQ**), *quinoline-3-carboxylic acid* (**3-CQ**), *quinoline-4-carboxylic acid* (**4-CQ**), *quinoline-5-carboxylic acid* (**5-CQ**), **6-CQ**, *quinoline-7-carboxylic acid* (**7-CQ**), or *quinoline-8-carboxylic acid* (**8-CQ**) (50 mg) was dissolved in ACN (3 mL), together with DSC (74 mg, 1.0 equiv). Triethylamine (40 μL, 1.0 equiv) was added, and the reaction mixture was stirred at room temperature overnight. The solvent was evaporated, and the crude product was purified by isocratic column chromatography on silica gel using chloroform/ethyl acetate (3:1, v/v) as the mobile phase. Further details on the products’ NMR spectra are provided in the Supporting Information (Chapter 1.2, Figures S7–S18).

### Determination of derivatization agents’ stability in organic solvents

The derivatization agent was dissolved in (i) ACN or (ii) a 1:1 mixture of ACN and 5 mM sodium tetraborate buffer (pH 9.0) to a final concentration of 0.5 mg/mL. Solutions were incubated at 25 °C and analyzed by LC-UV (190–800 nm) at predetermined time points (0, 0.5, 1, 2, 4, 6, 12, and 24 h) using an injection volume of 0.5 μL. Chromatograms were evaluated at the absorption maxima of each agent to monitor the degradation of the parent compound. Due to the observed precipitation, compounds 5-CQ-NHS, 7-CQ-NHS, and DNS were further diluted 1:1 (v/v) with acetone and centrifuged (25,000 × *g*, 20 min) prior to analysis.

### Determination of fluorescent properties of derivatization agents

The fluorescence properties of agents were characterized using a Tecan Spark plate reader (Tecan, Switzerland) with black 96-well plates. Stock solutions of each derivatization agent (1 mg/mL in ACN) were diluted with 1% aqueous formic acid to a final concentration of 0.5 mg/mL. Excitation and emission spectra were recorded in the 280–600 nm range to determine the excitation and emission maxima for each agent. Subsequently, fluorescence intensities were quantified at these specific wavelengths. All measurements were conducted with a manual gain of 50, 30 flashes per well, and an integration time of 40 µs.

### Derivatization conditions

#### Derivatization of amino acid standards

Amino acid standard mixture was prepared with a final concentration of 5 μM for each amino acid. The derivatization reaction was initiated by adding the derivatization agent (10 μL, 20 mM in ACN) to a mixture comprising the amino acid standard solution (10 μL, 5 μM), sodium tetraborate buffer (40 µL, 5 mM, pH 9.0), and ACN (40 µL), yielding a final volume of 100 µL. The reaction mixture was incubated for 1 h at 25 °C and subsequently quenched with 1% aqueous formic acid containing 1 µM caffeine standard (100 μL). Prior to LC-MS analysis, samples were centrifuged at 25,000 × *g* for 20 min. Finally, the supernatant (0.1 μL) was injected into the LC-MS system.

Statistical evaluation of derivatization efficiency was performed using Python (3.13) with SciPy (1.16.1), statsmodels (0.14.5), and seaborn (0.13.2). The MS response for each agent was averaged across all five amino acids and technical replicates, using DNS as the reference. Fold changes relative to DNS and corresponding *p*-value were calculated, with statistical significance defined as *p* < 0.05.

#### Derivatization of plasma samples

Plasma sample (1 µL) was mixed with ACN (40 µL), sodium tetraborate buffer (47 μL, 5 mM, pH 9.0), norvaline (internal standard, 1 µL, 1 mM), and Deuterated amino acid standard mixture (1 µL). The derivatization was initiated by the addition of derivatization agent (10 µL, 20 mM in ACN), resulting in a final reaction volume of 100 µL. Following incubation for 1 h at 25 °C, the reaction was quenched with 4% formic acid in ACN:MeOH (1:1, v/v) containing 1 µM caffeine standard (100 µL). Samples were centrifuged at 25,000 × *g* for 20 min, and the supernatant was injected (0.1 μL) into the LC-MS.

### LC-MS analysis

The final method for the analysis of 20 proteinogenic amino acids in NIST plasma (derivatized with 6-CiQ-NHS) was performed on the Agilent 1290 Infinity II UHPLC system equipped with a diode-array detector (DAD) and coupled to the Agilent 6470 Triple Quadrupole system. Chromatographic separation was achieved on an ASTRA C18-AQ UHPLC column (100 × 2.1 mm; 2.0 µm, Chromservis) maintained at 40 °C, with a flow rate of 0.400 mL/min and an injection volume of 0.1 µL. The mobile phases consisted of (A) 0.1% FA in 5 mM ammonium formate and (B) 0.1% FA in ACN:MeOH (1:1 v/v). The gradient elution program was set as follows: 0 min (7% B); 1.0 min (7% B); 6.0 min (16% B); 12.9 min (35% B); 13 min (95% B); 14.0 min (95% B); 14.1 min (7% B); 16.0 min (7% B).

MS detection was performed in positive ESI mode using multiple reaction monitoring (MRM) acquisition (Table 1). The ESI source parameters were operated under the following conditions: drying gas temperature, 200 °C; drying gas flow, 6 L/min; nebulizer pressure, 40 psi; sheath gas temperature, 300 °C; sheath gas flow, 11 L/min; capillary voltage, 1750 V; and nozzle voltage, 0 V. Detailed LC-MS conditions for the preliminary agent screening and the DNS benchmark agent are provided in the Supporting Information (Chapter 2).

**Table 1:** MS parameters for 6-CiQ-derivatized amino acids and deuterium-labeled internal standards

Amino acid	Precursor ion [M+H]^+^	Product ion quantifier [M+H]^+^	Quantifier CE (eV)	Amino acid	Precursor ion [M+H]^+^	Product ion quantifier [M+H]^+^	Quantifier CE (eV)
Ala	245.1	199.1	20	Ala-[d_4_]	248.8	129.1	56
Arg	330.3	156.1	36	Arg-[d_7_]	337.2	128.2	56
Asn	288.4	271.1	4	Asn-[d_3_]	291.1	128.7	52
Asp	289.2	199.2	24	Asp-[d_3_]	292.1	186.2	32
Cys	432.1	190.1	24	Cys-[d_2_]	279.2	128.8	44
Gln	302.5	285.2	4	Gln-[d_5_]	306.9	129.1	60
Glu	303.1	156.2	32	Glu-[d_5_]	307.9	128.9	52
Gly	231.2	185.5	24	Gly-[d_2_]	232.7	128.9	44
His	311.1	156.1	28	His-[d_5_]	316.1	156.3	32
ILe	287.3	241.2	24	Ile-[d_10_]	297.1	128.8	56
Leu	287.3	185.3	8	Leu-[d_10_]	297.2	129.0	56
Lys	457.3	156.2	32	Lys-[d_8_]	464.6	128.2	60
Met	305.5	211.1	28	Met-[d_8_]	313.2	128.8	48
nVal^a^	274.1	228.1	24	-	-	-	-
Phe	321.3	156.2	8	Phe-[d_8_]	329.1	128.7	48
Pro	271.4	227.1	20	Pro-[d_7_]	278.1	234.1	24
Ser	261.1	151.2	20	Ser-[d_3_]	264.1	129.1	48
Thr	275.2	156.8	24	Thr-[d_5_]	280.1	129.1	48
Trp	360.5	156.2	24	-	-	-	-
Tyr	337.1	293.2	24	Tyr-[d_7_]	344.1	128.8	48
Val	273.3	199.2	28	Val-[d_8_]	281.3	129.1	52

^a^*n*Val was used as an internal standard

### Method validation

The LC-MS method for amino acid analysis was validated in accordance with the guidelines of the European Medicines Agency (EMA) [22]. Key analytical parameters were evaluated, including the limit of detection (LOD), limit of quantification (LOQ), carry-over, linearity, matrix effects, accuracy, precision, and selectivity.

#### Linearity, carry-over, and selectivity

Linearity was established using a 12-point calibration curve constructed via a linear regression model. Deuterium-labeled amino acid standards were prepared in both neat solvent and matrix (NIST plasma) by serial 2-fold dilution, ranging from the undiluted mixture down to a 2048-fold dilution. The exact concentrations at each calibration point varied for individual deuterium-labeled amino acids, reflecting their specific abundances in the Deuterated amino acid standard mixture (see Sect. Materials). Carry-over was assessed by injecting a blank sample immediately following the highest concentration standard used in the linearity study. Selectivity was confirmed by comparing plasma samples spiked with deuterium-labeled amino acid standards against unspiked blanks.

#### LOD, LOQ, and matrix effect

Method sensitivity was defined by the LOD and LOQ, calculated as the concentration corresponding to signal-to-noise (S/N) ratios of 3 and 10, respectively. For the LOD and LOQ, the standard deviation (SD) was required not to exceed 20% across measurements (*n* = 6). The matrix effect (ME) was evaluated by comparing the slopes of calibration curves prepared in the matrix (plasma) versus those in the solvent, using the equation: ME(%) = (Slope_matrix_/Slope_solvent_) × 100.

#### Method accuracy

Accuracy was determined at three concentration levels—low, medium, and high—using six independently prepared replicates (*n* = 6). The concentration levels were selected based on the deuterium-labeled standard with the lowest abundance in the Deuterated amino acid standard mixture, asparagine-[d_3_]. The low level was set near the standard LOD, while the high level corresponded to the fourth highest point of the calibration curve. The medium level was calculated as the midpoint between these two values. Specific concentrations for other amino acids varied according to their proportion in the Deuterated amino acid standard mixture. Data processing involved normalization of analyte responses to the signal of caffeine (external standard). For the purpose of this study, accuracy was defined based on the consistency of response between consecutive independent samples. Accuracy was then calculated as the percentage ratio of caffeine-normalized peak areas between consecutive independent samples (sample *n* versus sample *n*–1), expressed as Accuracy(%) = (NPA_n_/NPA_n-1_) × 100, where NPA represents the normalized peak area. The reported intra-day accuracy represents the mean value of these ratios within a single day. Inter-day accuracy was determined as the mean value across three independent days.

#### Method precision

Precision was assessed at the same concentration levels as accuracy. Intra-day precision was expressed as the relative standard deviation (RSD) and calculated as: RSD(%) = (SD/mean normalized peak area) × 100. Inter-day precision was reported as the average RSD across three separate days.

#### Amino acid quantification in plasma by MS response ratio

Quantification of the non-deuterated amino acids in plasma was performed using the ratio of their MS response to that of corresponding deuterium-labeled internal standards. To ensure the reliability of this ratio across the biological concentration range, a 5-point calibration curve was established via linear regression. This calibration was based on defined molar ratios of non-deuterated to constant concentration of deuterium-labeled standards (2:1, 1.5:1, 1:1, 0.5:1, and 0.1:1). To prepare these calibration standards, plasma samples were spiked with the corresponding mixtures of non-deuterated and deuterium-labeled amino acids, prepared by the protocol described in the protocol described in Sect. Derivatization of amino acid standards. Each calibration point was analyzed in six replicates.

## Results and discussion

### Design and rationale of derivatization agents

**Fig. 1:**
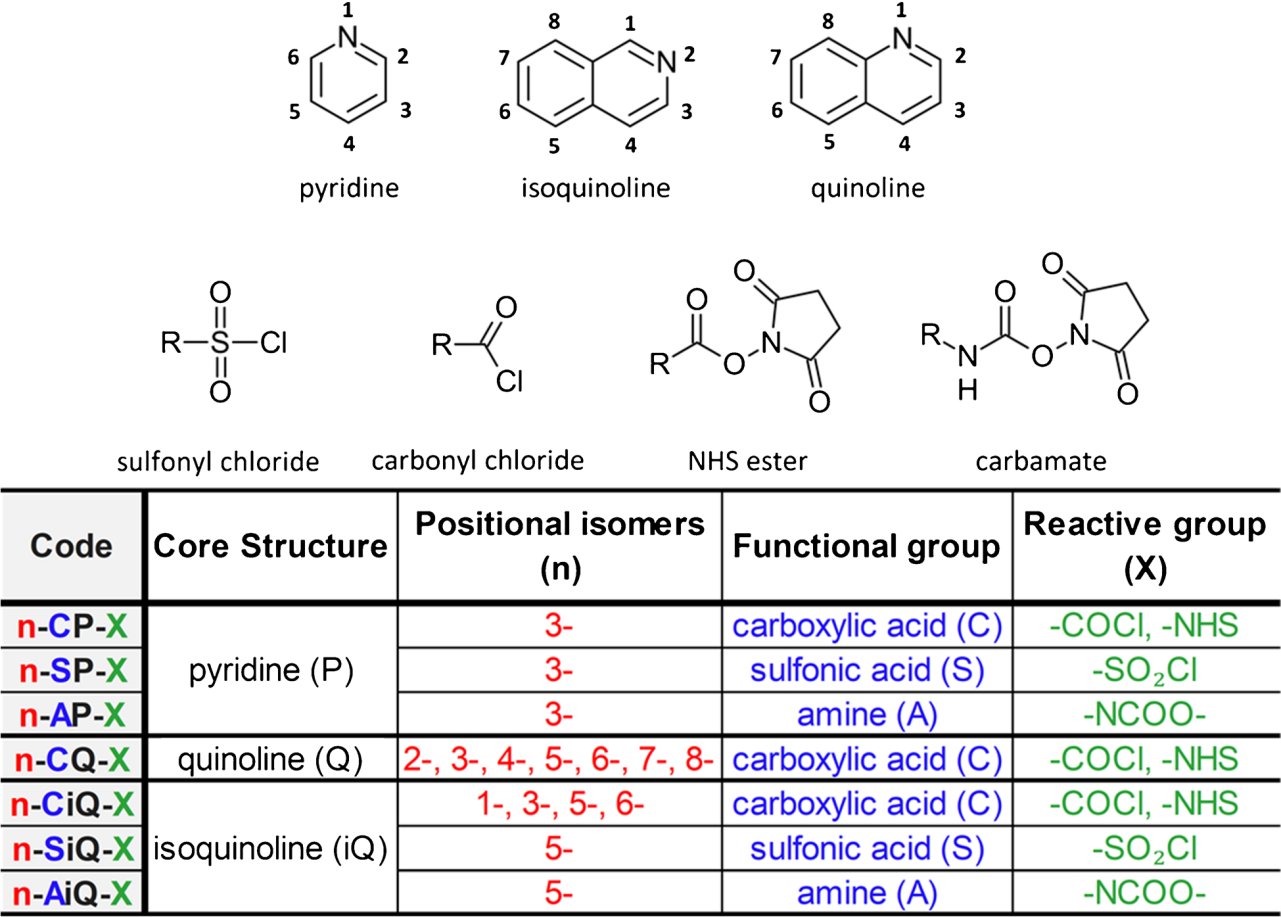
Design strategy of the derivatization agents: Summary of the utilized heterocyclic scaffolds and reactive groups, illustrating the nomenclature system

Based on our knowledge of heterocycles physical-chemical properties and previous works [10, 23, 24], three core structures with incorporated nitrogen atom as a protonatable moiety—pyridine, quinoline, and isoquinoline—were selected for evaluation (Fig. 1). The incorporated nitrogen atom was hypothesized to remain inert during derivatization, thereby preventing self-quenching or unwanted side reactions with the reactive group. This nitrogen atom could be protonated in the ESI, enhancing ionization efficiency in positive-ion mode. Moreover, its integration into the aromatic core prevents charge loss during MS fragmentation. Furthermore, the conjugation of the aromatic scaffold was expected to support UV absorption and FLD properties, enabling high detection sensitivity in LC-UV and LC-FLD analyses. Additionally, the aromatic core should increase lipophilicity, improving retention on RP columns. Finally, these scaffolds offer multiple substitution sites, allowing for a systematic investigation of the effect of positional isomerism on the analytical performance.

In terms of the reactive moiety, four reactive groups with known affinity for amines [10, 23, 24]—sulfonyl chloride, carbonyl chloride, NHS ester, and carbamate—were tested (Fig. 1). Although sulfonyl chlorides, carbonyl chlorides, and carbamates are common in commercial agent structures (e.g., DNS [25], AQC [16, 26], or Fmoc-Cl [19]), they often suffer from rapid hydrolysis. Therefore, NHS esters were selected to test the hypothesis whether they offer a superior balance between reactivity and stability. To ensure clarity, starting materials and prepared compounds were designated using a code following the form: “*Position* – *Aromatic core* – *Reactive moiety*”. For example, 6-CiQ-NHS denotes an isoquinoline core with a carboxylic acid function at position 6, activated as an NHS ester (Fig. 1).

### Synthesis of derivatization agents

A one-step synthetic protocol was successfully established for the majority of the designed agents. Carbonyl chlorides (Figure S19 i) were synthesized by treating the corresponding carboxylic acids with liquid thionyl chloride as a chlorinating agent at 70–75 °C. The reaction proceeded to quantitative conversion (Table S1) without the need for other solvents. The products were utilized without further purification. The synthesis of sulfonyl chlorides (Figure S19 ii) followed a similar protocol, using the respective sulfonic acid and thionyl chloride. However, a catalytic amount of DMF was required to enable the reaction. The products were purified via liquid-liquid extraction [27]. The sulfonyl chlorides were obtained in yields ranging from 21 to 74% yield (Table S1). In parallel, the NHS ester agents (Figure S19 iii) were synthesized by reacting the appropriate carboxylic acid precursor with DSC in the presence of triethylamine. These compounds required minimal workup and enabled efficient purification using isocratic chromatography, achieving yields ranging from 33 to 99% (Table S1). All prepared compounds were subsequently characterized and confirmed by MS and nuclear magnetic resonance (details in Supporting Information, Chapter 1).

The preparation of carbamate-functionalized agents proved challenging. Attempts to synthesize these compounds were unsuccessful due to the high reactivity of the forming product, which readily reacted with the nucleophilic amino group on the starting material (amino-pyridine (AP)/amino-isoquinoline (AiQ)) to form stable urea products (Figure S19 iv). Unfortunately, this undesired side reaction persisted despite performing the reaction at lower temperatures or varying the stoichiometry of the reaction components. These findings are consistent with the literature, which points to the high reactivity of carbamates [28]. Consequently, the carbamate pathway was excluded from further evaluation, and the study focused on the comparative assessment of carbonyl chlorides, sulfonyl chlorides, and NHS-based agents.

### Characterization of derivatization agents

#### Derivatization agent stability

The stability of derivatization agents is one of the important factors for determining their practical utility. Therefore, stability was evaluated under two distinct approaches. While the stability in solvent systems was assessed for all synthesized agents, long-term storage stability was evaluated for selected representatives of sulfonyl chloride, carbonyl chloride, and NHS ester agents. The long-term stability was assessed by storage at –20 °C under a nitrogen atmosphere. The carbonyl chloride–based agents exhibited the poorest stability, becoming unusable within 3 days. The sulfonyl chloride–based agents were moderately more stable but still degraded within seven days. In contrast, the NHS ester-based agents demonstrated superior long-term stability, remaining intact for over 1 year under identical conditions (Table S2).

**Fig. 2:**
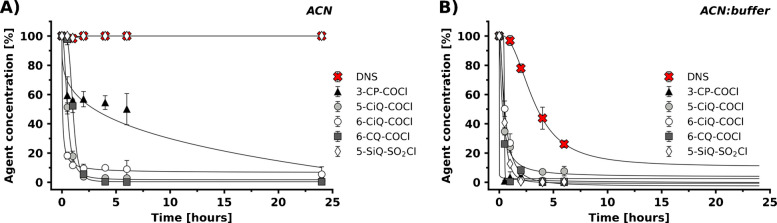
Stability of pyridine-, isoquinoline-, and quinoline-based derivatization agents containing sulfonyl chloride and carbonyl chloride groups, assessed in ACN (**A**) and a 1:1 (v/v) ACN:tetraborate buffer mixture (**B**), compared with the stability of DNS. The source data for this figure are provided in Table S3

The stability of agents in solution was investigated in two distinct solvents. Stock solution stability was tested in pure ACN. In contrast, a 1:1 (v/v) mixture of ACN and 5 mM sodium tetraborate buffer (pH 9.0) was employed to mimic the aqueous alkaline derivatization conditions. The commercially available DNS agent served as a benchmark. Results indicated that agents’ stability was correlated with the reactivity of the reactive groups. Carbonyl chloride–based agents exhibited the lowest overall stability in both tested solvents. In ACN, the initial concentration of these agents decreased by more than 85% within one hour (Fig. 2A). Notably, an even faster degradation rate was observed in the ACN:tetraborate mixture (Fig. 2B) with less than 50% of the initial concentration remaining after 30 min. These findings align with the high electrophilicity of the carbonyl carbon and the limited resonance stabilization typical for acyl chlorides. In contrast, the prepared sulfonyl chloride–based agent (5-SiQ-SO_2_Cl) showed higher stability in ACN. Such stability is likely due to the lower electrophilicity of the sulphur atom compared to the carbon atom, along with effective resonance stabilization [29, 30]. This was evidenced by an almost unchanged concentration within 24 h (Fig. 2A), indicating its suitability for preparing stable stock solutions. A different trend was observed in the alkaline conditions (ACN:tetraborate buffer mixture), where the 5-SiQ-SO_2_Cl agent degraded rapidly, losing more than 90% of its parent structure within 2 h (Fig. 2B). These findings contrast with the benchmark DNS agent. While similarly stable in ACN, DNS retained more than 75% of its initial concentration after 2 h in an alkaline environment. The variation between 5-SiQ-SO_2_Cl and DNS is probably attributed to differences in electron distribution within their respective core structures, which significantly impact the hydrolytic stability of the S-Cl bond [29, 31].

**Fig. 3:**
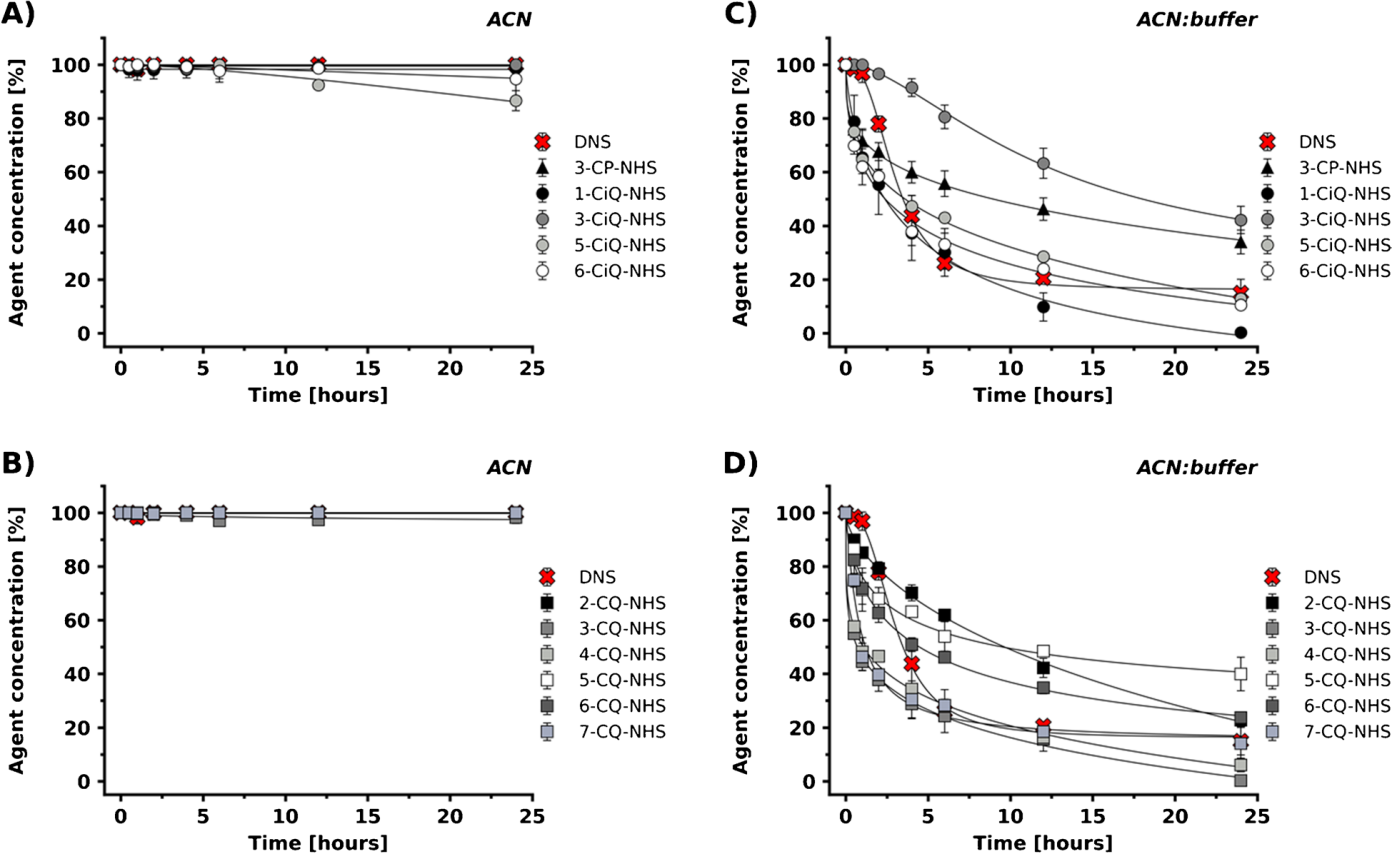
Stability of pyridine, isoquinoline (**A**), and quinoline (**B**) derivatives bearing NHS groups in ACN; and pyridine, isoquinoline (**C**), and quinoline (**D**) derivatives in ACN:tetraborate buffer (1:1, v/v), compared with DNS. The source data for this figure are provided in Tables S4-S6

NHS esters generally demonstrated good stability in ACN, with most pyridine, isoquinoline, and quinoline-based agents retaining over 95% of their initial concentration after 24 h (Fig. 3A, B). Stability decreased in the ACN:tetraborate buffer (Fig. 3C, D). This was expected due to the known hydrolysis of the NHS ester group in aqueous alkaline conditions [32–34]. However, the degradation rate remained manageable compared to carbonyl and sulfonyl chloride–based agents.

The impact of positional isomerism of the reactive group on stability was evident in both quinoline- and isoquinoline-NHS series in the alkaline system. Specifically, within the quinoline core, isomers such as 2-, 5-, and 6-CQ-NHS exhibited greater resistance to hydrolysis compared to others (e.g., 3-, 4-, and 7-CQ-NHS). A consistent trend was observed for whole isoquinoline series. These results suggest that the position of the NHS ester group on the heteroaromatic core influenced subtle electronic and steric effects [35], thereby playing a pivotal role in sensitivity to hydrolysis.

It is important to note that two derivatization agents displayed complete instability. 8-CQ-NHS was not detected in either solvent after dissolution. This observation suggests a rapid decomposition pathway potentially affected by steric strain or unfavorable electronic distribution in the core. Similarly, 3-SP-SO₂Cl proved completely unstable under the tested conditions. This behavior can be attributed to the high reactivity of the sulfonyl chloride group in this specific position [29, 34]. These findings highlight the importance of considering not only the reactive group but also the specific structural scaffold, including positional isomerism, when designing stable and effective derivatization agents.

#### Spectroscopic analysis of derivatization agents

**Fig. 4:**
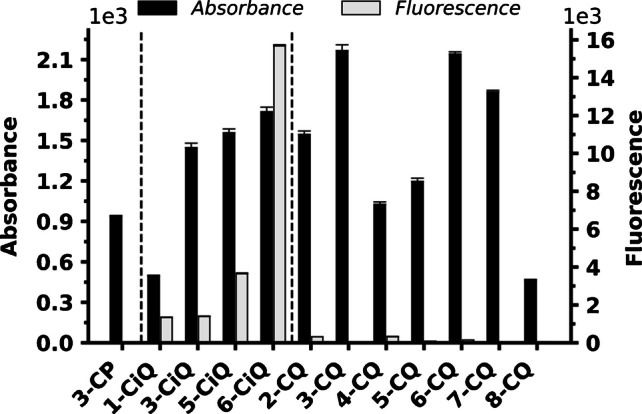
Comparison of absorbance and fluorescence intensities of the derivatization agents based on different heterocyclic cores and positional isomerism. Data were obtained from equimolar solutions measured at their respective absorption/emission maxima (λ_max_/Ex_max_). The source data for this figure are provided in Table S7

Despite the primary focus on MS analysis, the aromatic conjugation of the scaffolds provides optical properties enabling alternative detection methods. The systematic evaluation of agents revealed that spectroscopic characteristics were modulated by the type of heterocyclic core and the positional isomerism of the reactive group (Fig. 4). While the majority of agents exhibited distinct UV absorbance, the intensity varied. Quinoline agent derivatives 3-CQ and 6-CQ demonstrated the highest absorbance values, whereas 1-CiQ and 8-CQ displayed the lowest. Furthermore, the isoquinoline scaffold showed superior potential for FLD detection, with 6-CiQ-NHS identified as a particularly promising candidate. These agents possessed both chromophoric and fluorophoric properties, establishing them as a versatile option for diverse analytical workflows.

#### MS analysis of derivatized amino acids

**Fig. 5:**
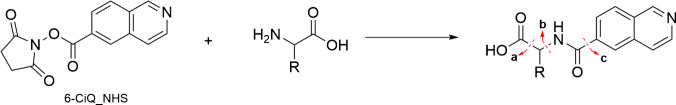
Reaction scheme of amino acid derivatization with 6-CiQ-NHS and proposed fragmentation pathways of the resulting derivatives in positive-ion mode

The fragmentation behavior of the derivatized amino acids was studied to establish transitions for MRM analysis (Table S8). A representative mixture of six amino acids (alanine, cysteine, histidine, serine, and tyrosine) was derivatized with the synthesized agents. This analysis revealed a consistent fragmentation pattern characterized by three distinct pathways across all agents (Fig. 5). First, a neutral loss of 46 m*/z* was observed, corresponding to the cleavage of the carboxylic acid (Fig. 5(a)). Additionally, the cleavage of the amino acid side chain yielded a fragment attributed to the amide or sulfonamide of the derivatization agent (Fig. 5(b)). However, the most intense fragment represented the positively charged heterocyclic core of the specific derivatization agent (Fig. 5(c)).

**Fig. 6:**
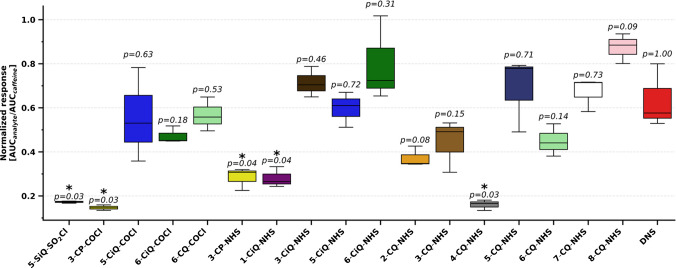
Comparison of average MS response of derivatized amino acids with synthesized agents expressed as the average (*n* = 6) normalized MS response to the caffeine standard. The source data for this figure are provided in Table S9

Following the establishment of MRM fragments, a comparative LC-MS response analysis was conducted for all prepared agents (Table S8). To control for analytical variations, two standards were employed. The norvaline (internal standard) monitored the derivatization efficiency. The caffeine (external standard) was used to normalize MS fluctuations during the analysis. The average MS response intensity of derivatized amino acids for each agent was summarized to provide a clear evaluation of their performance (Fig. 6). The commercially available DNS agent served as a benchmark for evaluation.

The majority of agents successfully derivatized the tested amino acid standards. A notable exception was 3-SP-SO_2_Cl, which (in accordance with its instability) failed to yield reaction products with any of the analytes. Among the evaluated amino acids, cysteine presented a specific analytical challenge. It exhibited negligible derivatization yields with several agents (detailed data are provided in Table S9). This difficulty may be attributed to the known instability of the free cysteine and its susceptibility to oxidation to form the disulfide-bonded dimer, cystine [36, 37] (although detailed confirmation was beyond the scope of this work). Furthermore, the proximity of the thiol moiety to the amino group may modulate the nucleophilicity of the amine through electronic effects and consequently impede the derivatization reaction.

A key observation was the influence of the positional isomerism of the agents' reactive group on the MS intensity of derivatized products (Fig. 6). Agents 6-CiQ-NHS and 8-CQ-NHS consistently exhibited the highest performance, showing responses 1.12- to 1.38-fold higher than the DNS benchmark. Other high-performing agents included 3-CiQ-NHS, 5-CiQ-NHS, and several quinoline derivatives such as 5-CQ-NHS and 7-CQ-NHS. Additionally, carbonyl chlorides 5-CiQ-COCl and 6-CQ-COCl demonstrated competitive sensitivity. In contrast, agents based on the pyridine core (3-CP-COCl, 3-CP-NHS) and specific isoquinoline and quinoline isomers (e.g., 1-CiQ-NHS, 2-CQ-NHS, 4-CQ-NHS, and 5-SiQ-SO_2_Cl) showed a statistically lower MS response compared to DNS (*p* < 0.05).

#### Reaction kinetics

**Fig. 7:**
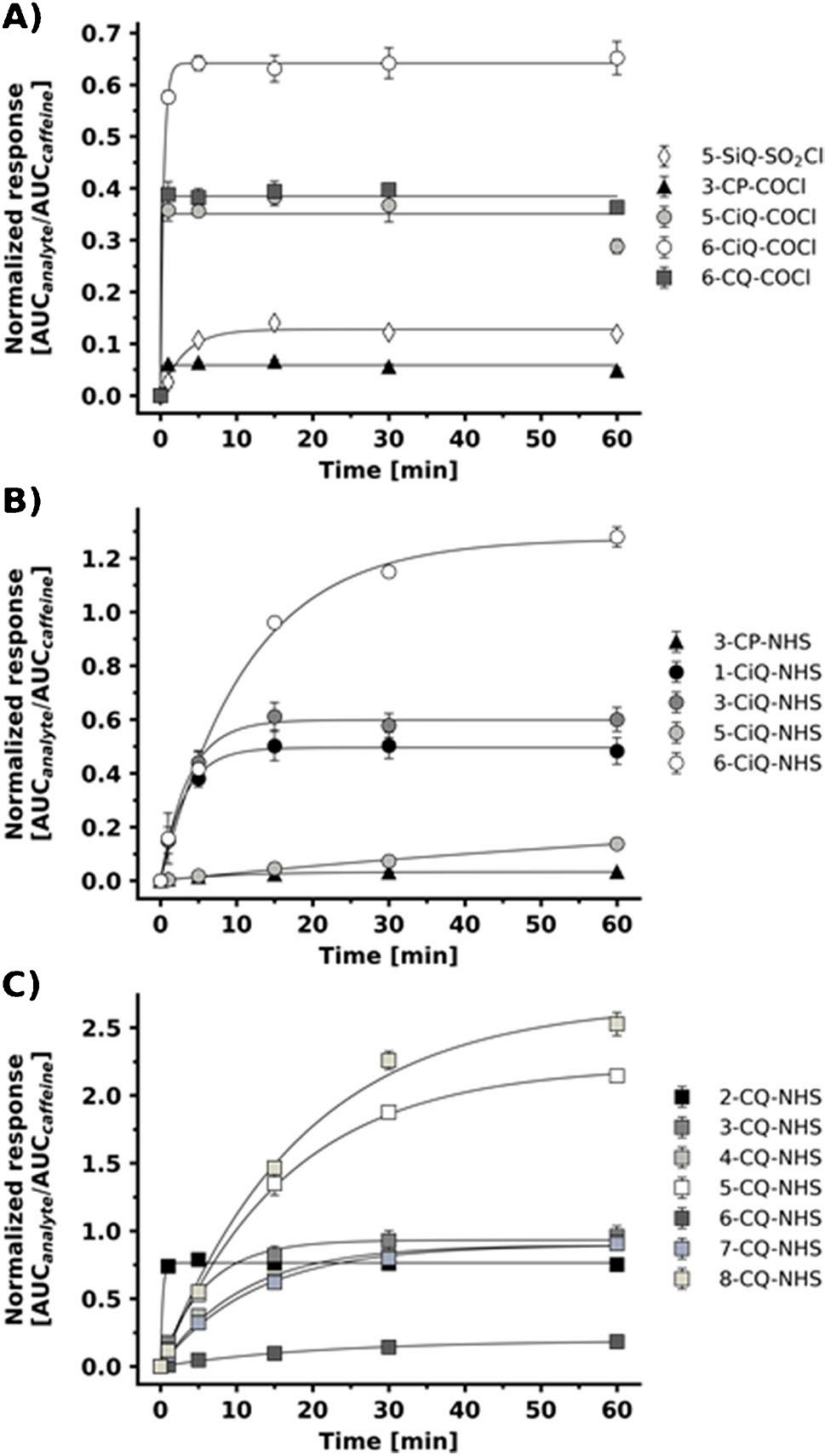
Kinetic profiles of serine derivatization with prepared agents featuring different functional groups: **A** thionyl chlorides and carbonyl chlorides, **B** pyridine and isoquinoline-NHS esters, and **C** quinoline-NHS esters. The source data for this figure are provided in Table S10

To further investigate the reactivity of synthesized agents, kinetic profiles were evaluated using the representative mixture of six amino acids (alanine, cysteine, histidine, serine, and tyrosine). Since the kinetic trends were consistent across all tested amino acids, serine was chosen as a model to illustrate the kinetic behavior of the agents (Fig. 7). As expected, the reaction kinetics varied among the tested agents depending on the utilized reactive group and positional isomerism. The sulfonyl chloride agent exhibited rapid kinetics, reaching maximum product formation within 10 min (Fig. 7A). The carbonyl chloride–based agents demonstrated even higher reactivity, achieving complete reaction within 5 min. However, as discussed in Sect. Derivatization agent stability, the high reactivity of chlorides is compromised by their lower solvent stability. In contrast, the NHS ester agents, isoquinoline and quinoline series (Fig. 7B, C), generally displayed lower kinetic rates, which were compensated for by their superior solvent stability.

Beyond the reactive group type, its specific position on the core played a crucial role. Kinetic data revealed that the proximity of the reactive group to the electron-withdrawing ring nitrogen likely enhances local electrophilicity, thereby accelerating the reaction. For instance, in the isoquinoline series, 1-CiQ-NHS and 3-CiQ-NHS (positions proximal to nitrogen) reached maximum product formation within 15 min. In contrast, the distal positions from nitrogen, 5-CiQ-NHS and 6-CiQ-NHS isomers, required 60-min incubation. A similar trend was observed among the quinoline series. Isomer 2-CQ-NHS (proximal to nitrogen) reached its maximum product concentration in 5 min, whereas more distal positions 3-, 4-, and 7-CQ-NHS isomers achieved maximum within 30 min. Isomers 5-CQ-NHS and 6-CQ-NHS required 60 min, mirroring the behavior of their isoquinoline isomers.

The impact of agent concentration on the reaction was evaluated by comparing 5 mM and 20 mM agent solutions. Although the higher concentration resulted in a modest increase in reaction efficiency (approximately 10%) for standard solutions, it was selected for the final protocol. This molar excess is expected to drive the reaction equilibrium and compensate for agent hydrolysis in the aqueous alkaline buffer. Furthermore, it ensures reliable derivatization in complex biological matrices (e.g., plasma) by overcoming competition from endogenous nucleophiles, such as proteins, peptides, or biogenic amines.

#### Chromatographic behavior of derivatives

**Fig. 8:**
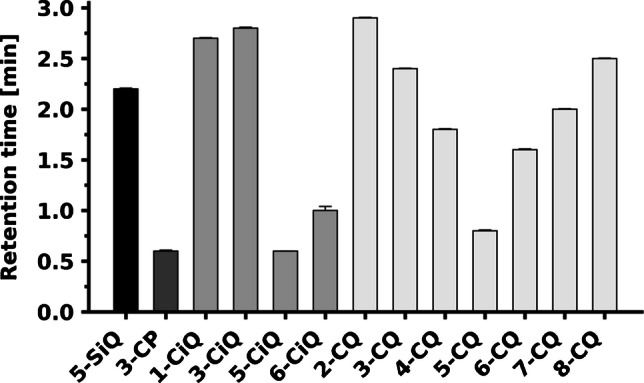
Influence of the heterocyclic core (pyridine, isoquinoline, quinoline), type of functional group (amide or sulfonamide), and positional isomerism on the retention time of serine derivatives. The source data for this figure are provided in Table S11

The influence of the heterocyclic core and the final linkage group between agent and amino acid (the amide or sulfonamide structure) on the retention time was evaluated for all prepared agents (Fig. 8). As expected, derivatives formed from sulfonyl chloride precursors (sulfonamides) generally exhibited longer retention times. In contrast, those formed from NHS ester or carbonyl chloride precursors (amides) eluted earlier. This observation reflects the higher inherent lipophilicity of the sulfonamide group [30].

Positional isomerism within the quinoline and isoquinoline series significantly affected also retention behavior. A clear dependence was observed between retention and the distance of the linkage group from the nitrogen atom in the agent core. In the quinoline series, derivatives at the 2-position (adjacent to nitrogen) and the 8-position (peri-position, in close proximity to nitrogen) exhibited the highest retention. Conversely, the remote 5-CQ-derivative eluted first. A similar trend was obtained in the isoquinoline series. Here, 1- and 3-CiQ-derivatives—both surrounding the nitrogen atom—displayed the longest retention times, while the 5-CiQ-derivative exhibited the shortest (Fig. 8). These shifts in retention times suggest that the strong and weak retention of individual isomers directly reflects variations in the overall molecular polarity. The specific position of the reactive group relative to the nitrogen atom influences the electron distribution and the overall dipole moment. Consequently, this modulates the molecule’s lipophilicity and its interaction with the stationary phase [35]. Based on a complex evaluation comprising stability (Sect. Derivatization agent stability), MS sensitivity (Sect. MS analysis of derivatized amino acids), and chromatographic performance, 3-CiQ-NHS, 6-CiQ-NHS, 3-CQ-NHS, and 6-CQ-NHS were identified as the most promising candidates for further detailed investigation.

### Final analytical method development

#### Influence of buffer pH and reaction temperature on conversion efficiency

**Fig. 9:**
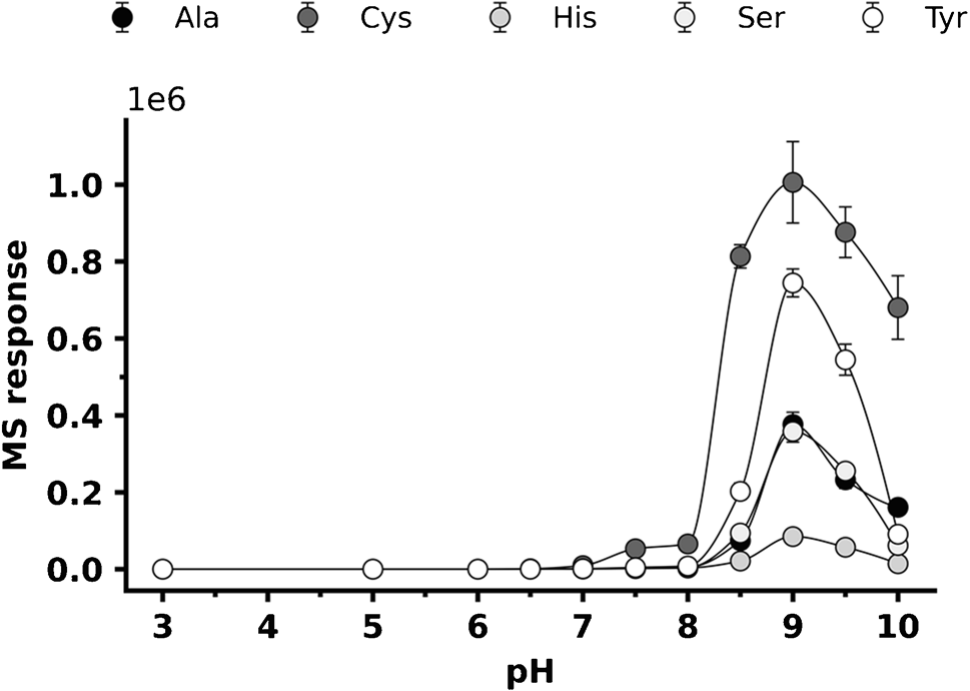
Effect of sodium tetraborate buffer pH on the derivatization efficiency of the 6-CiQ-NHS agent. The source data for this figure are provided in Table S12

Following the selection of the four most promising NHS ester agents, key reaction parameters (including buffer composition, pH, and temperature) were evaluated to maximize amino acid conversion efficiency. Two buffers, sodium tetraborate (5 mM, pH 9.0) and sodium bicarbonate (5 mM, pH 9.0), were directly compared. Volatile, MS-compatible ammonium buffers were avoided due to the potential for ammonia to react with the reactive group of NHS ester. Consistent with previous reports [38], the tetraborate buffer proved superior. Specifically, it yielded an average MS response for derivatized products 1.7-fold higher than that obtained with the bicarbonate buffer. Consequently, sodium tetraborate was selected for all subsequent experiments. The influence of pH on reaction efficiency was subsequently assessed over the range of 7.0–10.0 (Fig. 9, Table S12). Maximum MS responses were consistently observed at pH 9.0, which is in good agreement with established literature protocols for NHS derivatization [39].

Finally, the impact of temperature on derivatization reaction was evaluated at 25, 40, and 60 °C with a 60-min incubation. No statistically significant differences in conversion efficiency were observed across the tested temperatures (Figure S20, Table S13). A temperature of 25 °C provided a satisfactory balance between reaction rate and minimizing potential agent degradation. Importantly, the results indicate that the method is robust, as minor temperature fluctuations do not substantially affect derivatization efficiency.

#### Chromatographic separation

**Fig. 10:**
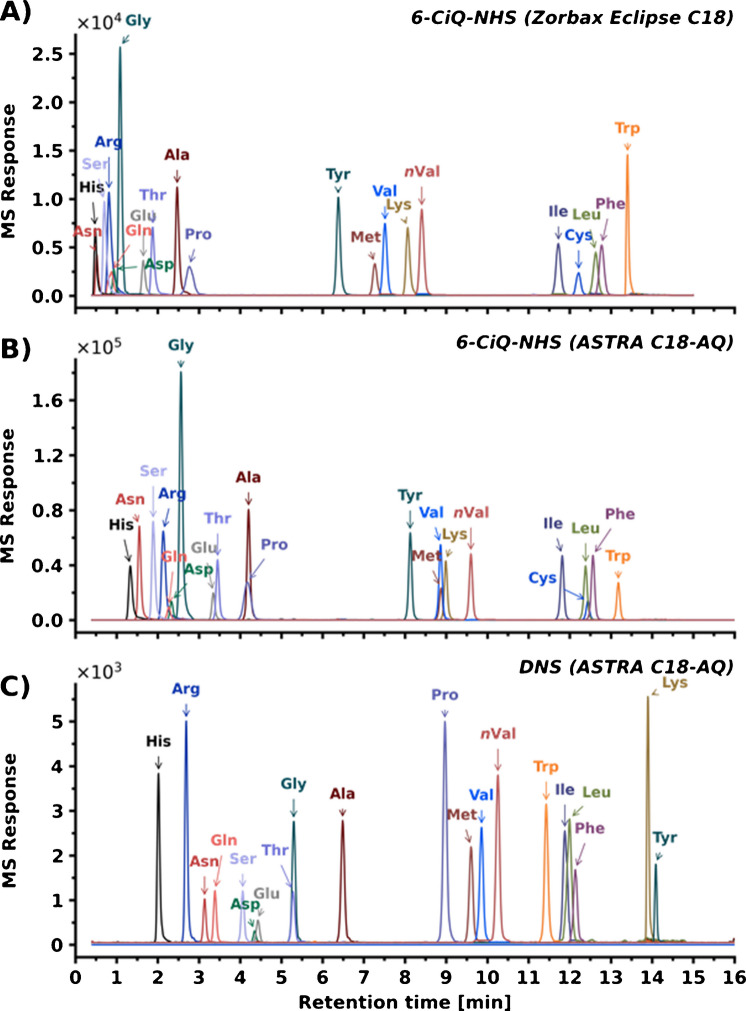
Chromatographic separation of 6-CiQ-NHS amino acid derivatives on Zorbax Eclipse C18 (**A**) and ASTRA C18-AQ (**B**) columns, and DNS amino acid derivatives’ separation on the ASTRA C18-AQ column (**C**)

Chromatographic separation was performed using two distinct C_18_ columns (Fig. 10, Figure S21): a fully endcapped Zorbax Eclipse Plus C18 (50 × 2.1 mm, 1.8 µm) and a polar-modified ASTRA C18-AQ (100 × 2.1 mm, 2.0 µm). The ASTRA C18-AQ column was selected for its compatibility with highly aqueous mobile phases and for its capacity for π-π interactions, providing an additional separation mechanism to standard hydrophobic partitioning. The primary objective was to achieve the baseline resolution of 21 amino acids, with particular focus on the isomeric pairs (isoleucine/leucine and valine/norvaline).

Evaluation of the 3-substituted agent isomers revealed differences in chromatographic performance. The 3-CiQ-NHS failed to resolve the isomeric pair on the shorter column (Figure S21A). Although a tendency toward separation was observed on the longer column (Figure S21B), the resolution remained insufficient. In contrast, the quinoline analogue 3-CQ-NHS achieved adequate separation on both columns (Figure S21C, D). The 6-substituted derivatization agents (both 6-CiQ-NHS and 6-CQ-NHS) successfully resolved isomer pairs on both tested columns (Figure S21E, F). Nevertheless, utilizing the shorter Zorbax Eclipse Plus C18 column (Fig. 10A) resulted in compromised resolution of early-eluting derivatized amino acids compared to the longer ASTRA C18-AQ column (Fig. 10B). Therefore, the ASTRA C18-AQ column was selected for further method development.

For direct comparison, the benchmark DNS agent was also analyzed on the ASTRA C18-AQ column (Fig. 10C). Contrary to the 3-CQ-NHS, 6-CiQ-NHS, and 6-CQ-NHS agents, the DNS agent was incapable of separating the isoleucine/leucine isomers under the tested conditions. Furthermore, the higher reactivity of the sulfonyl chloride group of DNS led to the formation of doubly labeled products for lysine and tyrosine. The corresponding single-labeled products were not observed for these amino acids. This contrasts with the previous agents, which selectively yielded mono-derivatized products. The only exception was lysine, where the formation of doubly labelled product was analogous to that of DNS. Ultimately, 6-CiQ-NHS was selected as the final derivatization agent for method validation. Although selected quinoline derivatives (3-CQ-NHS and 6-CQ-NHS) demonstrated satisfactory chromatographic resolution, they exhibited lower MS responses compared to 6-CiQ-NHS. Consequently, 6-CiQ-NHS was chosen based on its superior overall performance assessment, combining good chromatographic resolution with the highest MS response, stability, and favorable spectral properties.

### Method validation and LC-MS analysis of plasma samples

#### Derivatization of deuterium-labeled standards and kinetic isotope effects

Derivatization of deuterium-labeled amino acids was investigated to enable the use of isotopically labeled internal standards for method validation and accurate quantification. The fragmentation patterns of deuterium-labeled analogues were found to be consistent with their non-deuterated counterparts. The observed mass shifts corresponded to the number of incorporated deuterium atoms. The exceptions were the fragment ions at *m*/*z* 129 and *m*/*z* 156, which originate from the 6-CiQ-NHS heterocyclic core. These fragments remained unchanged (Table 1). However, a challenge was encountered when the expected molecular ion for some derivatized deuterium-labeled amino acids was notably absent or exhibited negligible intensity. Specifically, tryptophan-[d_8_] showed markedly poor conversion efficiency and was not detected under the established conditions. A similar trend was observed with glycine isotopes. While glycine-[d_2_] reacted efficiently, the fully deuterated glycine-[d_5_] yielded a significantly reduced MS response. These results suggest a potential kinetic isotope effect (KIE) [40]. The hypothesis is that the extensive deuteration in glycine-[d_5_] and tryptophan-[d_8_] may impede the proton abstraction from the amino group. Since this abstraction is essential for the nucleophilic attack on the NHS ester carbonyl group, the overall reaction rate is kinetically limited. It is also documented that KIEs can influence the reaction kinetics even when deuterium is not directly involved in the bond undergoing cleavage [40, 41].

A similar limitation was observed with the benchmark DNS agent. While the fragmentation pattern of both DNS derivatized non-deuterated and deuterium-labeled amino acids was consistent with literature reports [23, 42, 43], the derivatization efficiency was compromised for six deuterium-labeled standards (asparagine-[d_3_], cysteine-[d_2_], methionine-[d_8_], tryptophan-[d_8_], lysine-[d_8_], and tyrosine-[d_7_]). For these standards, the expected molecular ion was either not observed or exhibited very low MS intensity. To our knowledge, this specific reactivity limitation regarding highly deuterated standards has not been comprehensively addressed in previous studies. This is likely because many protocols rely on ^13^C or ^15^N isotopically labeled standards [14]. Our observations strongly suggest that extensive deuteration can negatively impact the efficiency of both NHS esters and sulfonyl chloride derivatization chemistries.

#### Stability of derivatized analytes

The stability of 6-CiQ-derivatized amino acids and their deuterium-labeled analogues was assessed under conditions designed to mimic practical analytical workflows (Tables S14–S19). Across all tested conditions (including short-term storage at ambient temperature, long-term freezing at −80 °C, and stability through an evaporation-reconstitution cycle), the majority of derivatives demonstrated good stability. The decrease in MS signal intensity for most of the analytes remained below 10% under these conditions. Exceptions were observed for proline and glutamate derivatives, which exhibited lower stability. For instance, after 24 h at ambient temperature, their signal intensities dropped by more than 20%. Proline proved to be the most labile analyte, showing a 31% signal reduction even during storage at −80 °C. Furthermore, the evaporation-reconstitution step led to pronounced degradation (19–38%) for proline, glutamate, and cysteine derivatives.

A comparable stability profile was observed for the deuterium-labeled amino acid derivatives. Following 24-h storage at laboratory temperature, most deuterium-labeled amino acids showed a signal decrease of less than 20%. After storage at −80 °C, the reduction in signal intensity was typically below 13%, with only lysine-[d_8_] exhibiting a decrease of 23%. Stability through the evaporation and reconstitution step was generally high, with signal exceeding 95%. The only exceptions were asparagine-[d_3_], methionine-[d_8_], and phenylalanine-[d_8_], which retained approximately 80% of their original signal.

#### Method validation and performance characteristics

The LC-MS method for the quantification of 6-CiQ-NHS-derivatized amino acids in human plasma was validated. Key validation parameters—linearity, LOD and LOQ, accuracy, precision, and matrix effects—are summarized in Table 2 and Table 3. Linearity was excellent for all characterized deuterium-labeled amino acids, yielding coefficients of determination (*R*^2^) exceeding 0.9950 across a broad concentration range of 1.5–5550 nM. Evaluation of method sensitivity revealed that 6-CiQ-NHS exhibited superior LOD and LOQ values compared to DNS for the majority of amino acids. Only threonine-[d_5_] and phenylalanine-[d_8_] showed comparable performance with DNS. On average, LODs obtained with 6-CiQ-NHS were approximately 6-fold lower than those achieved with DNS in this study. These results demonstrate superior ionization and robust fragmentation properties of the isoquinoline core, as well as suggesting improved derivatization efficiency compared to DNS. The absolute LOD for 6-CiQ-NHS ranged from 0.23 to 6.33 nM (normalized to a 1 µL injection), indicating that trace-level detection is feasible with minimal sample volume.

Placing these findings into perspective was challenging as literature-reported LODs differ widely depending on instrumentation and analytical protocol (especially sample preparation and injection volume). To allow for more or less consistent comparison, published values were also normalized to a 1 µL injection and compared with the measured values. Analysis of these published data revealed that highly sensitive methods achieved LODs between 0.008 and 348 nM, whereas less sensitive protocols ranged above 10 µM [43–47]. While 6-CiQ-NHS does not achieve the lowest reported LODs, it offers highly competitive sensitivity balanced by superior chemical stability, ease of synthesis, and good separation efficiency.

Matrix effects were evaluated by comparing the slopes of calibration curves prepared in plasma and a neat buffer solvent. The results revealed variability with matrix effect ratios ranging from 11 to 233% (comparison of calibration curves is shown in Figure S22). This highlights the necessity of using isotopically labeled internal standards to compensate for matrix-induced or reduced ionization in complex biological samples. Intra-day and inter-day accuracy and precision were assessed at three concentration levels (low, medium, and high). Accuracy values ranged from 90 to 140%, while intra-day precision and inter-day precision were 1–36% and 1–34%, respectively. Although some variability was observed at extreme concentrations, the method satisfied typical bioanalytical acceptance criteria [22]. This reliability, together with the achieved low LODs, confirms that derivatization with 6-CiQ-NHS enables sensitive and quantitative amino acid profiling. Its performance, coupled with the agent’s chemical stability and straightforward synthesis, positions it as a valuable alternative tool for routine LC-MS amino acid analyses.

**Table 2:** Summary of validation parameters for the 6-CiQ-NHS LC-MS method for amino acid analysis, including linearity, LOD and LOQ, accuracy, precision, and matrix effects

Amino acid	Retention time (min)	Calibration curve	*R*^2^	Matrix effect (%)	Selectivity (%)	Carryover (%)	Intra-day						Inter-day					
							Accuracy (%)			Precision (%)			Accuracy (%)			Precision (%)		
							L	M	H	L	M	H	L	M	H	L	M	H
Ala-[d_4_]	4.16	*y* = 2.3527*x* − 0.1786	0.9972	128	100	0.1	100	95	97	3	4	2	108	100	98	10	3	2
Arg-[d_7_]	2.05	*y* = 2.3218*x* − 0.0485	0.9976	221	100	0.2	103	91	98	9	5	1	106	98	97	7	4	1
Asn-[d_3_]	1.51	*y* = 0.0833*x* − 0.0001	0.9981	23	100	0.7	95	93	98	6	8	1	123	100	98	18	6	3
Asp-[d_3_]	2.31	*y* = 0.1156*x* − 0.0004	0.9997	52	100	0.2	106	91	91	14	2	4	140	102	96	26	11	4
Cys-[d_2_]	3.42	*y* = 0.0894*x* − 0.0003	0.9994	42	100	1.1	95	98	96	9	7	3	113	101	98	28	9	5
Gln-[d_5_]	2.25	*y* = 0.8864*x* − 0.0647	0.9972	120	100	0.1	101	94	95	1	4	2	108	100	97	11	3	2
Glu-[d_5_]	3.32	*y* = 0.4453*x* − 0.0055	0.9992	68	100	0.3	98	93	93	6	2	3	111	100	97	14	5	2
Gly-[d_2_]	2.54	*y* = 1.786*x* − 0.1047	0.9954	75	100	0.4	99	95	95	6	5	2	107	100	97	11	3	2
His-[d_5_]	1.19	*y* = 1.7544*x* − 0.0373	0.9957	233	100	0.3	101	92	97	5	5	1	103	96	97	10	4	2
Ile-[d_10_]	11.67	*y* = 0.4834*x* − 0.0081	0.9979	42	100	0.3	118	95	95	7	8	4	110	98	96	7	5	2
Leu-[d_10_]	12.25	*y* = 1.6114*x* − 0.0435	0.9975	110	100	0.1	98	93	95	3	5	3	104	100	98	9	4	2
Lys-[d_8_]	8.93	*y* = 0.0618*x* − 0.0023	0.9984	120	100	1.1	106	93	100	24	6	5	102	96	100	34	8	4
Met-[d_8_]	8.74	*y* = 0.4171*x* − 0.002	0.9989	55	100	0.3	114	96	97	16	5	2	114	102	97	18	5	3
Phe-[d_8_]	12.44	*y* = 0.1965*x* − 0.0005	0.9965	20	100	1.1	108	94	99	36	10	5	110	100	100	27	9	3
Pro-[d_7_]	4.11	*y* = 0.1161*x* − 0.0026	0.9988	11	100	0.2	100	90	97	8	4	4	109	99	98	22	4	3
Ser-[d_3_]	1.88	*y* = 2.1584*x* − 0.0293	0.9983	148	100	0.2	102	93	98	4	2	1	107	100	98	10	3	2
Thr-[d_5_]	3.41	*y* = 1.2931*x* − 0.32	0.9988	138	100	0.1	104	93	97	2	2	2	112	100	99	10	4	2
Tyr-[d_7_]	8.05	*y* = 0.3712*x* − 0.0042	0.9986	53	100	0.4	99	92	97	9	4	2	108	100	99	16	4	1
Val-[d_8_]	8.74	*y* = 2.1565*x* − 0.0842	0.9974	129	100	0.1	100	92	96	4	2	3	106	99	98	8	2	2

*L*, low; *M*, medium; *H*, high

Trp-[d_8_] was not detected

**Table 3:** Comparison of LOD and LOQ for deuterium-labeled amino acids derivatized with 6-CiQ-NHS and the commercially available DNS agent

	6-CiQ-NHS			DNS		Ref. LOD range [42–46]^a^
Amino acid	Linear range (nM)	LOD (nM)	LOQ (nM)	LOD (nM)	LOQ (nM)	LOD (nM)
Ala-[d_4_]	32.0–4100.0	8.0 ± 0.4	32.0 ± 2.6	64.1 ± 1.9	256.3 ± 7.9	0.032–348.000
Arg-[d_7_]	9.4–1200.0	2.3 ± 0.2	18.8 ± 1.9	4.7 ± 0.8	18.8 ± 2.7	0.020–24.900
Asn-[d_3_]	18.8–1200.0	18.8 ± 3.0	150.0 ± 7.3	-	-	0.158–166.500
Asp-[d_3_]	15.6–500.0	31.3 ± 4.5	62.5 ± 5.0	62.5 ± 6.3	250.0 ± 5.2	0.267–232.800
Cys-[d_2_]	4.7–600.0	18.8 ± 3.1	37.5 ± 3.3	-	-	6.000–204.000
Gln-[d_5_]	10.8–5550.0	10.8 ± 1.8	43.4 ± 2.0	43.4 ± 5.3	173.4 ± 6.3	1.200–12.400
Glu-[d_5_]	6.3–1600.0	25.0 ± 2.9	100.0 ± 1.9	200.0 ± 7.8	400.0 ± 10.2	0.540–13.200
Gly-[d_2_]	7.4–3800.0	14.8 ± 0.9	59.4 ± 2.7	29.7 ± 3.8	118.8 ± 9.7	0.018–252.600
His-[d_5_]	4.9–1250.0	9.8 ± 0.7	78.1 ± 4.2	312.5 ± 7.4	625.0 ± 10.4	0.030–35.400
Ile-[d_10_]	15.2–975.0	7.6 ± 0.4	121.9 ± 4.1	60.9 ± 7.9	243.8 ± 6.0	0.008–13.400
Leu-[d_10_]	4.2–2125.0	16.6 ± 1.8	33.2 ± 2.9	66.4 ± 6.8	132.8 ± 6.4	0.009–51.900
Lys-[d_8_]	27.3–1750.0	27.3 ± 2.5	109.4 ± 5.6	-	-	0.061–76.500
Met-[d_8_]	4.7–600.0	9.4 ± 0.9	37.5 ± 2.1	-	-	0.027–30.400
Phe-[d_8_]	17.2–1100.0	17.2 ± 2.3	137.5 ± 4.3	34.4 ± 4.7	137.5 ± 7.1	0.058–34.500
Pro-[d_7_]	32.8–2100.0	8.2 ± 0.8	32.8 ± 3.4	32.8 ± 4.7	131.3 ± 7.4	0.030–23.400
Ser-[d_3_]	1.5–1500.0	23.4 ± 1.3	46.9 ± 4.6	93.8 ± 7.2	187.5 ± 7.9	0.145–277.200
Thr-[d_5_]	15.8–2025.0	63.3 ± 3.4	253.1 ± 4.8	63.3 ± 8.9	253.1 ± 7.2	0.012–174.900
Tyr-[d_7_]	4.4–1125.0	4.4 ± 0.6	35.2 ± 2.5	-	-	0.020–38.700
Val-[d_8_]	1.9–3900.0	7.6 ± 0.8	30.5 ± 1.5	60.9 ± 6.4	243.8 ± 7.8	0.032–14.700

Trp-[d_8_] was not detected

^a^Experimental LODs refer to deuterium-labeled standards; literature ranges refer to non-deuterated amino acids

#### Amino acid quantification in certified reference material

The isotope dilution strategy was employed to quantify the concentration of 6-CiQ-derivatized amino acids in the plasma matrix. This approach utilizes the peak area ratio of each target amino acid to its corresponding 6-CiQ-deuterium-labeled standard. This effectively compensated for matrix effects (ionization suppression or enhancement) and variability in derivatization efficiency. Calibration curves were constructed across five concentration points. Linear regression analysis confirmed a linear response for all calibration curves, with *R*^2^ values exceeding 0.9924. Minor deviations were observed for cysteine (*R*^2^ = 0.9771), glutamate (*R*^2^ = 0.9880), and serine (*R*^2^ = 0.9865) (Figure S23). For the majority of analytes, the corresponding deuterium-labeled internal standard was used to ensure precise quantification. However, as discussed in Sect. “Derivatization of deuterium-labeled standards and kinetic isotope effects” cysteine-[d_2_] and tryptophan-[d_8_] were found unsuitable due to insufficient derivatization efficiency. Therefore, alternative internal standards with similar retention times were selected (methionine-[d_8_] was used for cysteine and isoleucine-[d_10_] for tryptophan).

The accuracy of the method was confirmed by analyzing the NIST Standard Reference Material (SRM) 1950 plasma. Table 4 summarizes the experimental molar concentrations compared with the certified and non-certified reference values provided in the SRM Certificate of Analysis [48, 49]. With the notable exception of glycine, the measured concentrations demonstrated good accuracy, with a maximum difference of approximately 30% of the reference values. However, these differences are consistent with findings from independent studies by Xue [50] and Gray [51], who reported similar deviations for amino acids, including glycine, in the NIST SRM 1950 reference plasma sample. These findings highlight the persistent challenges in the accurate quantification of small, polar amino acids in complex matrices [52]. To confirm the method reproducibility, the mean coefficient of variation (CV) was determined from a total of nine measurements (three independent derivatization reactions, each analyzed in triplicate). Most amino acids demonstrated CVs below 10%, meeting the established criteria for bioanalytical reproducibility [53, 54]. The only exception was cysteine exhibiting a higher variability, CV of 17%. This is attributed to the inherent instability of the thiol group and its probability of oxidation to cystine during sample processing (discussed in Sect. MS analysis of derivatized amino acids).

**Table 4:** Comparison of amino acid certified and non-certified concentrations in NIST plasma and measured concentration after derivatization with 6-CiQ-NHS

Amino acid	NIST values ref. (µM) [49]	NIST values measured (µM)	CV (%)
Ala	300.0 ± 26.0	357.3 ± 16.4	4.6
Arg^a^	81.4 ± 2.3	95.1 ± 6.7	7.1
Cys^a^	44.3 ± 6.9	19.3 ± 3.3	17.0
Gly	245.0 ± 16.0	871.4 ± 35.4	4.1
Ile	55.5 ± 3.4	65.3 ± 5.8	8.8
Leu	100.4 ± 6.3	122.1 ± 7.7	6.3
Lys	140.0 ± 14.0	248.5 ± 5.9	2.4
Met	22.3 ± 1.8	23.1 ± 1.5	6.5
Phe^>a^	51.0 ± 7.0	80.0 ± 7.9	9.9
Pro	177.0 ± 9.0	222.3 ± 8.6	3.9
Ser	95.9 ± 4.3	102.5 ± 4.8	4.7
Thr^a^	119.5 ± 6.1	158.3 ± 7.9	5.0
Tyr	57.3 ± 3.0	80.2 ± 4.0	4.9
Val	182.2 ± 10.4	234.8 ± 9.9	4.2

^a^Concentrations of these amino acids are not certified

## Conclusions

The systematic investigation of derivatization agent structures and functional groups revealed several key findings. A critical trade-off between reactivity and stability was evident. Agents with COCl and SO₂Cl groups were highly reactive but unstable in solution and during storage. In contrast, NHS ester-based agents were highly stable, retaining full activity for over 1 year, though they required longer reaction times. Positional isomerism of the functional group on the heterocyclic core significantly influenced synthetic feasibility, derivatization kinetics, chromatographic behavior, and MS response, establishing clear structure-property relationships. Among the agents studied, 6-CiQ-NHS emerged as the most promising, providing an optimal balance of stability, efficient derivatization, and strong analytical signal.

A dedicated LC-MS method was developed and validated using human plasma. The method demonstrated excellent linearity (*R*^2^ ≥ 0.9954), accuracy, and precision. Low nanomolar LODs (2.3–63.3 nM) were achieved using only 0.1 µL injection volumes, highlighting the sensitivity of the approach. Compared with the commercially available DNS agent, 6-CiQ-NHS provided a 4- to 10-fold improvement in sensitivity and enabled critical separation of isomeric amino acids isoleucine/leucine that co-eluted under the DNS method.

The applicability of the 6-CiQ-NHS method for accurate quantification in plasma was further confirmed through analysis of the NIST SRM 1950 reference material. Reproducibility was high, with coefficients of variation below 10% for most amino acids, except for cysteine, which is inherently unstable and prone to disulfide bond formation. Overall, the 6-CiQ-NHS derivatization agent and associated LC-MS method provide a highly sensitive and reliable approach for amino acid quantification in biological samples. The established structure–property relationships offer a valuable framework for the rational design and selection of future derivatization agents.

## Supplementary Information

Below is the link to the electronic supplementary material.

Supplementary file1 (DOCX 12.0 MB)

## Data Availability

The authors confirm that all primary data supporting the findings of this study are included within the article and its associated Supplementary Information files. Any additional raw data or specific datasets generated during the current study are available from the corresponding author upon reasonable request.

## Abbreviations

1-CiQ: Isoquinoline-1-carboxylic acid
1-CiQ-NHS: (2,5-Dioxopyrrolidin-1-yl) isoquinoline-1-carboxylate
2-CQ: Quinoline-2-carboxylic acid
2-CQ-NHS: (2,5-Dioxopyrrolidin-1-yl) quinoline-2-carboxylate
3-AP: Pyridine-3-amine
3-CiQ: Isoquinoline-3-carboxylic acid
3-CiQ-NHS: (2,5-Dioxopyrrolidin-1-yl) isoquinoline-3-carboxylate
3-CP-COCl: Pyridine-3-carbonyl chloride
3-CP: Pyridine-3-carboxylic acid
3-CP-NHS: (2,5-Dioxopyrrolidin-1-yl) pyridine-3-carboxylate
3-CQ: Quinoline-3-carboxylic acid
3-CQ-NHS: (2,5-Dioxopyrrolidin-1-yl) quinoline-3-carboxylate
3-SP-SO_2_Cl: Pyridine-3-sulfonyl chloride
3-SP: Pyridine-3-sulfonic acid
4-CQ: Quinoline-4-carboxylic acid
4-CQ-NHS: (2,5-Dioxopyrrolidin-1-yl) quinoline-4-carboxylate
5-AiQ: Isoquinoline-5-amine
5-CiQ-COCl: Isoquinoline-5-carbonyl chloride
5-CiQ: Isoquinoline-5-carboxylic acid
5-CiQ-NHS: (2,5-Dioxopyrrolidin-1-yl) isoquinoline-5-carboxylate
5-CQ: Quinoline-5-carboxylic acid
5-CQ-NHS: (2,5-Dioxopyrrolidin-1-yl) quinoline-5-carboxylate
5-SiQ: Isoquinoline-5-sulfonic acid
5-SiQ-SO_2_Cl: Isoquinoline-5-sulfonyl chloride
6-CiQ-COCl: Isoquinoline-6-carbonyl chloride
6-CiQ: Isoquinoline-6-carboxylic acid
6-CiQ-NHS: (2,5-Dioxopyrrolidin-1-yl) isoquinoline-6-carboxylate
6-CQ-COCl: Quinoline-6-carbonyl chloride
6-CQ: Quinoline-6-carboxylic acid
6-CQ-NHS: (2,5-Dioxopyrrolidin-1-yl) quinoline-6-carboxylate
7-CQ: Quinoline-7-carboxylic acid
7-CQ-NHS: (2,5-Dioxopyrrolidin-1-yl) quinoline-7-carboxylate
8-CQ: Quinoline-8-carboxylic acid
8-CQ-NHS: (2,5-Dioxopyrrolidin-1-yl) quinoline-8-carboxylate
AQC: 6-Aminoquinolyl-*N*-hydroxysuccinimidyl carbamate
AzoC: 4-(Phenylazo)benzoic acid N-succinimidyl ester
DNS: Dansyl chloride
DSC: *N*,*N′*-Disuccinimidyl carbonate
Em_(max)_: Emission maximum
ESI: Electrospray ionization
Ex_(max)_: Excitation maximum
FLD: Fluorescence detection
Fmoc-Cl: 9-Fluorenylmethoxycarbonyl chloride
Fmoc-OH: 9-Fluorenylmethanol
GABA: γ-Aminobutyric acid
LC-MS: Liquid chromatography-mass spectrometry
m.p.: Melting point
MRM: Multiple reaction monitoring
MS: Mass spectrometry
NHS ester: *N*-Hydroxysuccinimidyl ester reactive group
OPA: *o*-Phthalaldehyde
RP: Reversed-phase
UV: Ultra-violet detection

## Declarations

### AI-assisted copy editing

During the preparation of this work, the authors used Grammarly and Gemini as grammar correction tools in order to improve the language aspects of the manuscript. After using these tools, the authors reviewed and edited the content as needed and take full responsibility for the content of the published article.
