# Supplementary material for: Derivatization agents for LC-MS analysis of amino acids: effects of core structure and functional groups
Source: Anal Bioanal Chem. 2026 Feb 10;418(8):2373–91. doi: 10.1007/s00216-026-06366-9 (PMC13065604; doi:10.1007/s00216-026-06366-9)
Supplement: Supplementary file 1 — Supplementary file1 (DOCX 12.0 MB) [file 216_2026_6366_MOESM1_ESM.docx]

**Supporting information**

**Derivatization agents for LC-MS analysis of amino acids: Effects of core structure and functional groups**

Tereza Hofmanova^a^, Rudolf Andrys ^a^*, Miroslav Lisa^a^

*^a^Department of Chemistry, Faculty of Science, University of Hradec Kralove, Rokitanskeho 62, 50003 Hradec Kralove, Czech Republic*

**Table of contents**

[1 Development and characterization of Pyridine, Isoquinoline, and Quinoline-Based Derivatization Agents 2](#_Toc214537310)

[1.1 Preparation of derivatization agents 2](#_Toc214537311)

[1.2 NMR characterization of final products 2](#_Toc214537312)

[1.2.1 NMR characterization of final products 2](#_Toc214537313)

[1.2.2 NMR spectra of agents 7](#_Toc214537314)

[1.3 Synthetic reaction scheme 25](#_Toc214537315)

[2 Preliminary methods 25](#_Toc214537316)

[2.1 Analysis of derivatized amino acid standards 25](#_Toc214537317)

[2.2 Analysis of DNS derivatized amino acids standards 26](#_Toc214537318)

[3 Solvent stability of derivatization agents 27](#_Toc214537319)

[4 Absorbance and fluorescence possibilities 29](#_Toc214537320)

[5 MRM fragmentation and MS behavior of derivatized amino acids 29](#_Toc214537321)

[6 Derivatization kinetic profiles 34](#_Toc214537322)

[7 Chromatographic retention behavior of derivatives 34](#_Toc214537323)

[8 pH influence on derivatization 35](#_Toc214537324)

[9 Effects of the temperature on the derivatization reaction 35](#_Toc214537325)

[10 Stability of derivatized products 36](#_Toc214537326)

[11 Linearity in presence and absence of matrix for 6-CiQ-NHS derivatization 40](#_Toc214537327)

[12 Calibration curves for amino acids quantification 41](#_Toc214537328)

# Development and characterization of Pyridine, Isoquinoline, and Quinoline-Based Derivatization Agents

## Preparation of derivatization agents

**Table S1**: Summary of obtained yields from synthesis of derivatization agents.

| **Functional group** | **Compound** | **Yield (%)** |
| --- | --- | --- |
| NCOO | 3-AP_NCOO | 0 |
|  | 5-AiQ_NCOO | 0 |
| SO_2_Cl | 3-SP-SO_2_Cl | 21 |
|  | 5-SiQ-SO_2_Cl | 74 |
| COCl | 3-CP-COCl | quantitative |
|  | 5-CiQ-COCl | quantitative |
|  | 6-CiQ-COCl | quantitative |
|  | 6-CQ-COCl | quantitative |
| NHS | 3-CP-NHS | 82 |
|  | 1-CiQ-NHS | 30 |
|  | 3-CiQ-NHS | 30 |
|  | 5-CiQ-NHS | 75 |
|  | 6-CiQ-NHS | 50 |
|  | 2-CQ-NHS | 62 |
|  | 3-CQ-NHS | 69 |
|  | 4-CQ-NHS | 67 |
|  | 5-CQ-NHS | 50 |
|  | 6-CQ-NHS | 77 |
|  | 7-CQ-NHS | 99 |
|  | 8-CQ-NHS | 65 |

## NMR characterization of final products

The ^1^H and ^13^C NMR spectra were measured in DMSO-*d_6_* solution and CDCl_3_ solution at room temperature on FT NMR spectrometer Avance NEO 500 MHz (499.87 MHz for ^1^H and 125.71 MHz for ^13^C) (Bruker, Germany). Chemical shifts (*δ*) are given in parts per million (ppm) and spin multiplicities are given as s (singlet), d (doublet), t (triplet) or m (multiplet). Coupling constants, J, are expressed in hertz (Hz). For ^1^H *δ* is relative to DMSO-*d_6_* (*δ* = 2.50), to CDCl_3_ (*δ* = 7.26) and for ^13^C is relative to DMSO-*d_6_* (*δ* = 39.43) and to CDCl_3_ (*δ* = 77.16).

### NMR characterization of final products

#### SO_2_Cl derivatization agents

*Pyridine-3-sulfonyl chloride* (**3-SP-SO_2_Cl**) (**Figure S1**). Dark red amorphous substance; Yield 35 mg (21%); ^1^H NMR (500 MHz, DMSO-*d_6_*): δ 8.99 (d; J = 1.8 Hz; 1H; ArH), 8.90 (d; J = 5.6 Hz; 1H; ArH), 8.71 (dt; J = 8.0, 1.6 Hz; 1H; ArH), 8.08 (dd; J = 8.0, 5.7 Hz; 1H; ArH); ^13^C NMR (126 MHz, DMSO-*d_6_*): δ 146.83, 142.83, 142.36, 138.91, 127.44. MS (ESI^+^): [M+H]^+^: calculated for C_5_H_5_ClNO_2_S^+^ (m/z): 177.97; found: 177.90.

*Isoquinoline-5-sulfonyl chloride* (**5-SiQ-SO_2_Cl**) (**Figure S2**). Purple amorphous substance; Yield 121 mg (74%); ^1^H NMR (500 MHz, DMSO-*d_6_*): δ 9.95 (s; 1H; ArH), 9.16 (d; J = 6.8 Hz; 1H; ArH), 8.74 (d; J = 6.8 Hz; 1H; ArH), 8.51 (dd; J = 13.5, 7.7 Hz; 2H; ArH), 7.98 (t; J = 7.7 Hz; 1H; ArH); ^13^C NMR (126 MHz, DMSO-*d_6_*): δ 147.16, 143.95, 134.78, 133.44, 131.71, 131.46, 129.82, 127.72, 124.47. MS (ESI^+^): [M+H]^+^: calculated for C_9_H_7_ClNO_2_S^+^ (m/z): 227.98; found: 228.10.

#### COCl derivatization agents

*Pyridine-3-carbonyl chloride* (**3-CP-COCl**) (**Figure S3**). White solid; Yield quantitative; m.p. 150.5-152.0°C; ^1^H NMR (500 MHz, DMSO-*d_6_*): δ 9.20 (d; J = 1.8 Hz; 1H; ArH), 9.06 (dd; J = 5.6, 1.3 Hz; 1H; ArH), 8.83 (dt; J = 8.1, 1.7 Hz; 1H; ArH), 8.07 (dd; J = 7.9, 5.7 Hz; 1H; ArH); ^13^C NMR (126 MHz, DMSO-*d_6_*): δ 163.89, 146.29, 144.36, 143.95, 129.63, 126.97. MS (ESI^+^): [M+H]^+^: calculated for C_6_H_5_ClO^+^ (m/z): 142.00, 144.00; found: 141.90, 143.90.

*Isoquinoline-5-carbonyl chloride* (**5-CiQ-COCl**) (**Figure S4**). Beige solid; Yield quantitative; m.p. 166.8-168.5°C; ^1^H NMR (500 MHz, DMSO-*d_6_*): δ 10.07 (s; 1H; ArH), 9.30 (d; J = 6.9 Hz; 1H; ArH), 8.82-8.76 (m; 3H; ArH), 8.10 (dd; J = 8.2, 7.5 Hz; 1H; ArH); ^13^C NMR (126 MHz, DMSO-*d_6_*): δ 166.70, 147.72, 139.49, 136.51, 135.61, 132.97, 129.91, 127.92, 127.05, 123.03. MS (ESI^+^): [M+H]^+^: calculated for C_10_H_7_ClO^+^ (m/z): 192.02, 194.01; found: 192.00, 194.00.

*Isoquinoline-6-carbonyl chloride* (**6-CiQ-COCl**) (**Figure S5**). Light brown solid; Yield quantitative; m.p. 216.5-218.2°C; ^1^H NMR (500 MHz, DMSO-*d_6_*): δ 10.06 (s; 1H; ArH), 8.94 (s; 1H; ArH), 8.79 (d; J = 6.4 Hz; 1H; ArH), 8.73 (d; J = 6.4 Hz; 1H; ArH), 8.65 (d; J = 8.6 Hz; 1H; ArH), 8.39 (dd; J = 8.6, 1.4 Hz; 1H; ArH); ^13^C NMR (126 MHz, DMSO-*d_6_*): δ 166.08, 147.36, 138.04, 136.87, 132.41, 131.13, 129.41, 129.24, 128.80, 126.43. MS (ESI^+^): [M+H]^+^: calculated for C_10_H_7_ClO^+^ (m/z): 192.02, 194.01; found: 192.00, 194.00.

*Quinoline-6-carbonyl chloride* (**6-CQ-COCl**) (**Figure S6**). Brown solid; Yield quantitative; m.p. 193.2-195.0°C; ^1^H NMR (500 MHz, DMSO-*d_6_*): δ 9.39 (dd; J = 5.2, 1.3 Hz; 1H; ArH), 9.34 (d; J = 8.1 Hz; 1H; ArH), 8.99 (s; 1H; ArH), 8.58-8.50 (m; 2H; ArH), 8.15 (dd; J = 8.3, 5.2 Hz; 1H; ArH); ^13^C NMR (126 MHz, DMSO-*d_6_*): δ 166.01, 147.31, 146.91, 140.26, 133.11, 131.45, 131.15, 128.16, 122.94, 122.18. MS (ESI^+^): [M+H]^+^: calculated for C_10_H_7_ClO^+^ (m/z): 192.02, 194.01; found: 191.90, 193.90.

#### NHS ester derivatization agents

*(2,5-dioxopyrrolidin-1-yl) pyridine-3-carboxylate* (**3-CP-NHS**) (**Figure S7**). White solid; Yield 82 mg (82%); m.p. 121.7-123.7°C; ^1^H NMR (500 MHz, CDCl_3_): δ 9.34 (s; 1H; ArH), 8.91 (d; *J* = 3.5 Hz; 1H; ArH), 8.42 (d; *J* = 8.0 Hz; 1H; ArH), 7.51 (dd; *J* = 7.7, 5.0 Hz; 1H; ArH), 2.93 (s; 4H; 2 × CH_2_); ^13^C NMR (126 MHz, CDCl_3_): δ 169.01, 160.88, 155.11, 151.40, 138.11, 123.86, 121.89, 25.78. MS (ESI^+^): [M+H]^+^: calculated for C_10_H_9_N_2_O_4_^+^ (m/z): 221.05; found: 221.10. Ability of fluorescence at Ex_(max)_ = 324 nm and Em_(max)_ = 382 nm.

*(2,5-dioxopyrrolidin-1-yl) isoquinoline-1-carboxylate* (**1-CiQ-NHS**) (**Figure S8**). Beige solid; Yield 23 mg (30%); m.p. 131.5-133.2°C; ^1^H NMR (500 MHz, CDCl_3_): δ 8.84 (d; *J* = 8.7 Hz; 1H; ArH), 8.74 (d; *J* = 5.5 Hz; 1H; ArH), 7.97-7.93 (m; 2H; ArH), 7.79 (ddd; *J* = 8.2, 6.9, 1.3 Hz; 1H; ArH), 7.75 (ddd; *J* = 8.2, 6.9, 1.3 Hz; 1H; ArH), 2.91 (s; 4H; 2 × CH_2_); ^13^C NMR (126 MHz, CDCl_3_): δ 169.11, 160.68, 143.63, 142.04, 137.05, 131.23, 129.94, 127.88, 127.56, 126.14, 125.80, 25.92. MS (ESI^+^): [M+H]^+^: calculated for C_14_H_11_N_2_O_4_^+^ (m/z): 271.07; found: 271.10. Ability of fluorescence at Ex_(max)_ = 343 nm and Em_(max)_ = 413 nm.

*(2,5-dioxopyrrolidin-1-yl) isoquinoline-3-carboxylate* (**3-CiQ-NHS**) (**Figure S9**). White solid; Yield 23 mg (30%); m.p. 168.6-170.1°C; ^1^H NMR (500 MHz, CDCl_3_): δ 9.41 (s; 1H; ArH), 8.72 (s; 1H; ArH), 8.13 (d; *J* = 5.3 Hz; 1H; ArH), 8.02 (d; *J* = 6.0 Hz; 1H; ArH), 7.86 (s br; 2H; ArH), 2.95 (s; 4H; 2 × CH_2_); ^13^C NMR (126 MHz, CDCl_3_): δ 169.11, 160.91, 153.37, 137.11, 135.24, 132.12, 131.05, 130.54, 128.45, 128.21, 126.69, 25.88. MS (ESI^+^): [M+H]^+^: calculated for C_14_H_11_N_2_O_4_^+^ (m/z): 271.07; found: 271.10. Ability of fluorescence at Ex_(max)_ = 302 nm and Em_(max)_ = 362 nm.

*(2,5-dioxopyrrolidin-1-yl) isoquinoline-5-carboxylate* (**5-CiQ-NHS**) (**Figure S10**). Beige solid; Yield 58 mg (75%); m.p. 179.3-181.1°C; ^1^H NMR (500 MHz, CDCl_3_): δ 9.40 (s; 1H; ArH), 8.72 (s; 1H; ArH), 8.67 (d; *J* = 4.8 Hz; 1H; ArH), 8.22 (dd; *J* = 8.5, 1.1 Hz; 1H; ArH), 8.12 (d; *J* = 8.6 Hz; 1H; ArH), 7.81 (d; *J* = 5.6 Hz; 1H; ArH), 2.95 (s; 4H; 2 × CH_2_); ^13^C NMR (126 MHz, CDCl_3_): δ 169.15, 161.49, 152.50, 144.00, 135.02, 131.32, 130.54, 128.87, 126.96, 126.90, 121.76, 25.84. MS (ESI^+^): [M+H]^+^: calculated for C_14_H_11_N_2_O_4_^+^ (m/z): 271.07; found: 271.20. Ability of fluorescence at Ex_(max)_ = 330 nm and Em_(max)_ = 371 nm.

*(2,5-dioxopyrrolidin-1-yl) isoquinoline-6-carboxylate* (**6-CiQ-NHS**) (**Figure S11**). Light beige soli; Yield 39 mg (50%); m.p. 181.3-182.9°C; ^1^H NMR (500 MHz, CDCl_3_): δ 9.41 (s; 1H; ArH), 8.69 (d; *J* = 7.4 Hz; 3H; ArH), 8.33 (d; *J* = 8.1 Hz; 1H; ArH), 7.74 (t; *J* = 7.7 Hz; 1H; ArH), 2.97 (s; 4H; 2 × CH_2_); ^13^C NMR (126 MHz, CDCl_3_): δ 169.34, 161.20, 152.89, 145.13, 136.41, 135.59, 134.71, 128.89, 126.52, 120.89, 118.49, 25.88. MS (ESI^+^): [M+H]^+^: calculated for C_14_H_11_N_2_O_4_^+^ (m/z): 271.07; found: 271.20. Ability of fluorescence at Ex_(max)_ = 330 nm and Em_(max)_ = 386 nm.

*(2,5-dioxopyrrolidin-1-yl) quinoline-2-carboxylate* (**2-CQ-NHS**) (**Figure S12**). Light yellow solid; Yield 48 mg (62%); m.p. 118.6-120.5°C; ^1^H NMR (500 MHz, CDCl_3_): δ 8.36 (d; *J* = 8.6 Hz; 1H; ArH), 8.32 (d; *J* = 8.5 Hz; 1H; ArH), 8.21 (d; *J* = 8.5 Hz; 1H; ArH), 7.91 (d; *J* = 8.2 Hz; 1H; ArH), 7.83 (ddd; *J* = 8.5, 6.9, 1.4 Hz; 1H; ArH), 7.71 (ddd; *J* = 8.1, 6.9, 1.1 Hz; 1H; ArH), 2.95 (s; 4H; 2 × CH_2_); ^13^C NMR (126 MHz, CDCl_3_): δ 169.01, 160.83, 147.85, 143.92, 137.77, 131.05, 130.99, 129.95, 129.78, 127.79, 121.68, 25.86. MS (ESI^+^): [M+H]^+^: calculated for C_14_H_11_N_2_O_4_^+^ (m/z): 271.07; found: 270.90. Ability of fluorescence at Ex_(max)_ = 333 nm and Em_(max)_ = 388 nm.

*(2,5-dioxopyrrolidin-1-yl) quinoline-3-carboxylate* (**3-CQ-NHS**) (**Figure S13**). White solid; Yield 54 mg (69%); m.p. 176.4-177.9°C; ^1^H NMR (500 MHz, CDCl_3_): δ 9.47 (d; *J* = 2.2 Hz; 1H; ArH), 9.01 (d; *J* = 1.9 Hz; 1H; ArH), 8.22 (d; *J* = 8.5 Hz; 1H; ArH), 7.97 (d; *J* = 8.2 Hz; 1H; ArH), 7.92 (ddd; *J* = 8.4, 7.0, 1.4 Hz; 1H; ArH), 7.72-7.66 (m; 1H; ArH), 2.95 (s; 4H; 2 × CH_2_); ^13^C NMR (126 MHz, CDCl_3_): δ 169.13, 160.94, 150.21, 149.32, 140.80, 133.47, 129.54, 128.36, 126.60, 118.49, 25.83. MS (ESI^+^): [M+H]^+^: calculated for C_14_H_11_N_2_O_4_^+^ (m/z): 271.07; found: 271.10. Ability of fluorescence at Ex_(max)_ = 324 nm and Em_(max)_ = 385 nm.

*(2,5-dioxopyrrolidin-1-yl) quinoline-4-carboxylate* (**4-CQ-NHS**) (**Figure S14**). White solid; Yield 52 mg (67%); m.p. 121.3-123.4°C; ^1^H NMR (500 MHz, CDCl_3_): δ 9.10 (d; *J* = 4.5 Hz; 1H; ArH), 8.67-8.63 (m; 1H; ArH), 8.26 (d; *J* = 8.4 Hz; 1H; ArH), 8.12 (d; *J* = 4.4 Hz; 1H; ArH), 7.84 (ddd; *J* = 8.4, 6.9, 1.3 Hz; 1H; ArH), 7.73 (ddd; *J* = 8.3, 6.9, 1.2 Hz; 1H; ArH), 2.96 (s; 4H; 2 × CH_2_); ^13^C NMR (126 MHz, CDCl_3_): δ 169.00, 161.40, 149.33, 148.58, 130.83, 130.56, 130.00, 129.45, 125.19, 124.74, 123.00, 25.86. MS (ESI^+^): [M+H]^+^: calculated for C_14_H_11_N_2_O_4_^+^ (m/z): 271.07; found: 270.90. Ability of fluorescence at Ex_(max)_ = 333 nm and Em_(max)_ = 429 nm.

*(2,5-dioxopyrrolidin-1-yl) quinoline-5-carboxylate* (**5-CQ-NHS**) (**Figure S15**). White solid; Yield 39 mg (50%); m.p. 148.2-150.1°C; ^1^H NMR (500 MHz, CDCl_3_): δ 9.21-9.17 (m; 1H; ArH), 9.00 (dd; *J* = 4.1, 1.3 Hz; 1H; ArH), 8.52 (dd; *J* = 7.4, 1.1 Hz; 1H; ArH), 8.45 (d; *J* = 8.5 Hz; 1H; ArH), 7.82 (dd; *J* = 8.4, 7.5 Hz; 1H; ArH), 7.58 (dd; *J* = 8.8, 4.2 Hz; 1H; ArH), 2.95 (s; 4H; 2 × CH_2_); ^13^C NMR (126 MHz, CDCl_3_): δ 169.37, 161.42, 130.98, 147.78, 136.86, 134.29, 132.56, 128.47, 127.38, 123.36, 121.82, 25.86. MS (ESI^+^): [M+H]^+^: calculated for C_14_H_11_N_2_O_4_^+^ (m/z): 271.07; found: 271.00. Ability of fluorescence at Ex_(max)_ = 315 nm and Em_(max)_ = 375 nm.

*(2,5-dioxopyrrolidin-1-yl) quinoline-6-carboxylate* (**6-CQ-NHS**) (**Figure S16**). Light brown solid; Yield 60 mg (77%); m.p. 183.8-185.5°C; ^1^H NMR (500 MHz, CDCl_3_): δ 9.18 (dd; *J* = 4.7, 1.5 Hz; 1H; ArH), 8.88 (d; *J* = 1.7 Hz; 1H; ArH); 8.68 (dd; *J* = 8.2, 5.1 Hz; 2H; ArH), 8.50 (dd; *J* = 9.0, 1.8 Hz; 1H; ArH), 7.82 (dd; *J* = 8.4, 4.8 Hz; 1H; ArH), 2.96 (s; 4H; 2 × CH_2_). ^13^C NMR (126 MHz, CDCl_3_): δ 169.27, 161.58, 153.44, 150.46, 137.97, 132.97, 130.49, 129.16, 127.50, 123.34, 122.54, 25.84. MS (ESI^+^): [M+H]^+^: calculated for C_14_H_11_N_2_O_4_^+^ (m/z): 271.07; found: 271.10. Ability of fluorescence at Ex_(max)_ = 357 nm and Em_(max)_ = 466 nm.

*(2,5-dioxopyrrolidin-1-yl) quinoline-7-carboxylate* (**7-CQ-NHS**) (**Figure S17**). White solid; Yield 78 mg (99%); m.p. 135.5-137.5°C;^1^H NMR (500 MHz, CDCl_3_): δ 9.05 (dd; *J* = 4.2, 1.5 Hz; 1H; ArH), 9.00 (s; 1H; ArH), 8.28 (d; *J* = 8.2 Hz; 1H; ArH), 8.17 (dd; *J* = 8.5, 1.7 Hz; 1H; ArH), 7.96 (d; *J* = 8.6 Hz; 1H; ArH), 7.59 (dd; *J* = 8.3, 4.2 Hz; 1H; ArH), 2.94 (s; 4H; 2 × CH_2_); ^13^C NMR (126 MHz, CDCl_3_): δ 169.21, 161.70, 151.60, 146.62, 136.74, 133.47, 131.79, 129.00, 126.31, 126.24, 123.88, 25.84. MS (ESI^+^): [M+H]^+^: calculated for C_14_H_11_N_2_O_4_^+^ (m/z): 271.07; found: 270.90. Ability of fluorescence at Ex_(max)_ = 320 nm and Em_(max)_ = 381 nm.

*(2,5-dioxopyrrolidin-1-yl) quinoline-8-carboxylate* (**8-CQ-NHS**) (**Figure S18**). White solid; Yield 51 mg (65%); m.p. 145.6-147.5°C; ^1^H NMR (500 MHz, CDCl_3_): δ 9.25 (dd; *J* = 4.3, 1.6 Hz; 1H; ArH), 8.52 (dd; *J* = 7.3, 1.3 Hz; 1H; ArH), 8.35 (dd; *J* = 8.3, 1.5 Hz; 1H; ArH), 8.16 (dd; *J* = 8.2, 1.1 Hz; 1H; ArH), 7.71-7.67 (m; 1H; ArH), 7.62 (d; *J* = 4.4 Hz; 1H; ArH), 2.96 (s; 4H; 2 × CH_2_); ^13^C NMR (126 MHz, CDCl_3_): δ 169.56, 167.24, 161.23, 152.28, 138.20, 134.66, 134.24, 128.79, 126.11, 122.55, 25.92. MS (ESI^+^): [M+H]^+^: calculated for C_14_H_11_N_2_O_4_^+^ (m/z): 271.07; found: 271.00.

### NMR spectra of agents




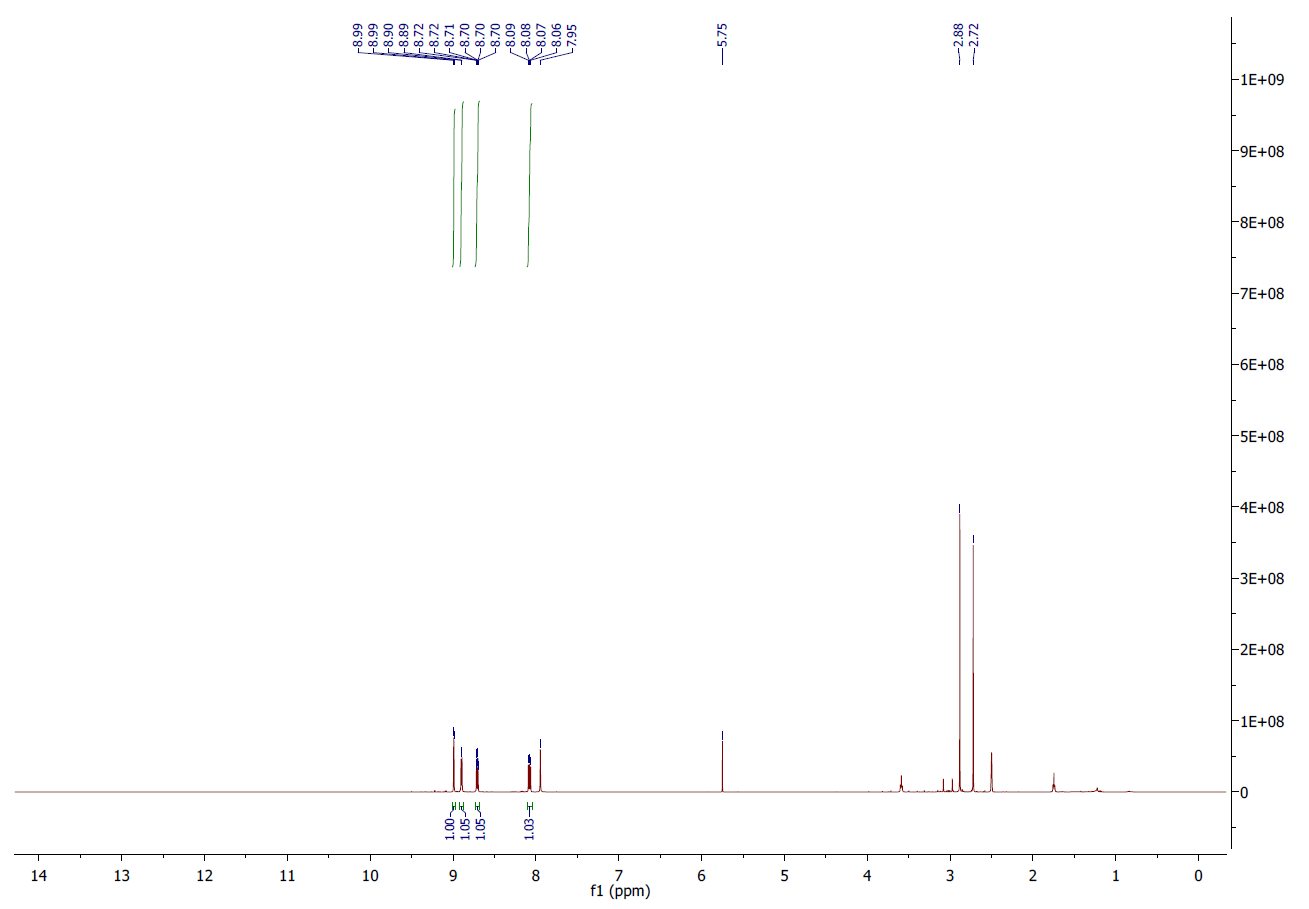


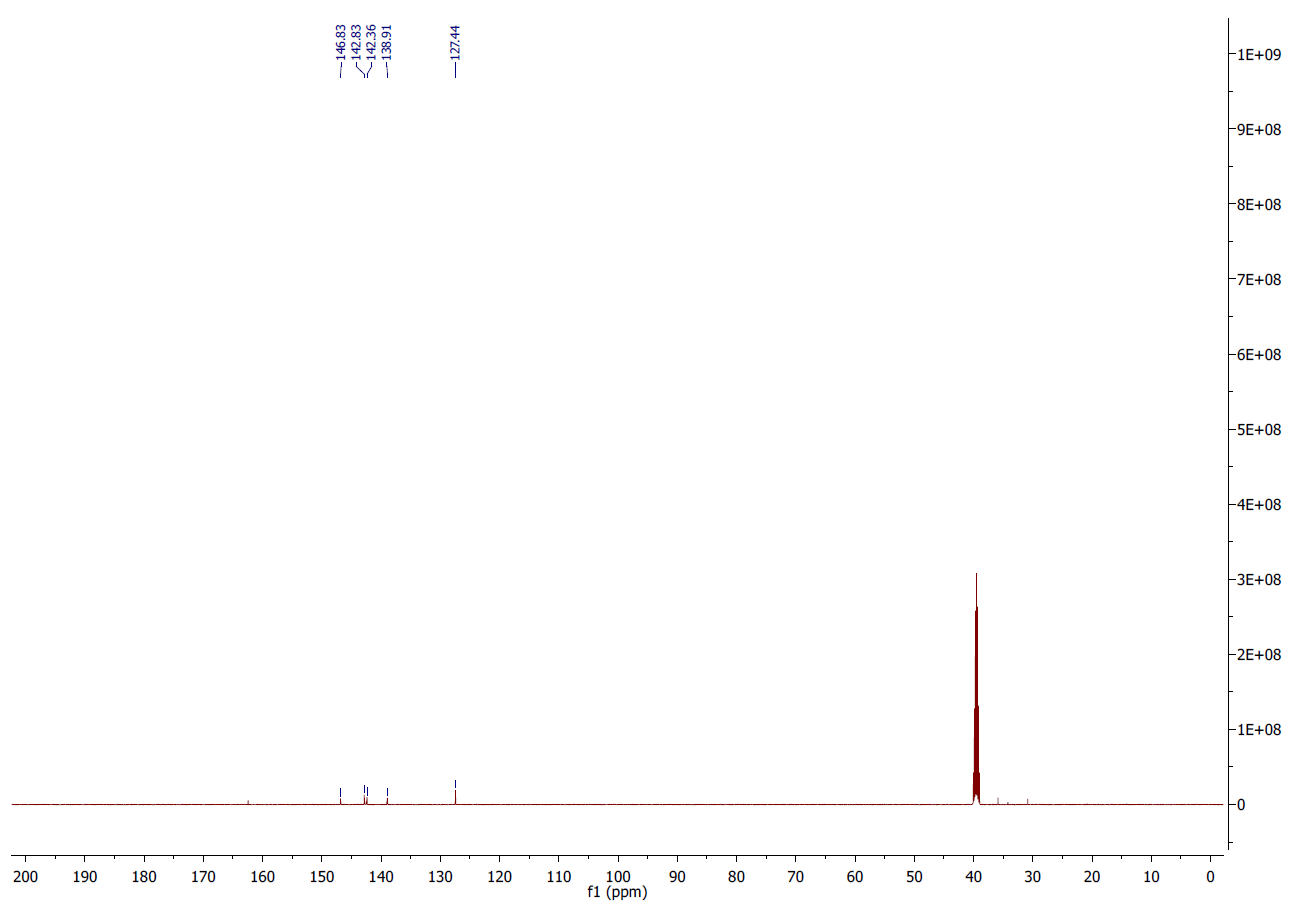


**Figure S1:** ^1^H (**A**) and ^13^C (**B**) NMR spectra of 3-SP-SO_2_Cl in DMSO-*d_6_* (Due to product instability during solvent evaporation, residual peaks for DCM (^1^H: 5.75) and DMF (^1^H: 2.72, 2.88, 7.95; ^13^C: 30.86, 35.88, 162.42) are present in both NMR spectra).




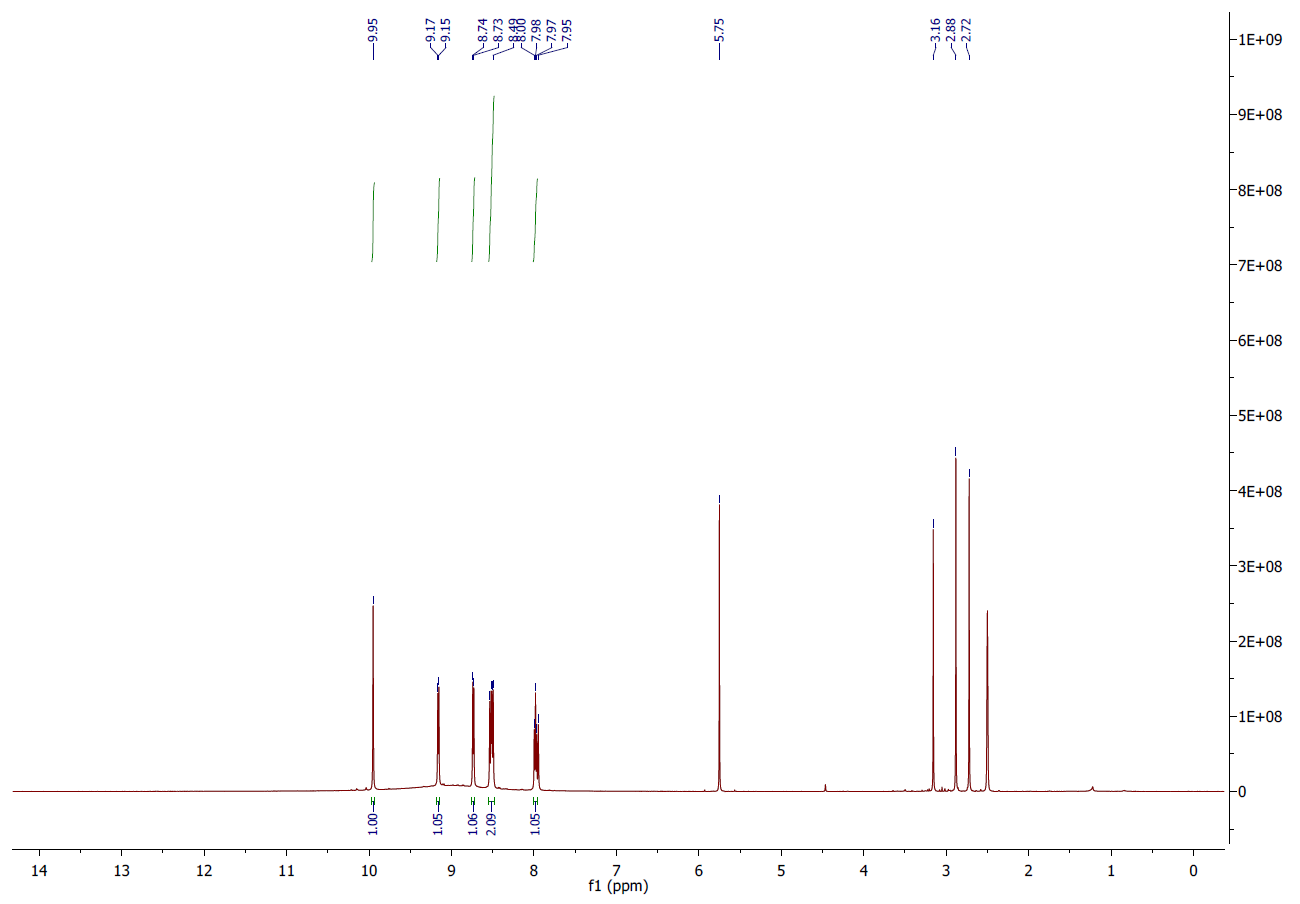


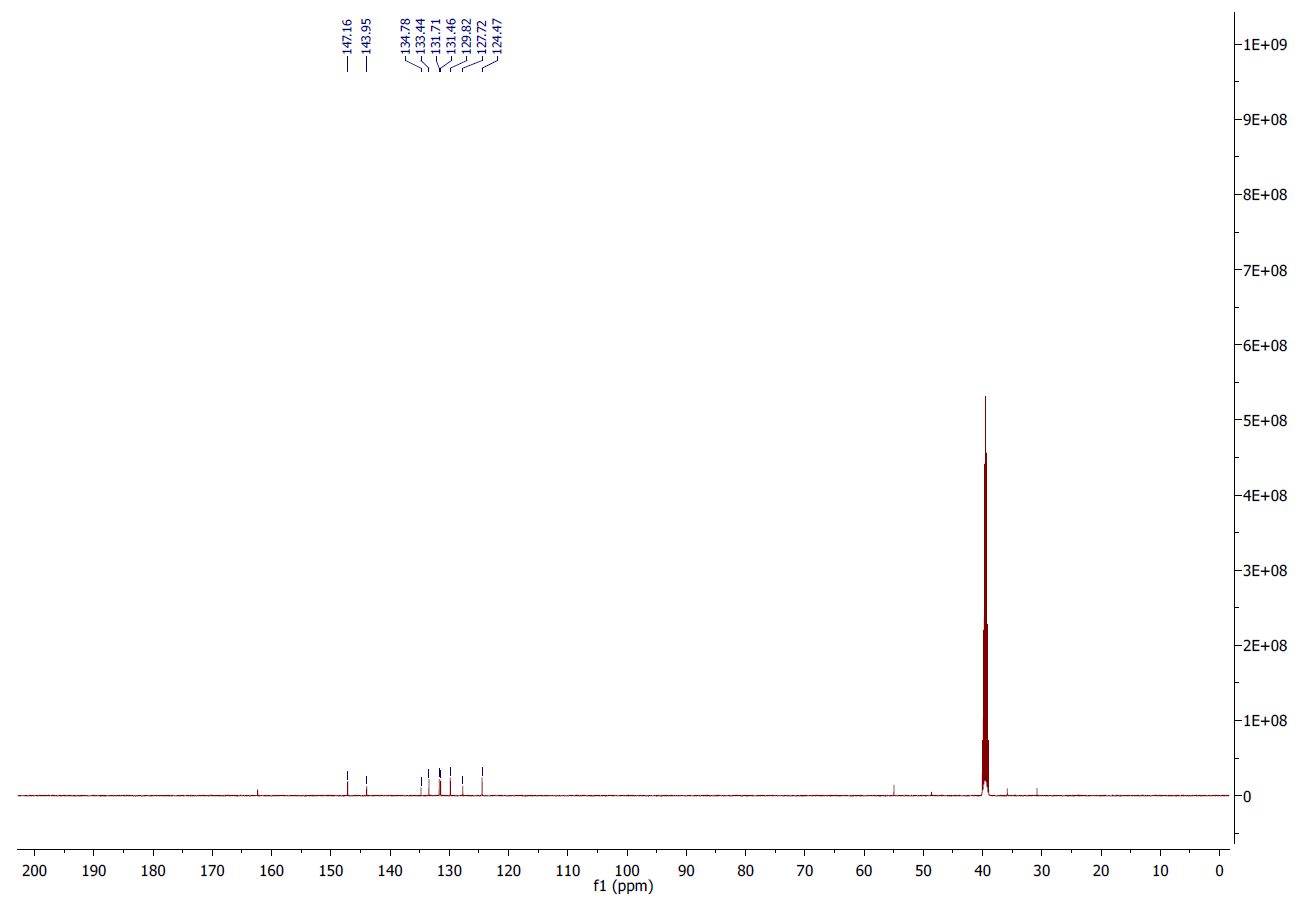


**Figure S2**: ^1^H (**A**) and ^13^C (**B**) NMR spectra of 5-SiQ-SO_2_Cl in DMSO-*d_6_* (Due to product instability during solvent evaporation, residual peaks for DCM (^1^H: 5.75; ^13^C: 54.96), MeOH (^1^H: 3.16; ^13^C: 48.61), and DMF (^1^H: 2.72, 2.88, 7.95; ^13^C: 30.82, 35.83, 162.36) are present in both NMR spectra).




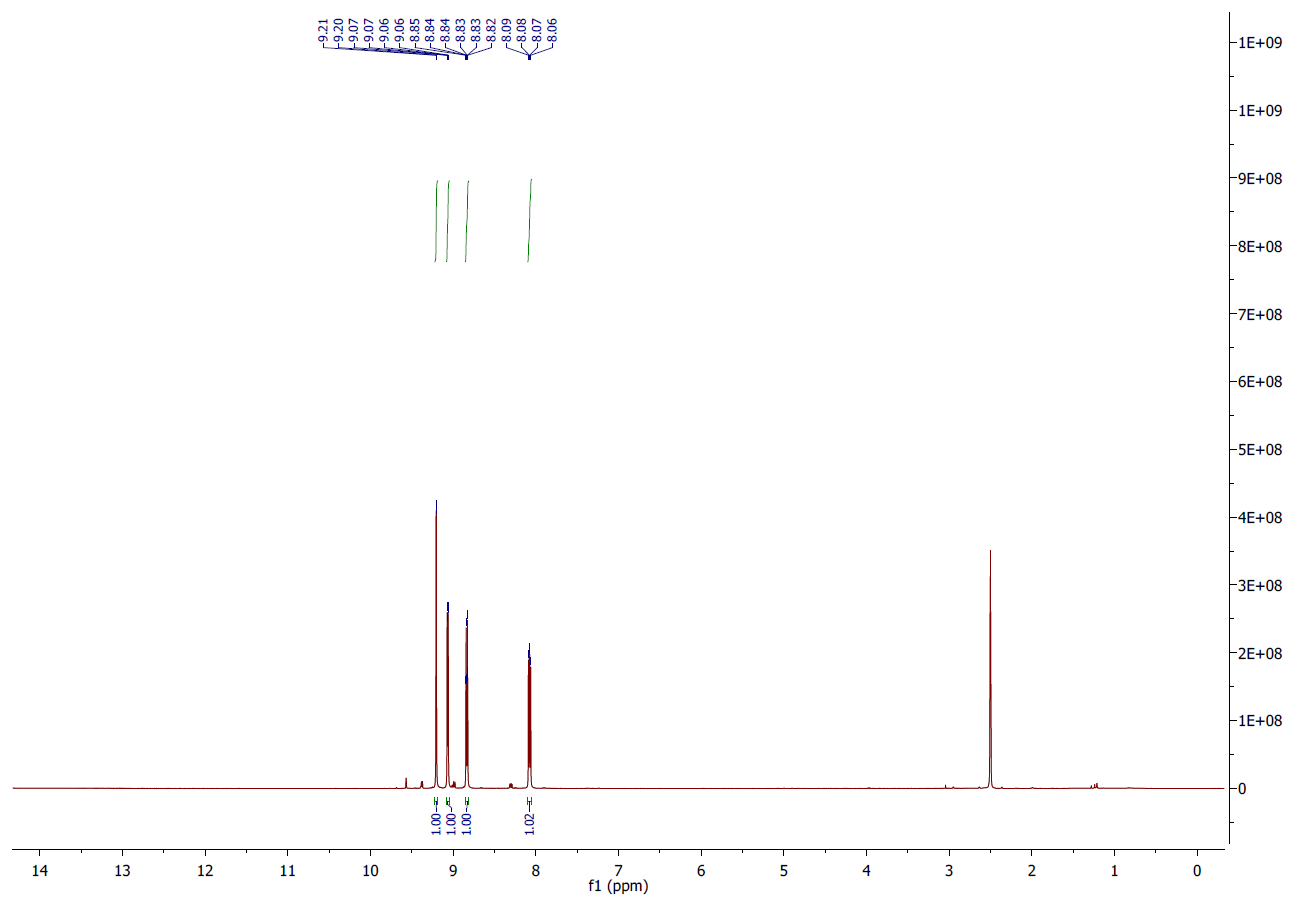


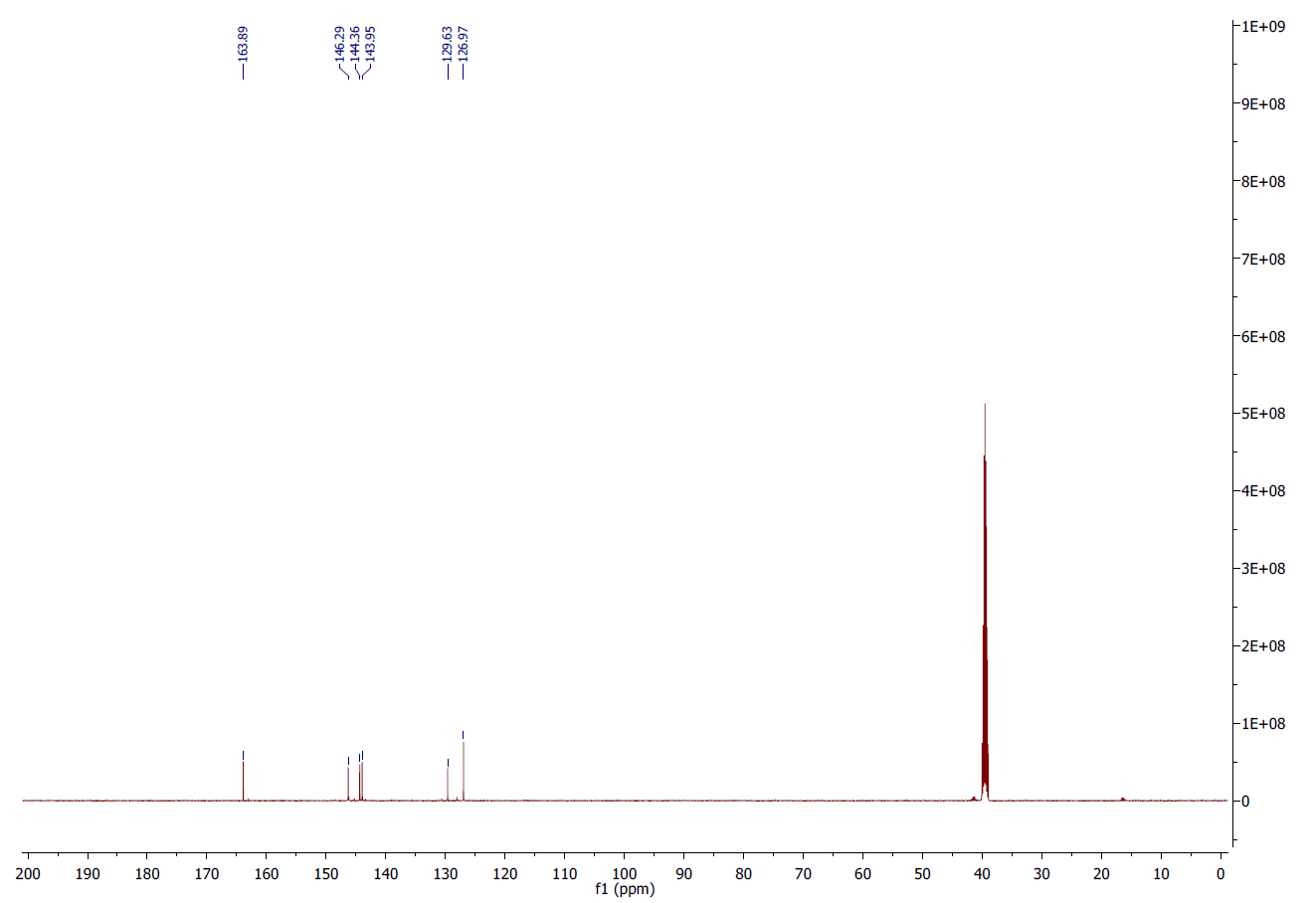


**Figure S3**: ^1^H (**A**) and ^13^C (**B**) NMR spectra of 3-CP-COCl in DMSO-*d_6_*.




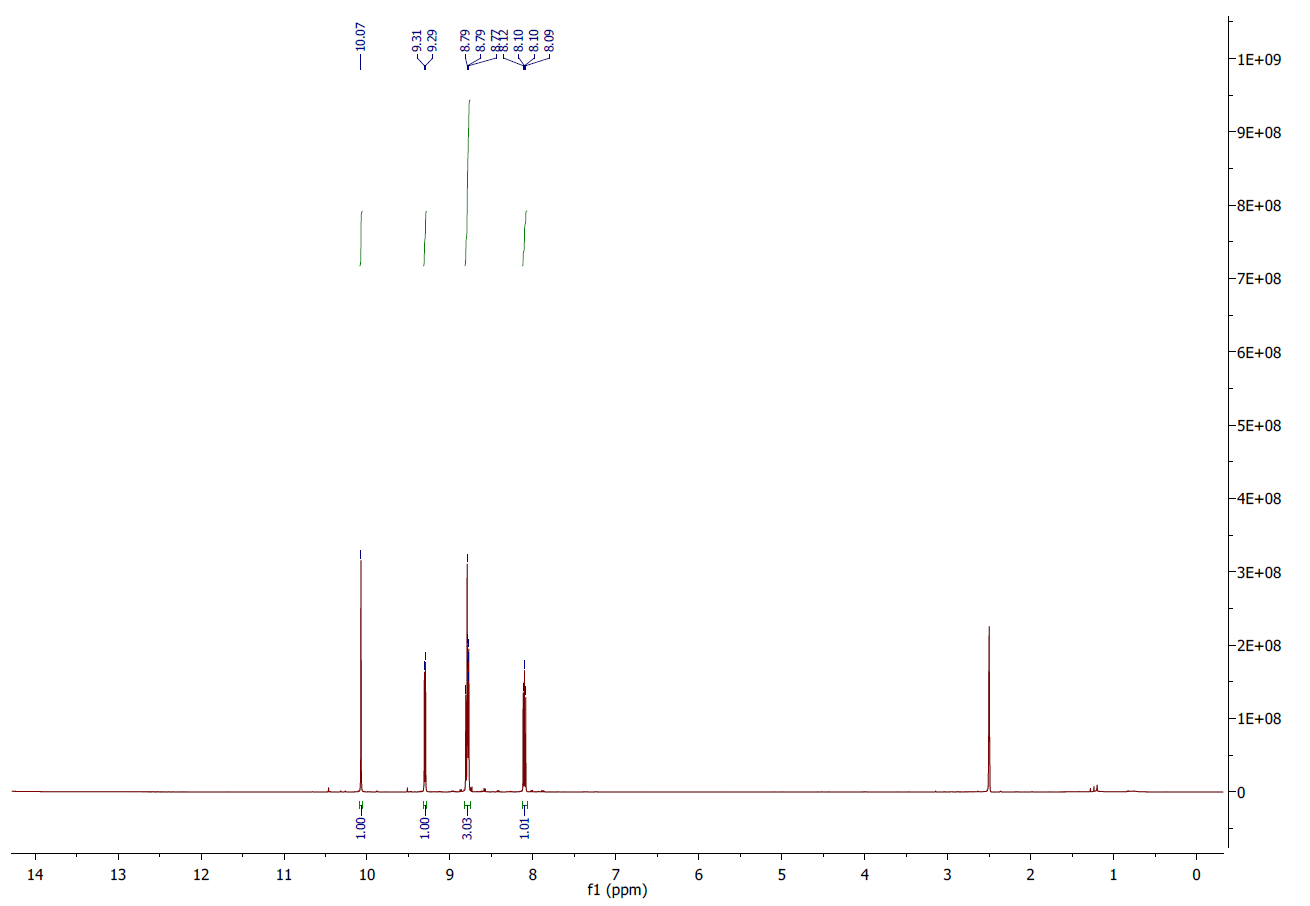


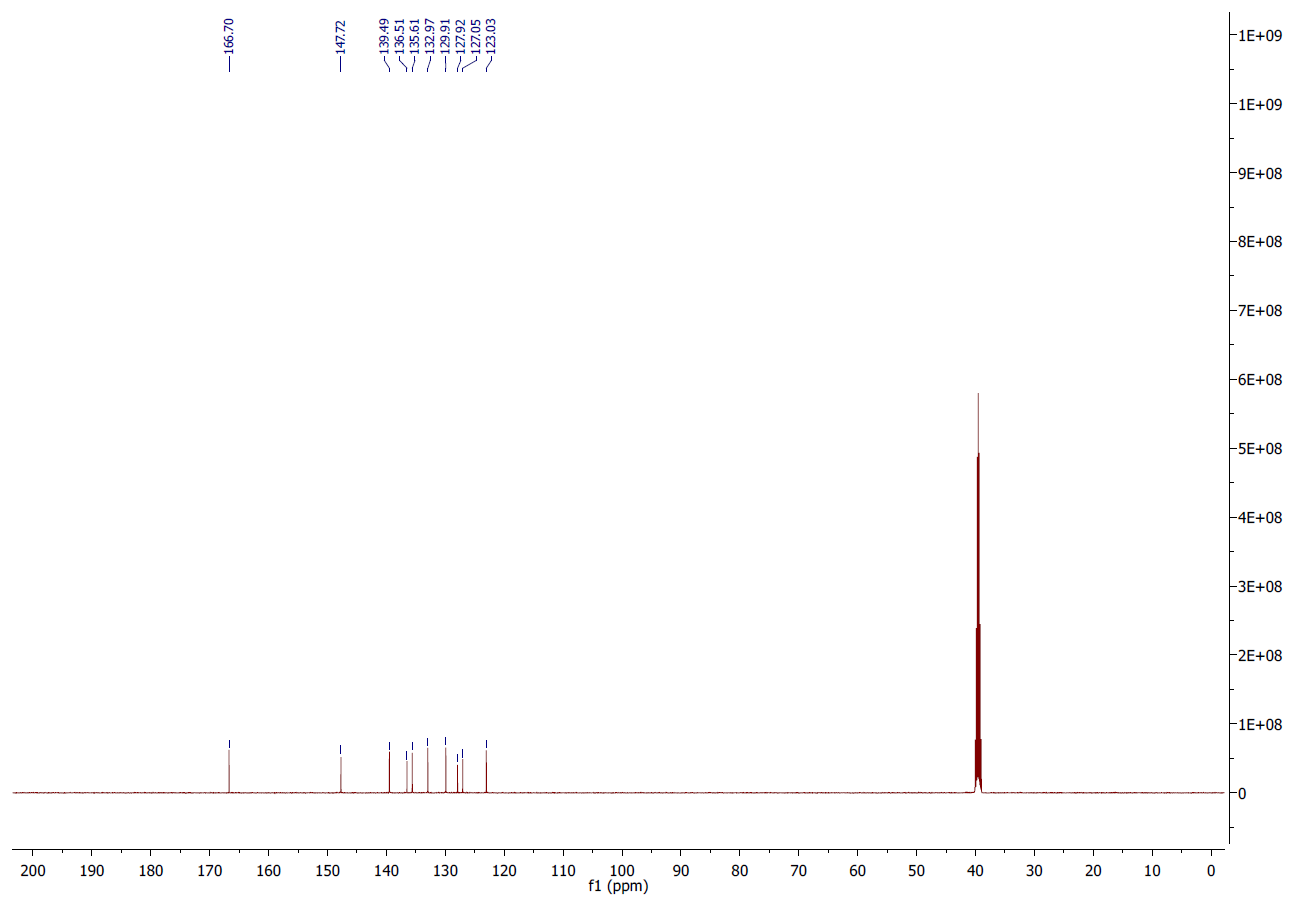


**Figure S4**: ^1^H (**A**) and ^13^C (**B**) NMR spectra of 5-CiQ-COCl in DMSO-*d_6_*.




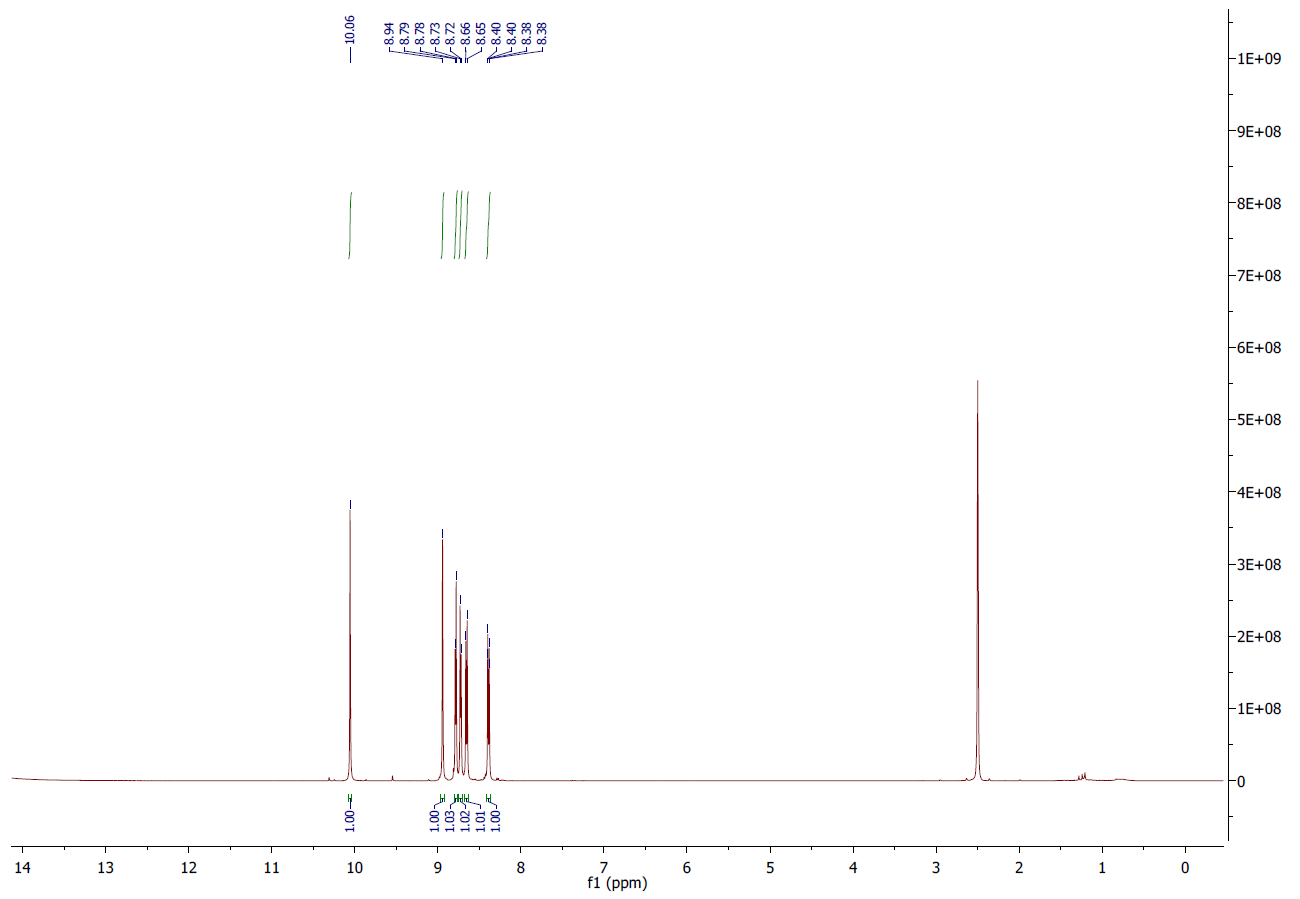


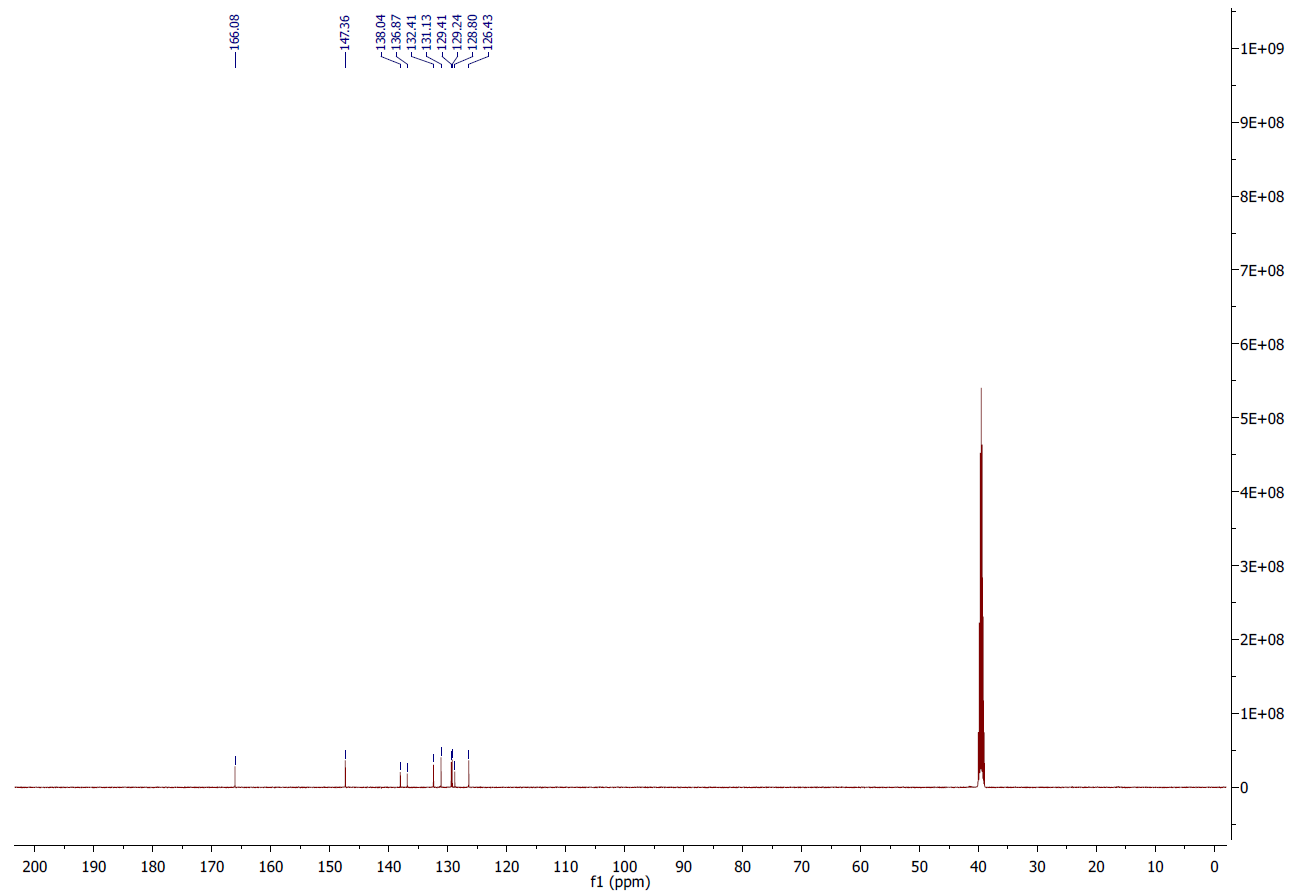


**Figure S5**: ^1^H (**A**) and ^13^C (**B**) NMR spectra of 6-CiQ-COCl in DMSO-*d_6_*.




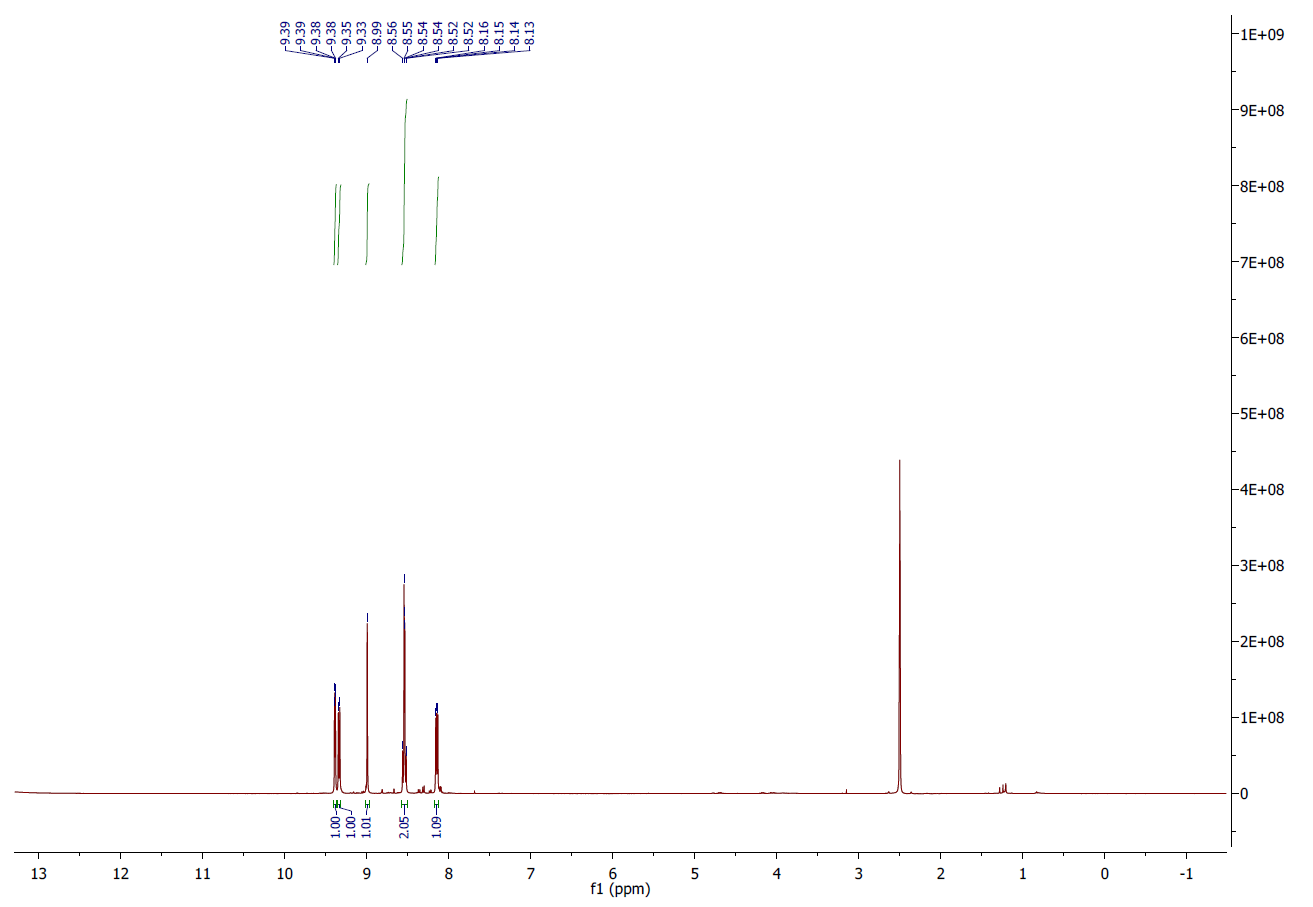


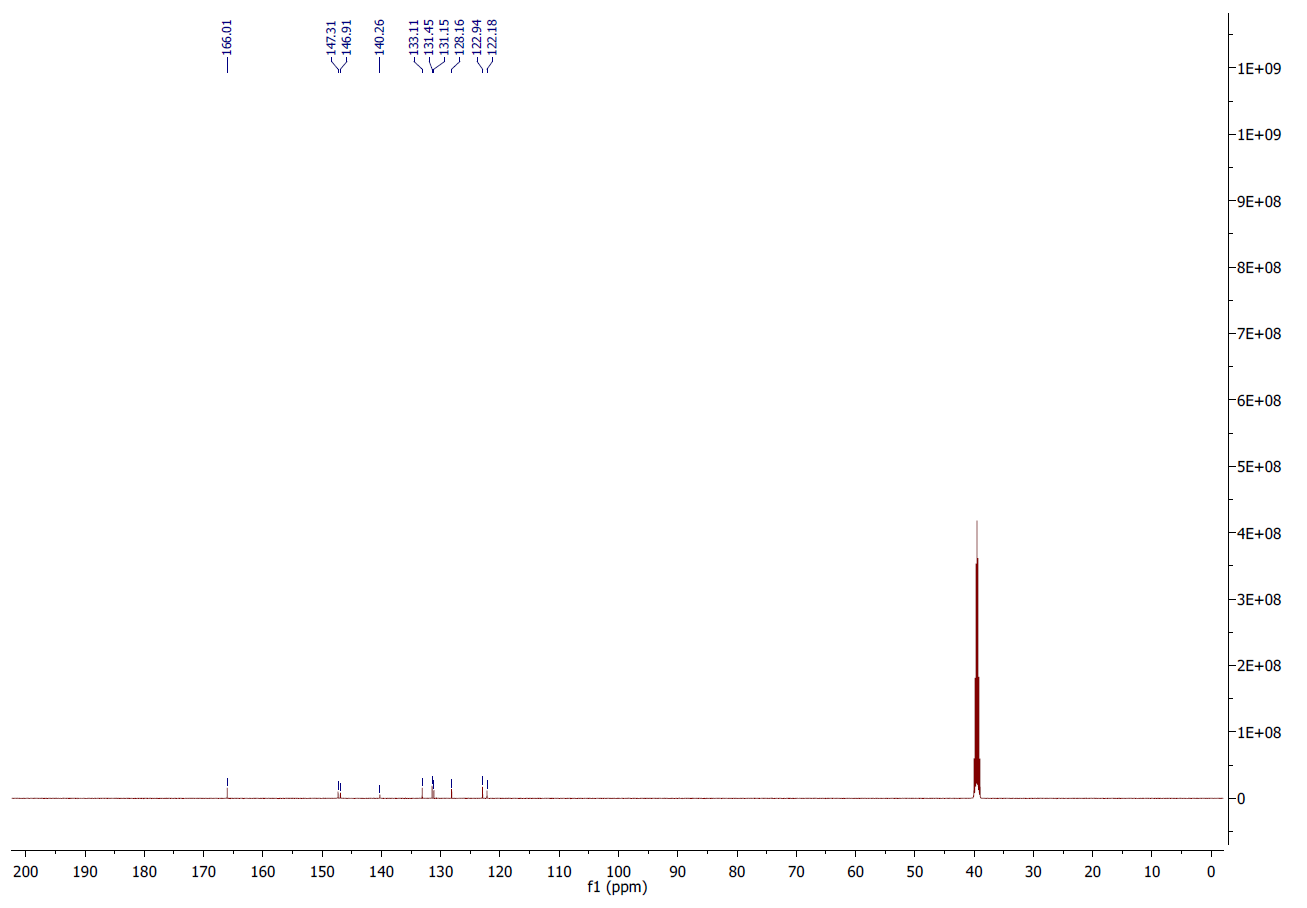


**Figure S6**: ^1^H (**A**) and ^13^C (**B**) NMR spectra of 6-CQ-COCl in DMSO-*d_6_*.




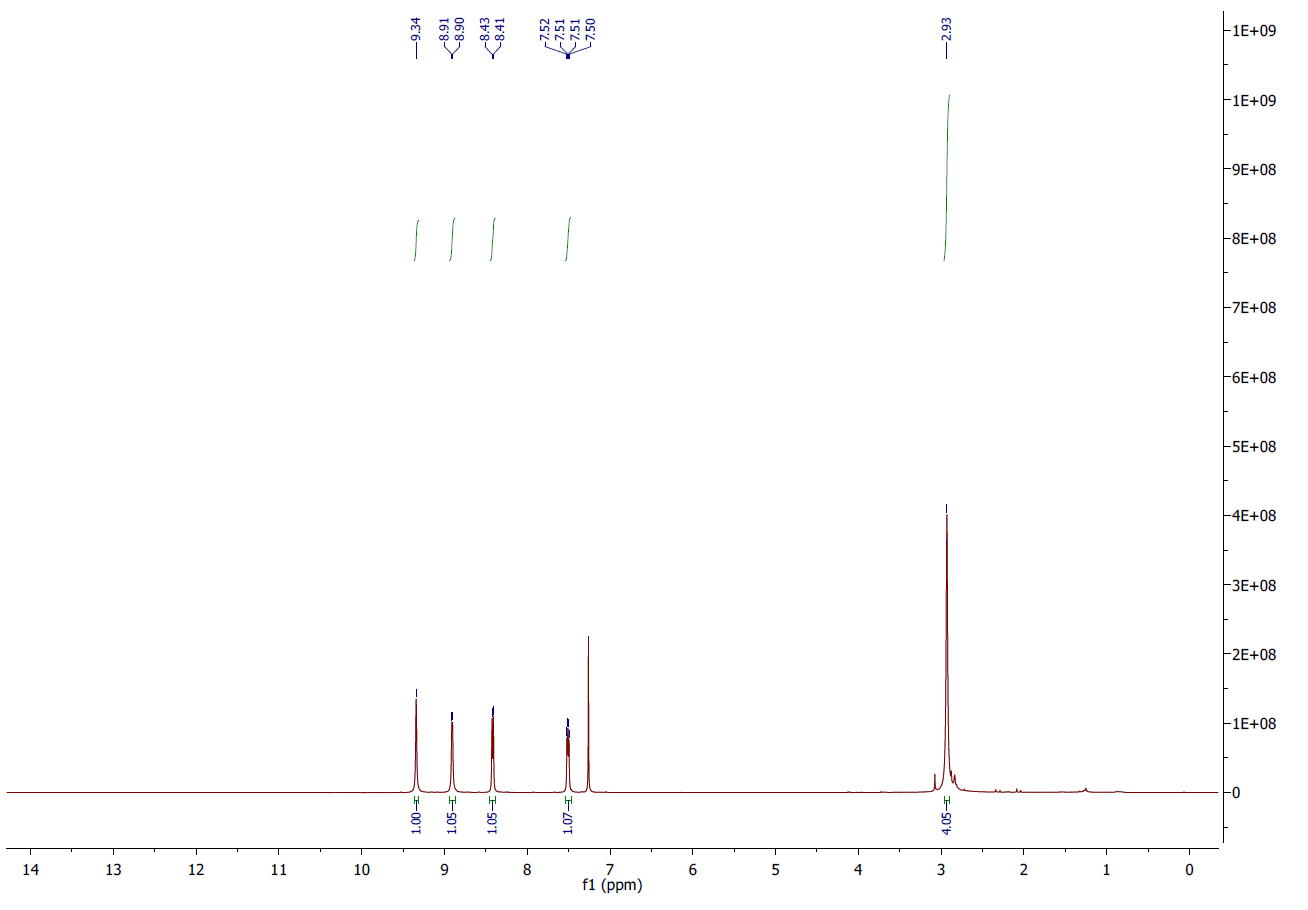


**A**


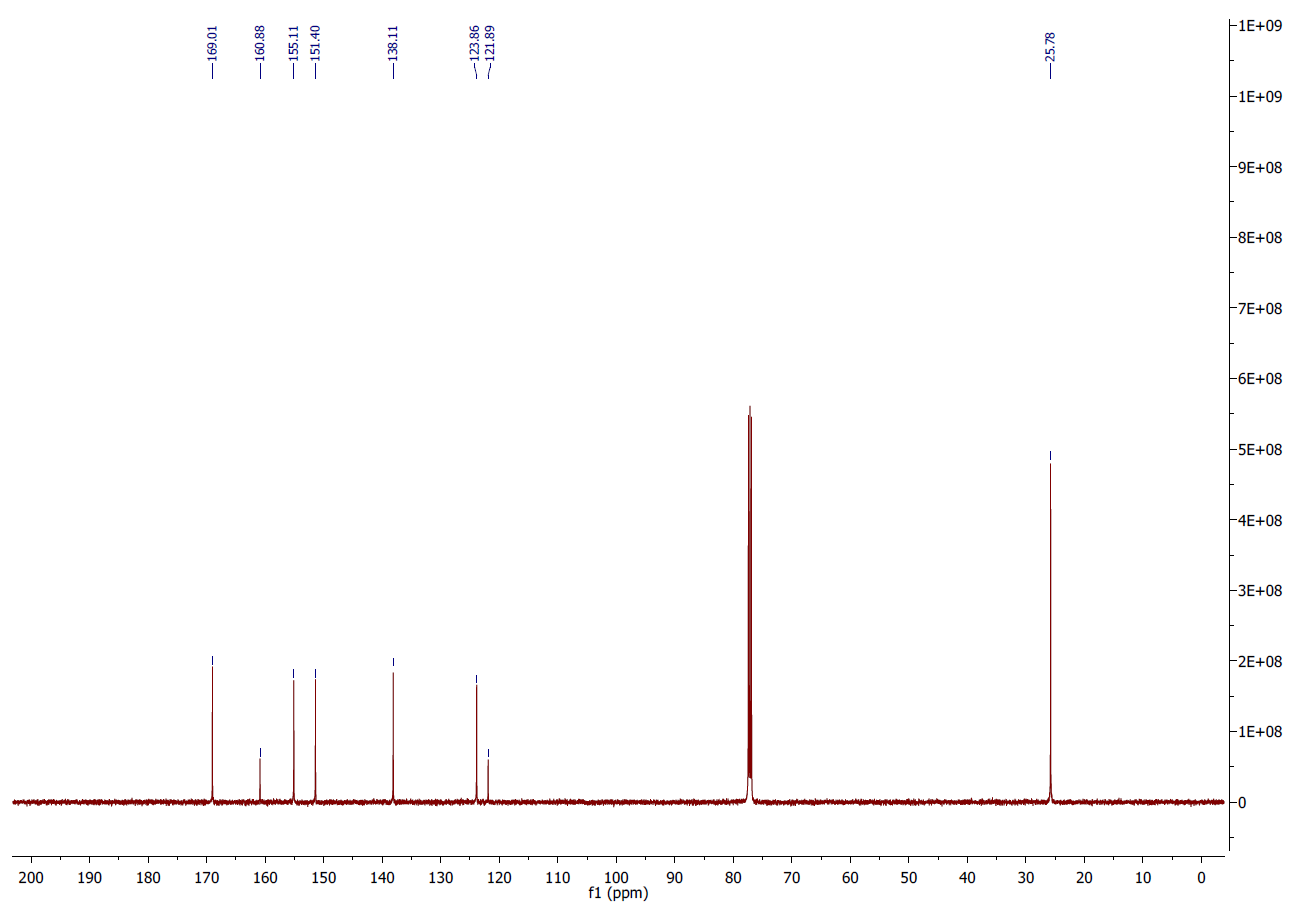


**B**

**Figure S7**: ^1^H (**A**) and ^13^C (**B**) NMR spectra of 3-CP-NHS in CDCl_3_.




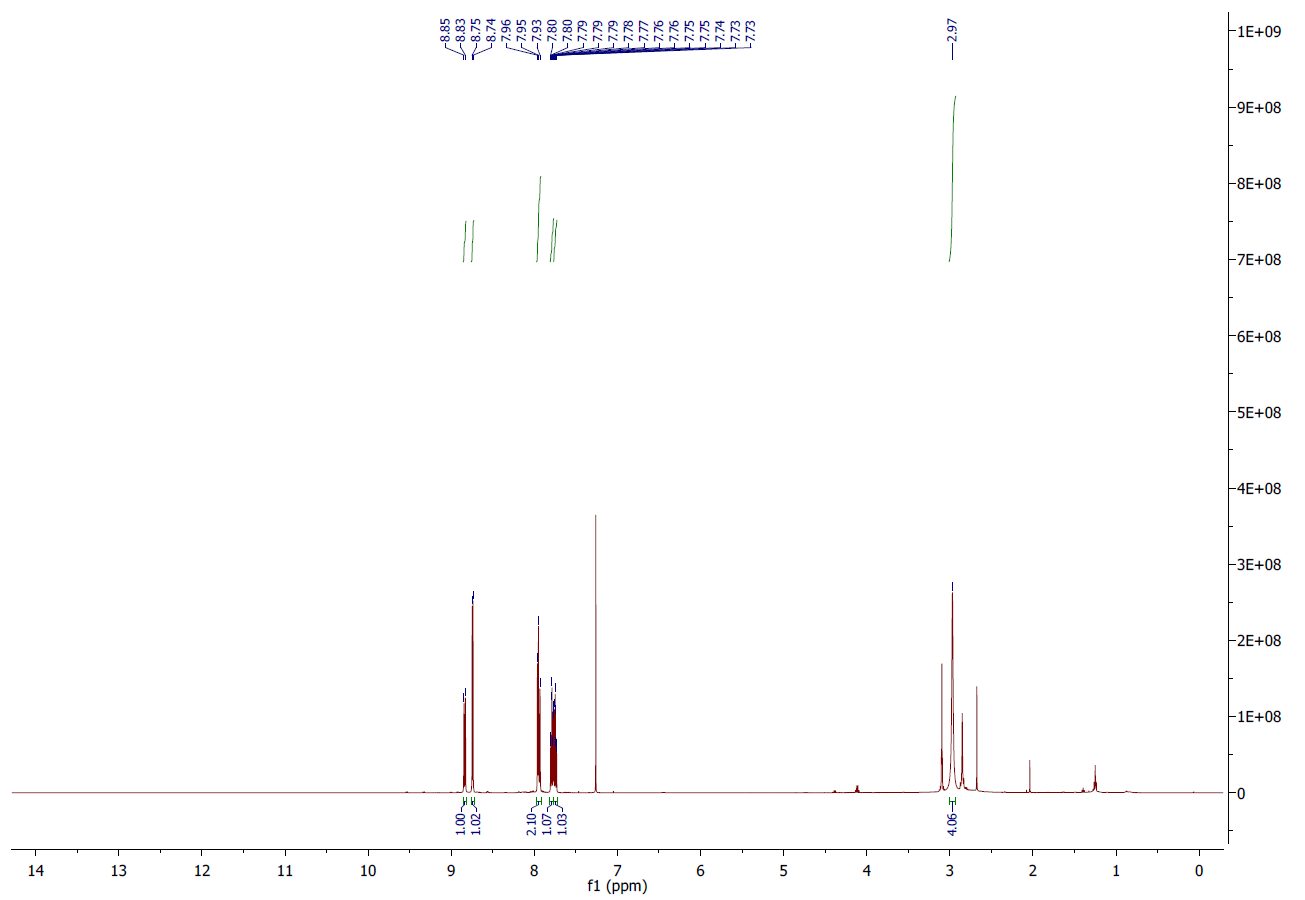


**A**


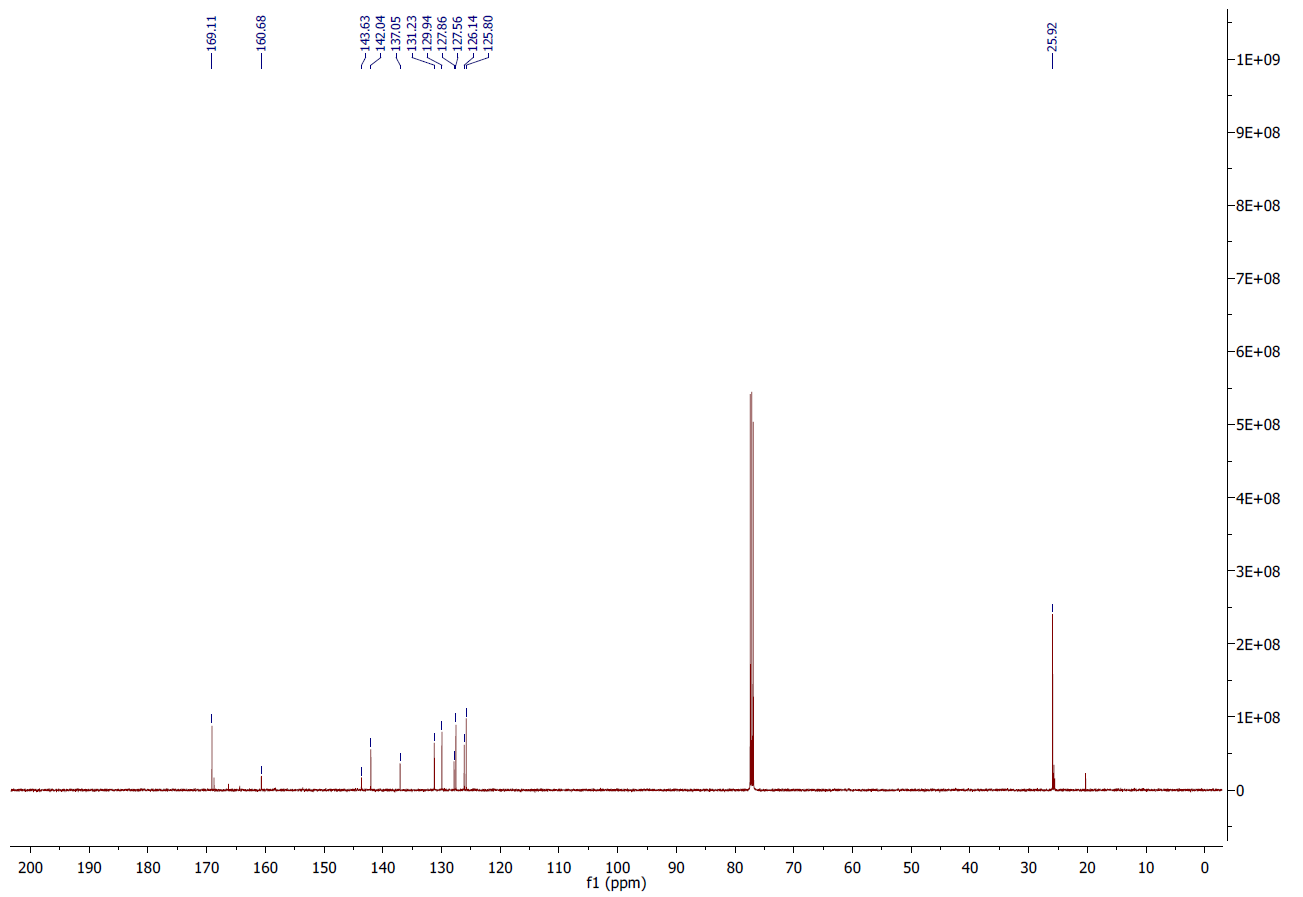


**B**

**Figure S8**: ^1^H (**A**) and ^13^C (**B**) NMR spectra of 1-CiQ-NHS in CDCl_3_.




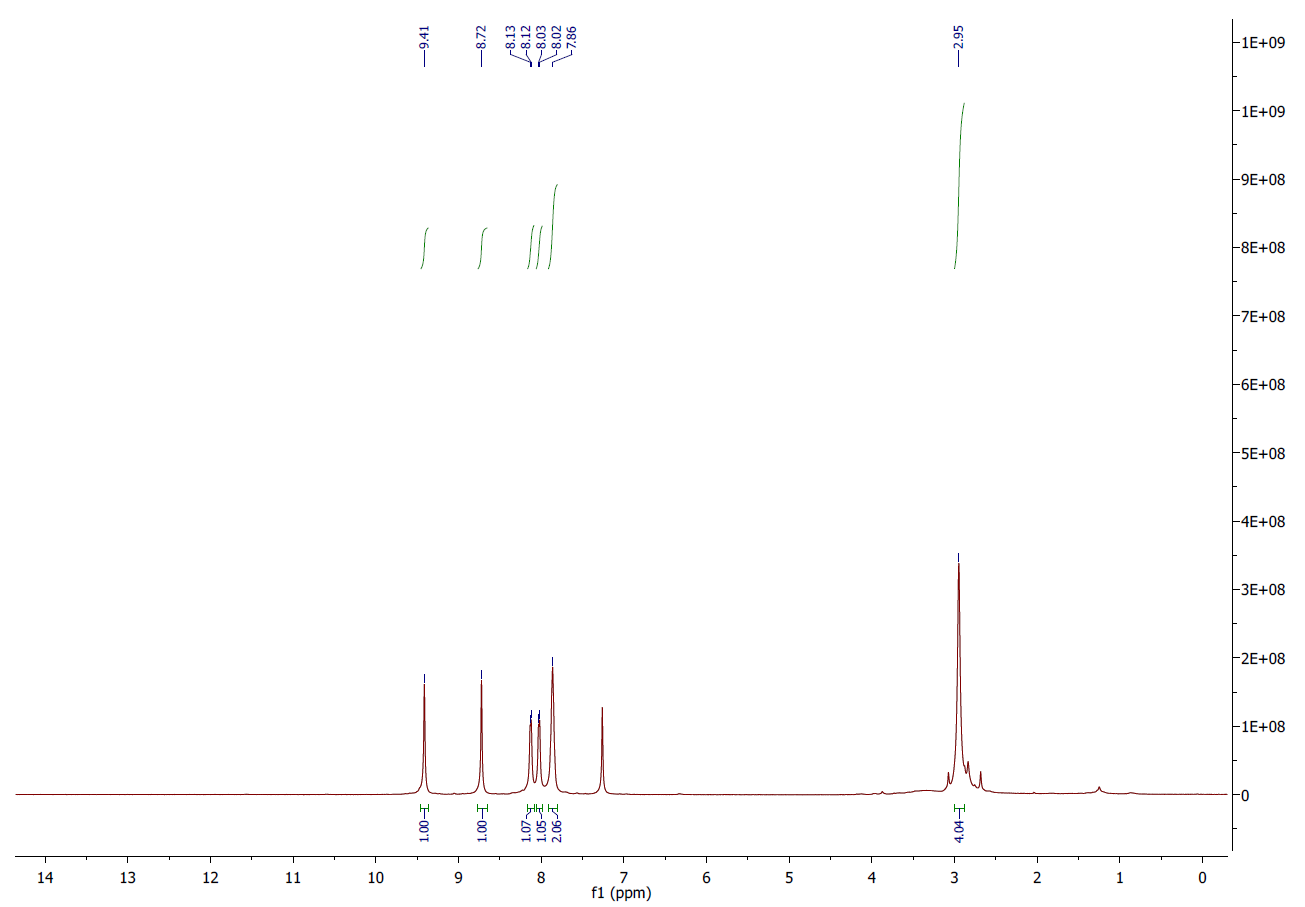


**A**


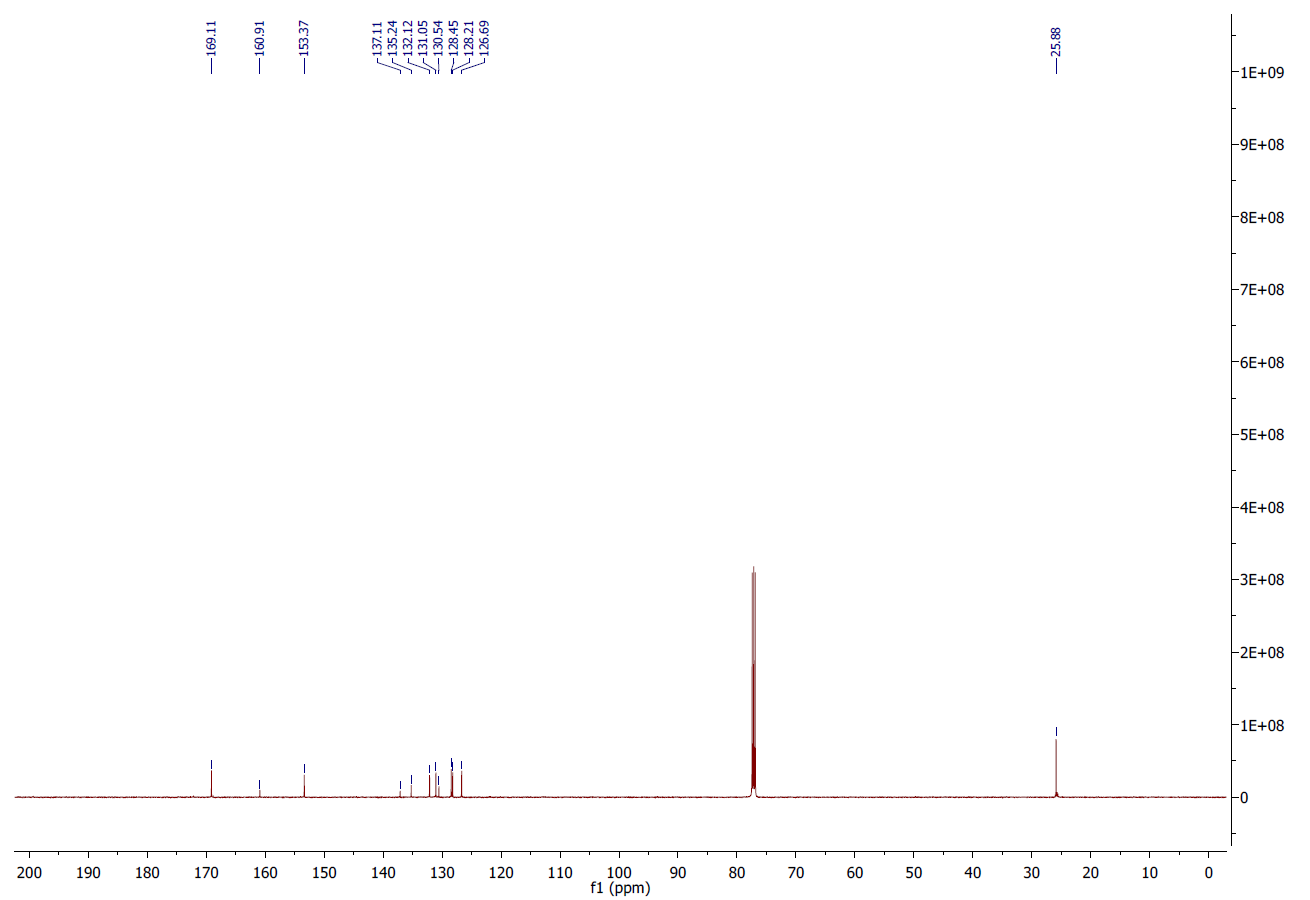


**B**

**Figure S9**: ^1^H (**A**) and ^13^C (**B**) NMR spectra of 3-CiQ-NHS in CDCl_3_.




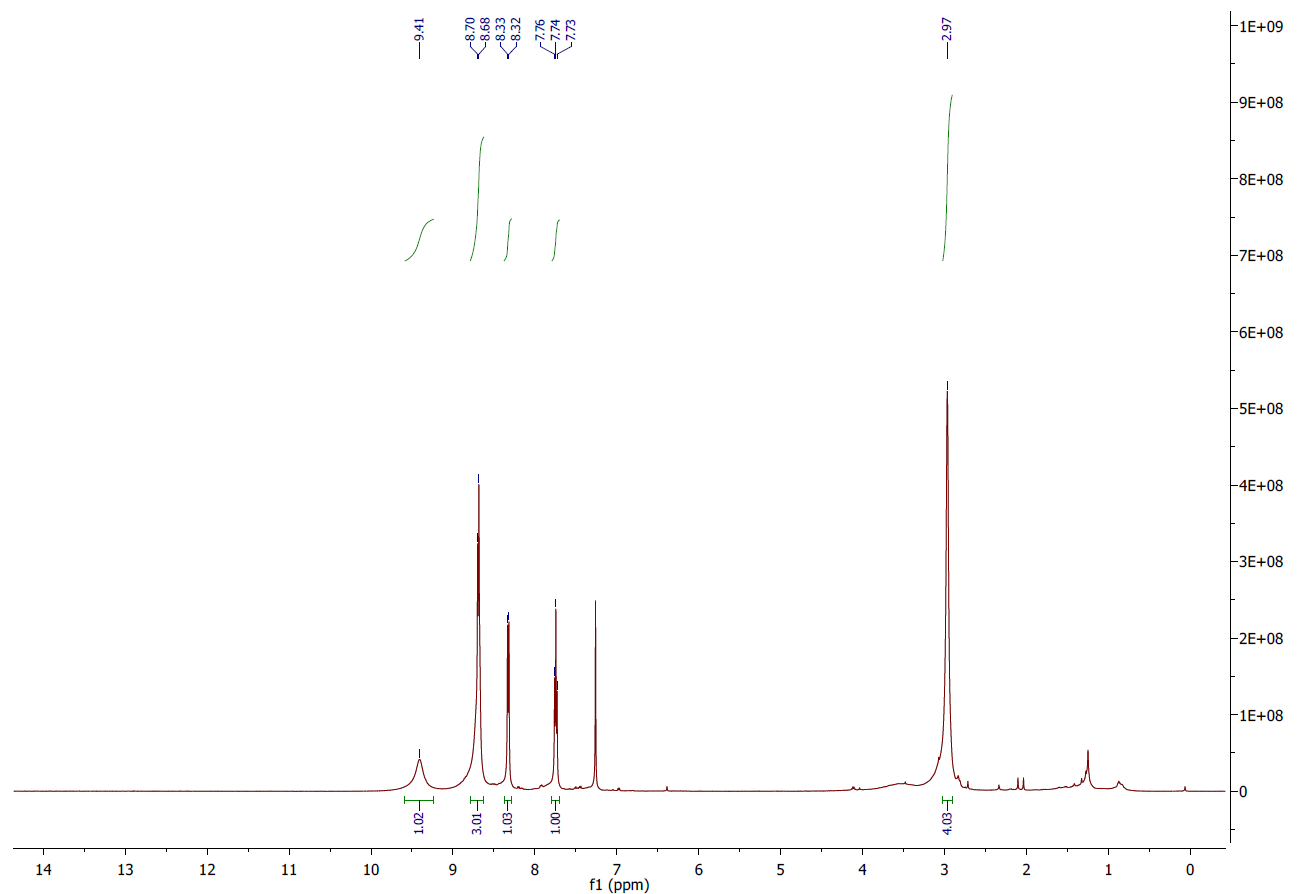


**A**


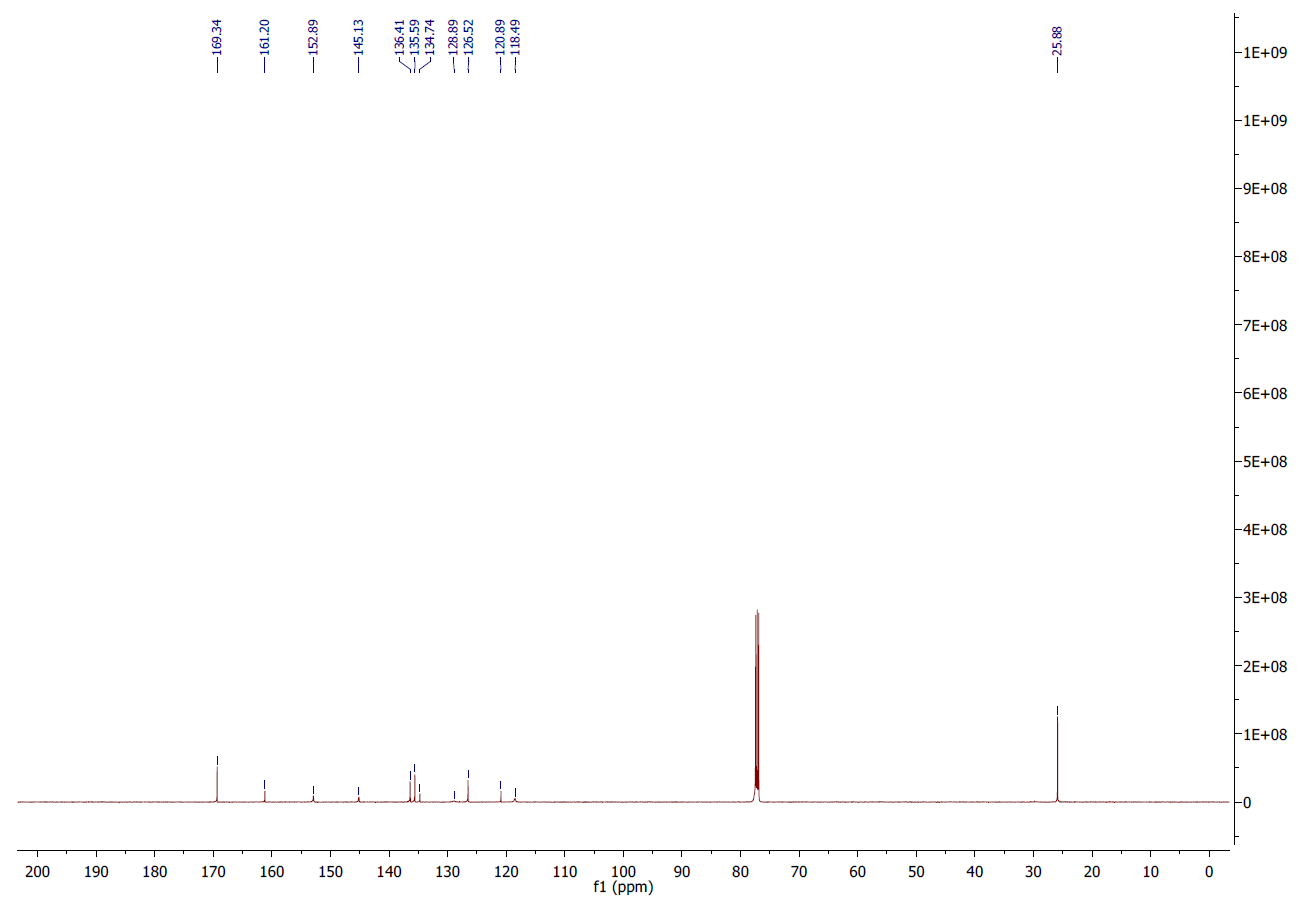


**B**

**Figure S10**: ^1^H (**A**) and ^13^C (**B**) NMR spectra of 5-CiQ-NHS in CDCl_3_.




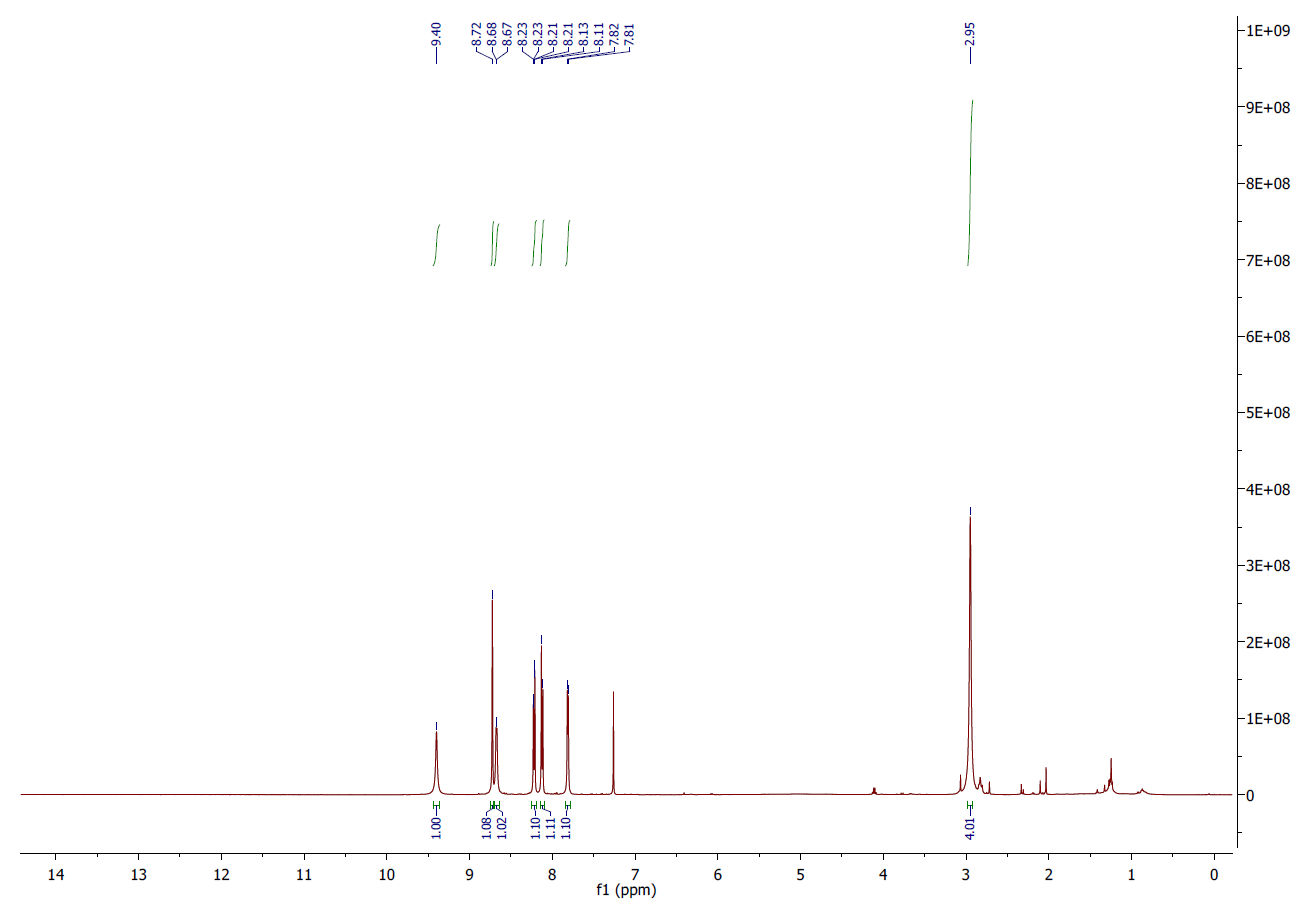


**A**


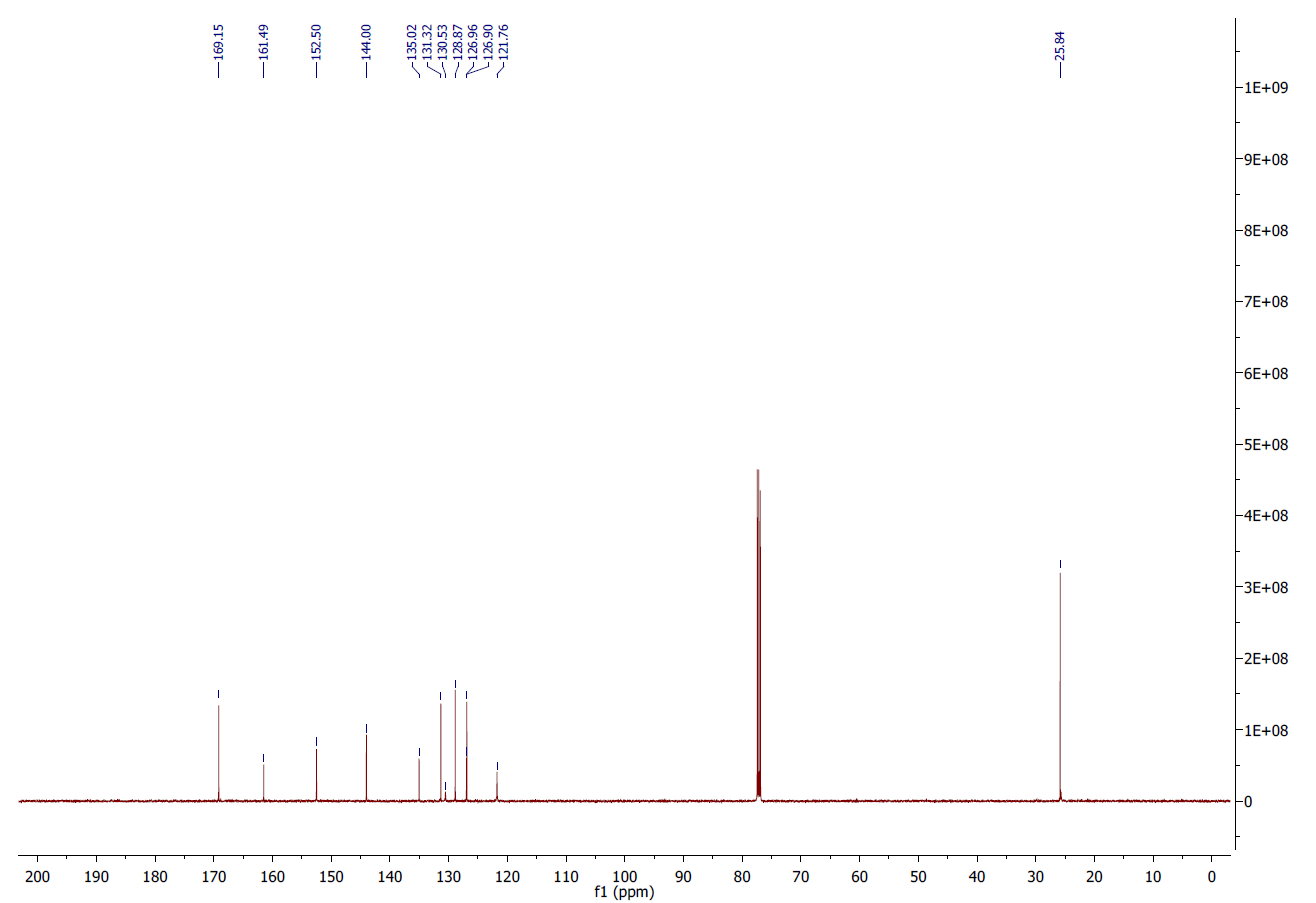


**B**

**Figure S11**: ^1^H (**A**) and ^13^C (**B**) NMR spectra of 6-CiQ-NHS in CDCl_3_.




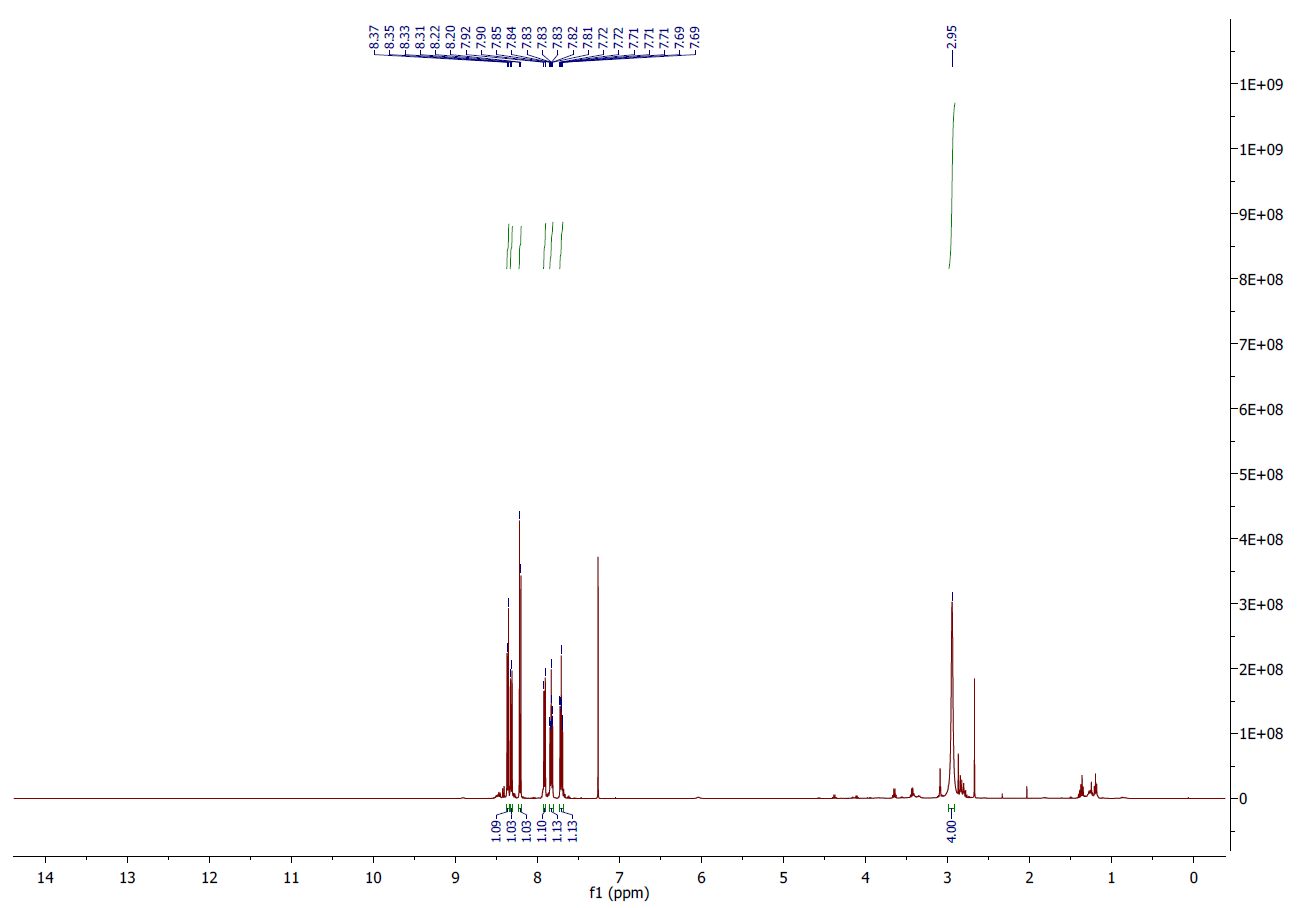


**A**


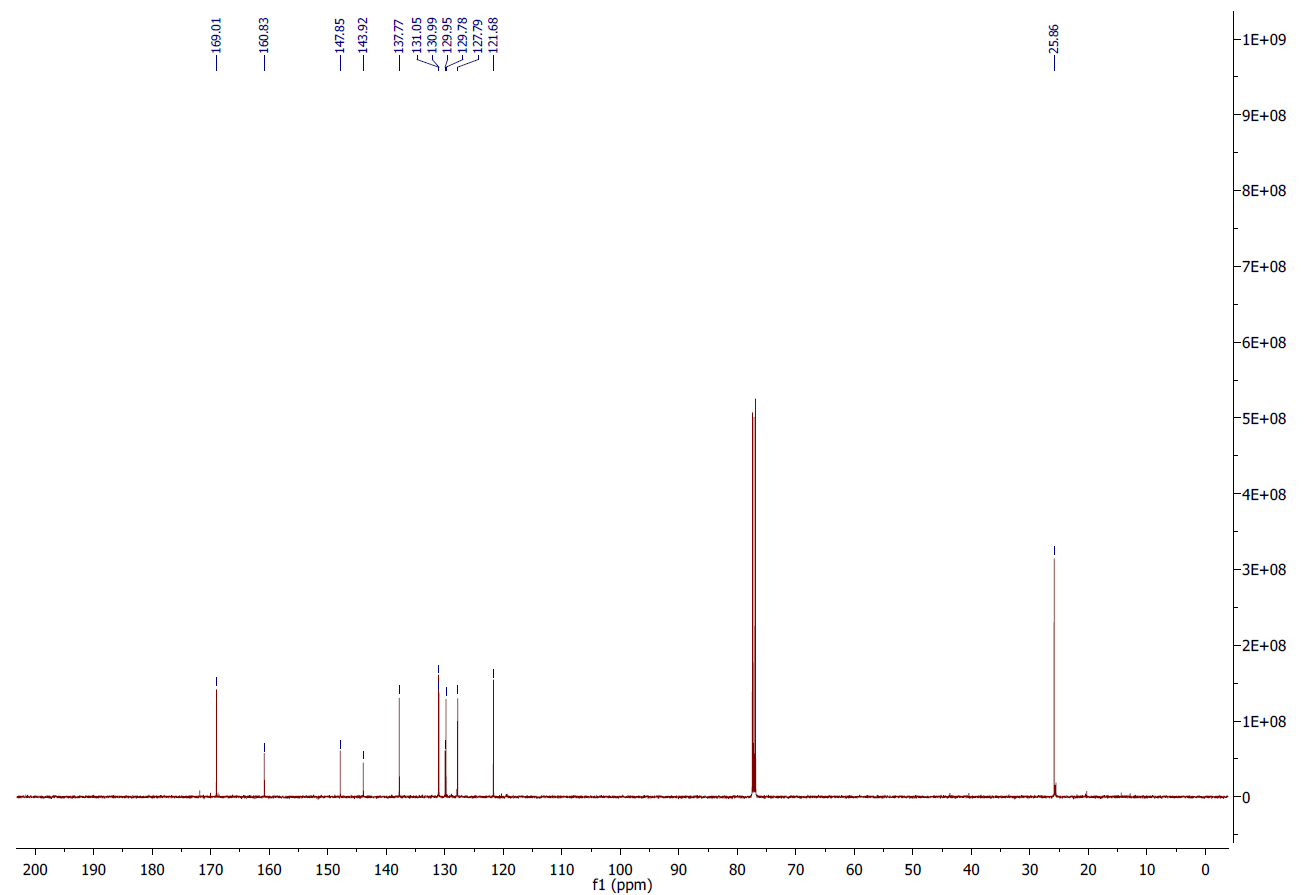


**B**

**Figure S12**: ^1^H (**A**) and ^13^C (**B**) NMR spectra of 2-CQ-NHS in CDCl_3_.




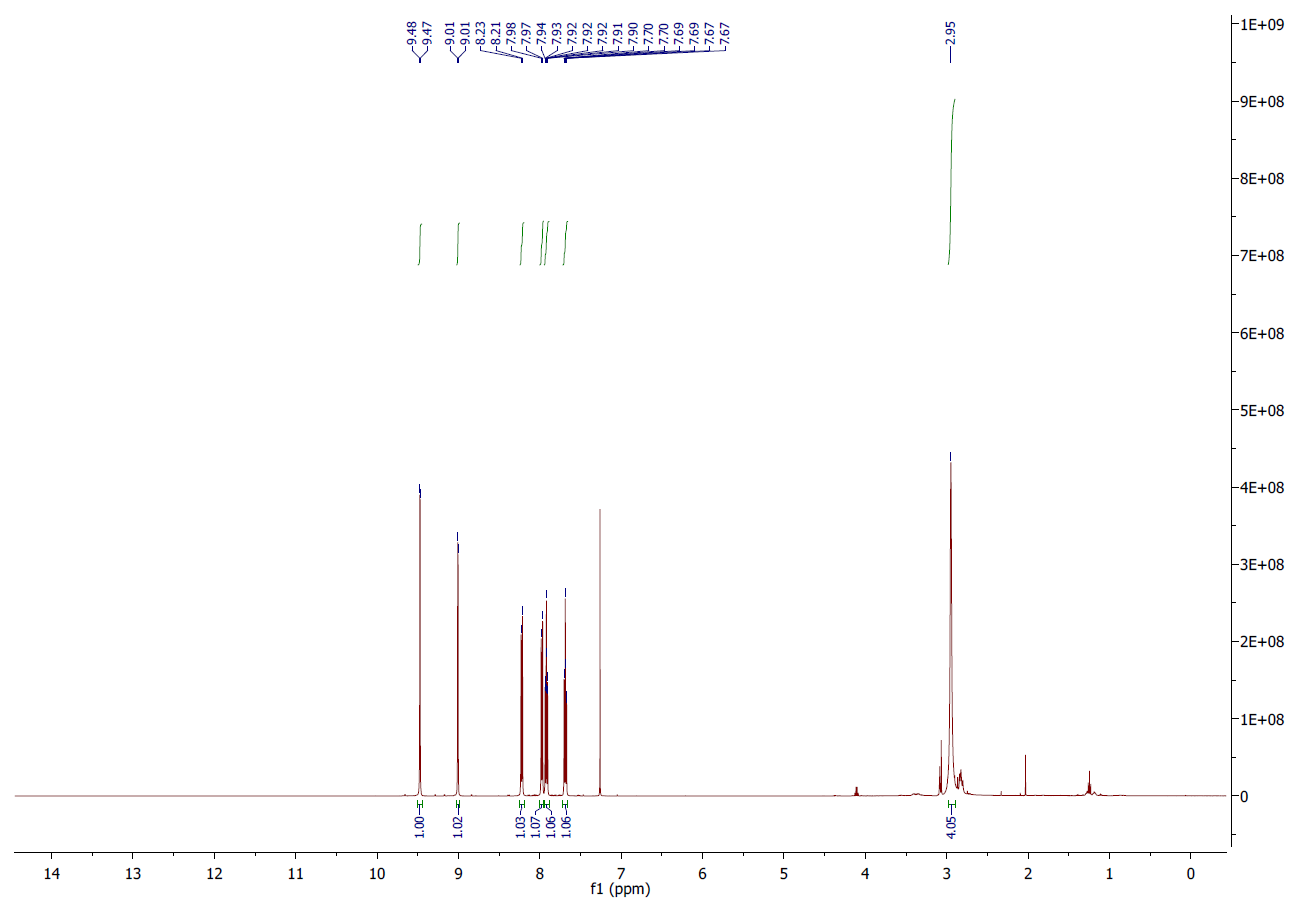


**A**


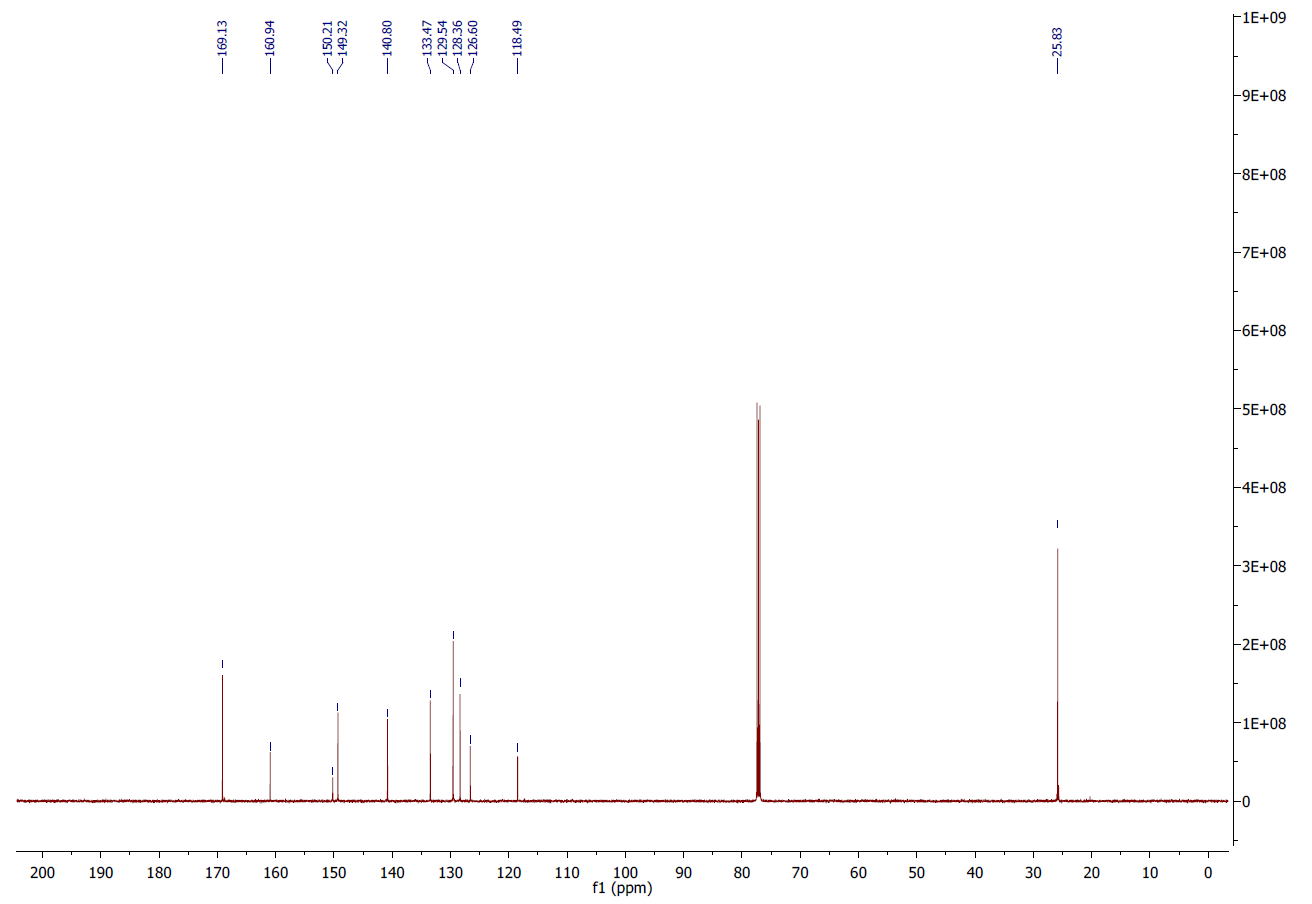


**B**

**Figure S13**: ^1^H (**A**) and ^13^C (**B**) NMR spectra of 3-CQ-NHS in CDCl_3_.




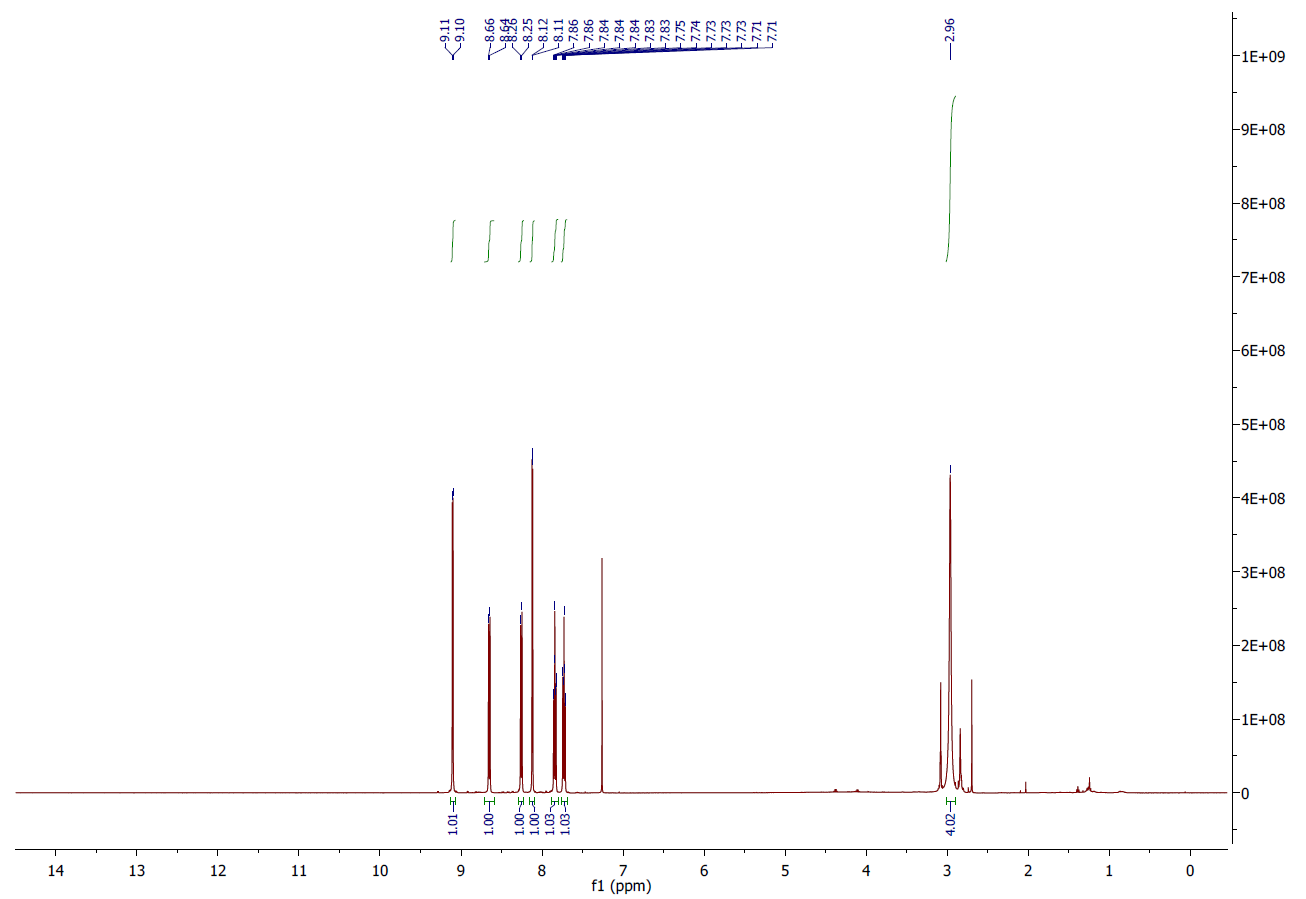


**A**


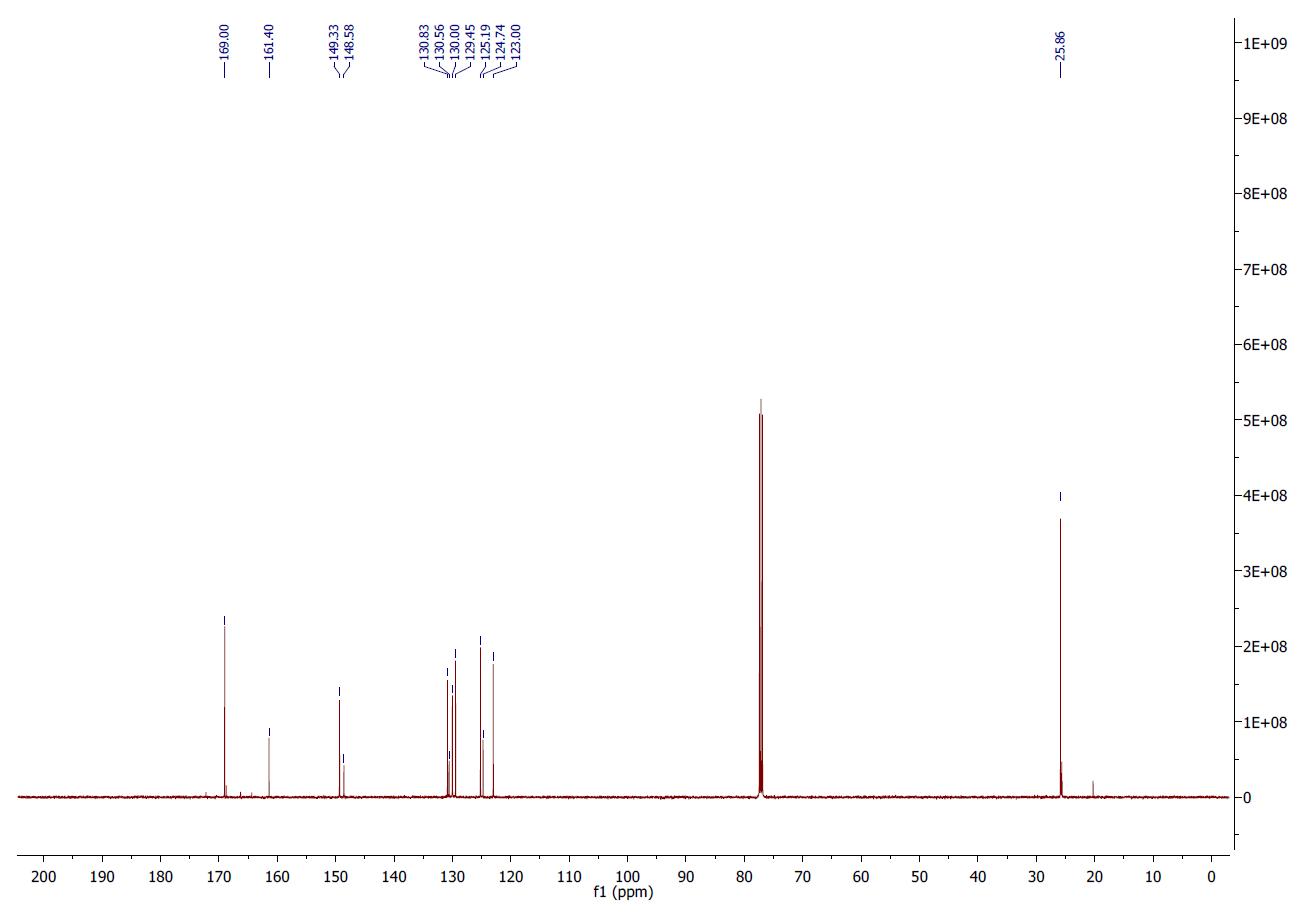


**B**

**Figure S14**: ^1^H (**A**) and ^13^C (**B**) NMR spectra of 4-CQ-NHS in CDCl_3_.




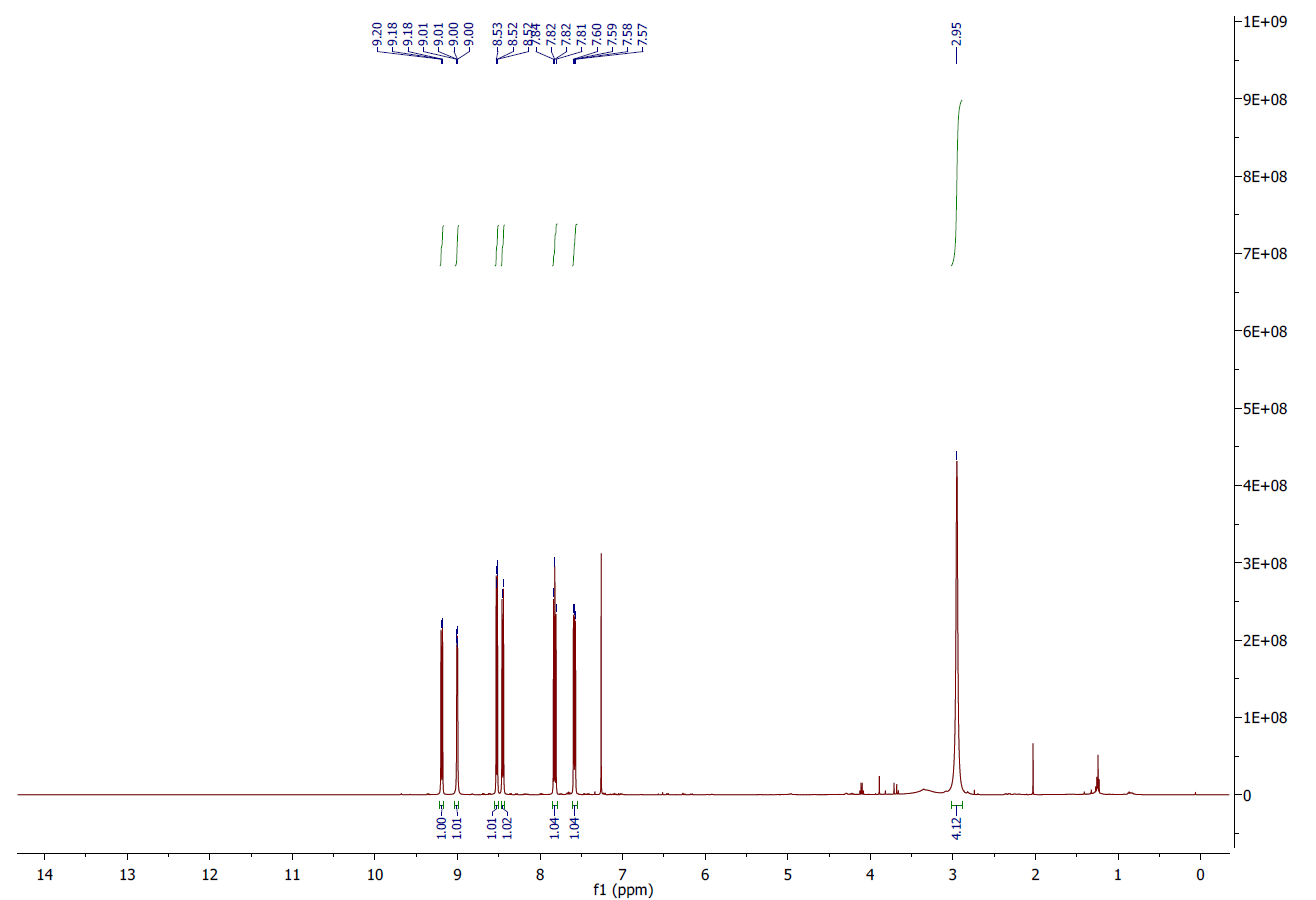


**A**


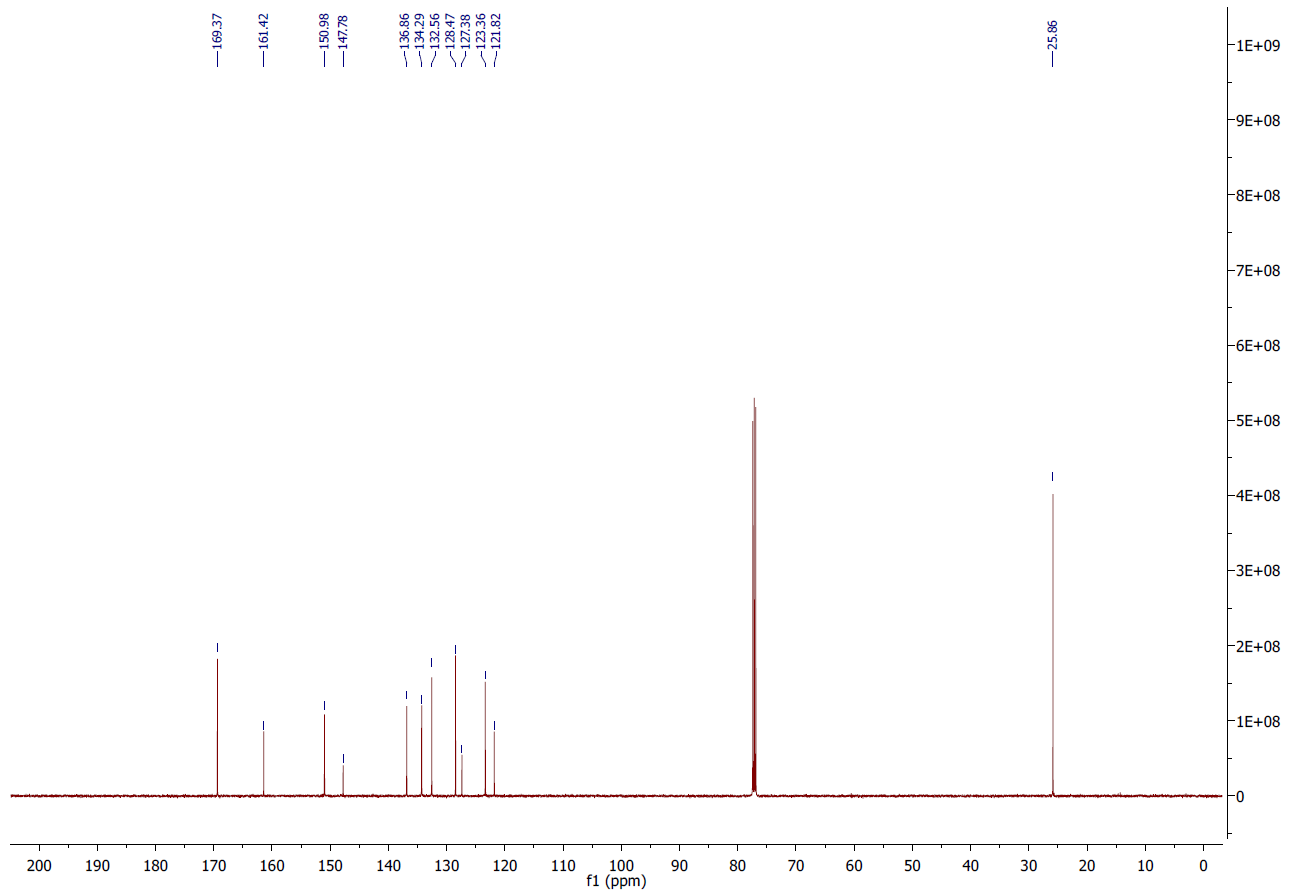


**B**

**Figure S15**: ^1^H (**A**) and ^13^C (**B**) NMR spectra of 5-CQ-NHS in CDCl_3_.




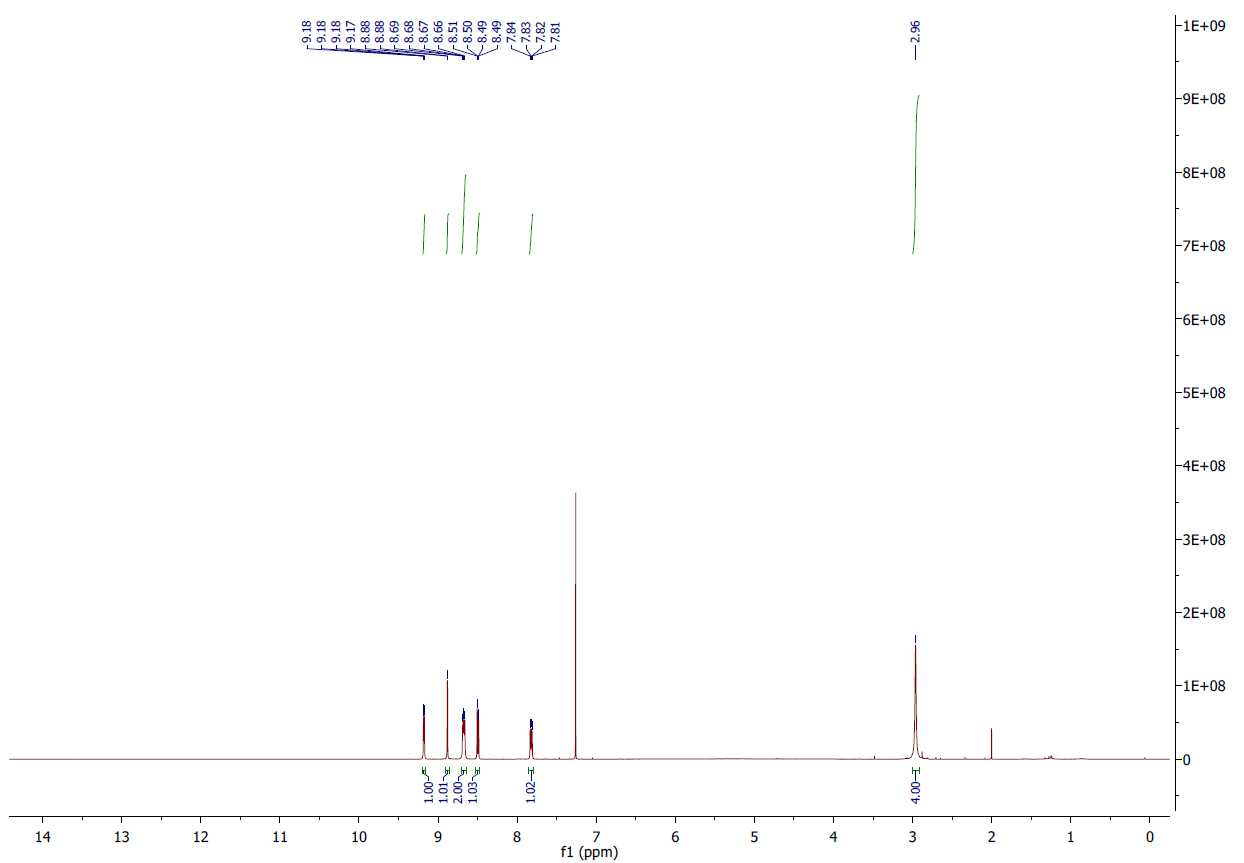


**A**


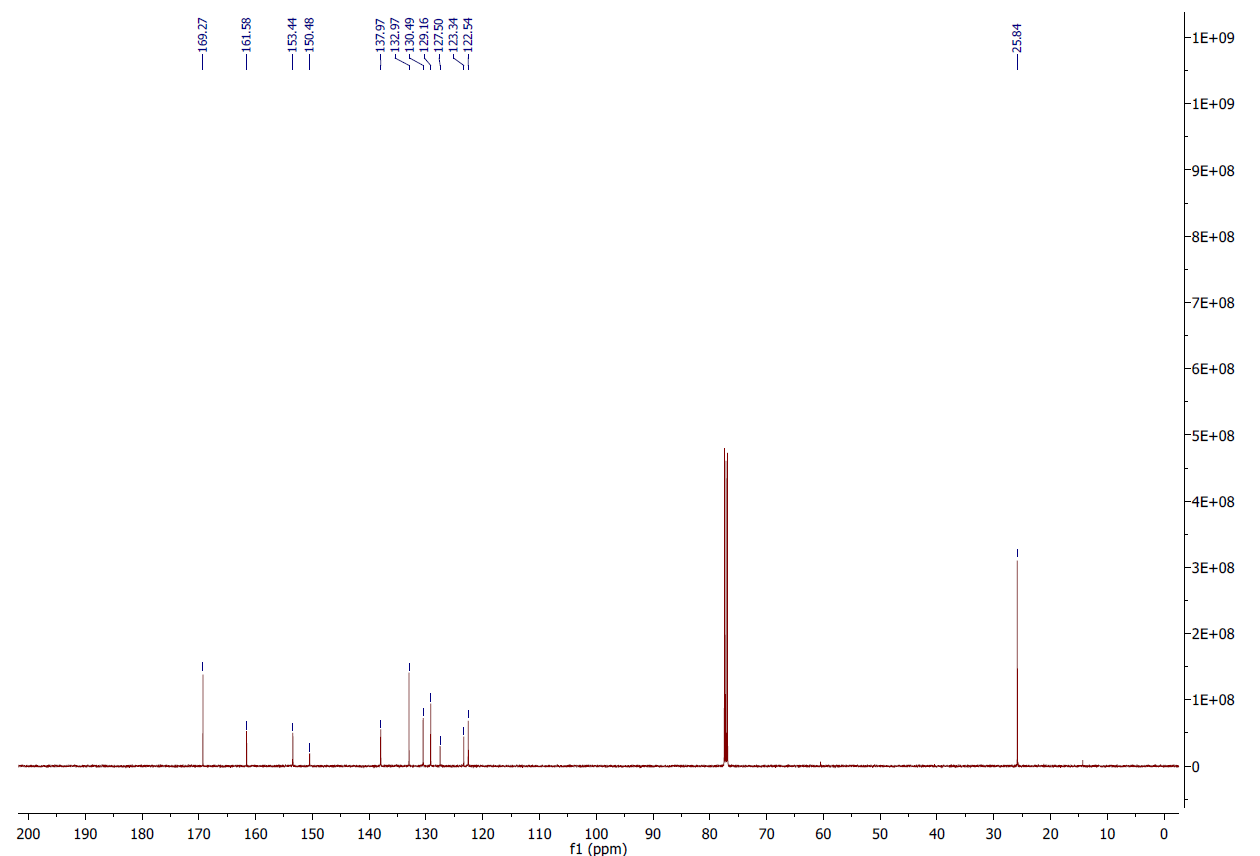


**B**

**Figure S16**: ^1^H (**A**) and ^13^C (**B**) NMR spectra of 6-CQ-NHS in CDCl_3_.




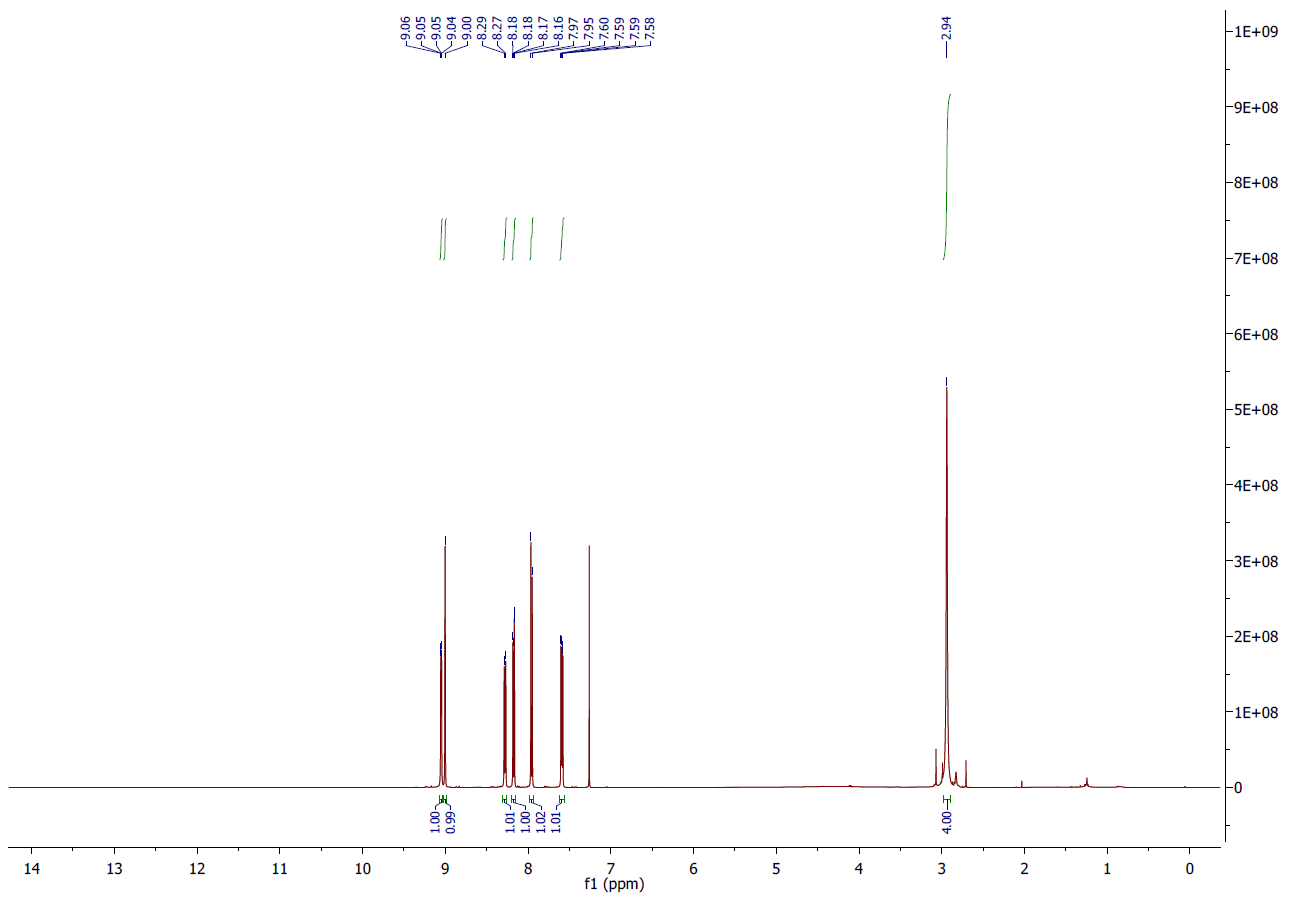


**A**


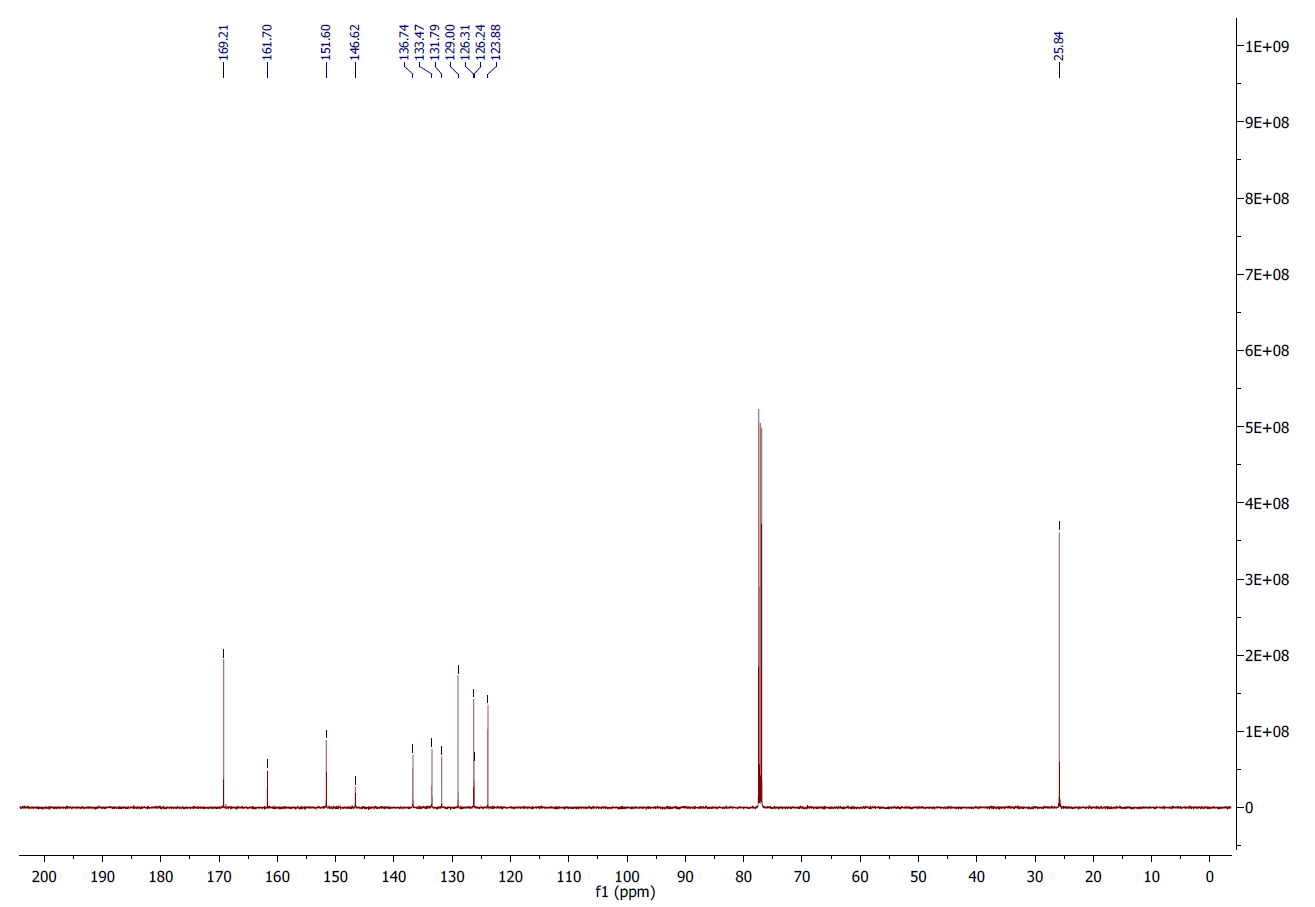


**B**

**Figure S17**: ^1^H (**A**) and ^13^C (**B**) NMR spectra of 7-CQ-NHS in CDCl_3_.




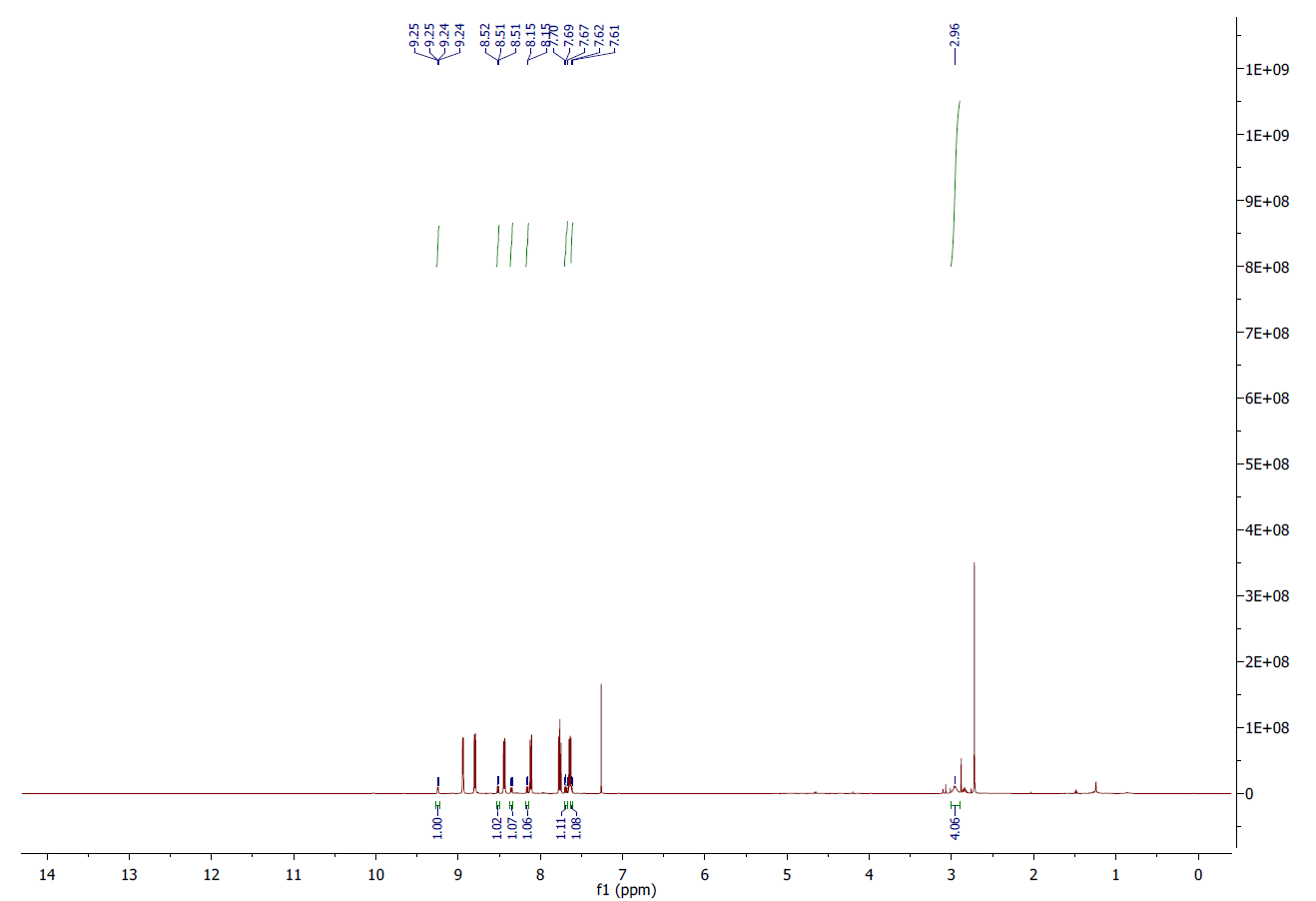


**A**


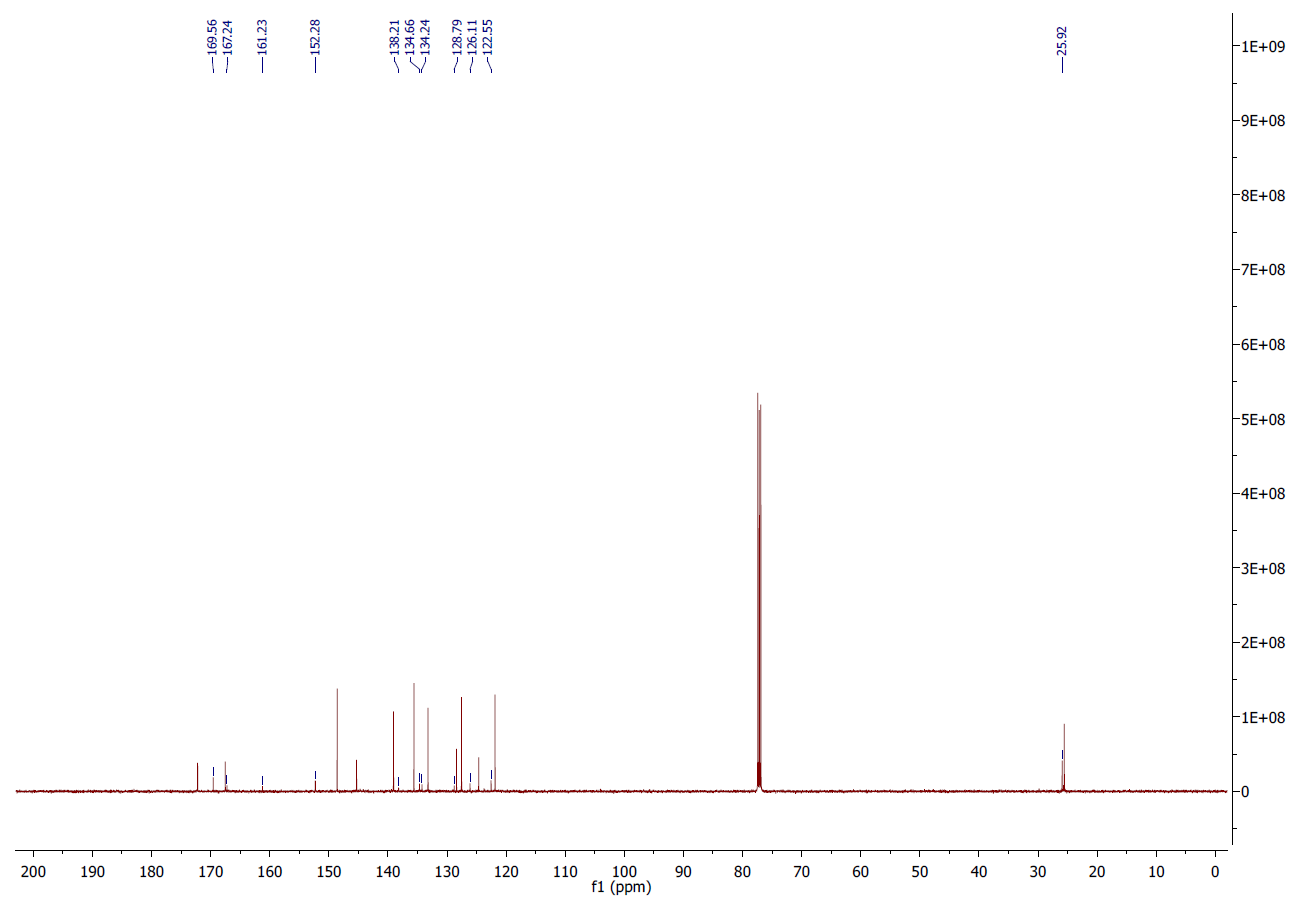


**B**

**Figure S18**: ^1^H (**A**) and ^13^C (**B**) NMR spectra of 8-CQ-NHS in CDCl_3_ (Due to product degradation back to the starting material, the identification of its NMR spectral features was performed by comparison with the NMR spectrum of the 8-CQ-COOH starting material).

## Synthetic reaction scheme





**Figure S19**: Reaction scheme for the preparation of four types of agents’ reactive functional groups: (i) carbonyl chloride, (ii) sulfonyl chloride, (iii) NHS ester, and (iv) carbamate (illustrating concurrent formation of a urea-based byproduct).

# Preliminary methods

## Analysis of derivatized amino acid standards

Preliminary analysis was performed using an Agilent 1290 Infinity II UHPLC system equipped with a diode-array detector (DAD) and coupled to an Agilent 6470 Triple Quadrupole mass spectrometer. Chromatographic separation was achieved on a Zorbax EclipsePlus C18 column (50 × 2.1 mm; 1.8 µm, Agilent) maintained at 40 °C. The mobile phase consisted of (A) 0.1% FA in 5 mM ammonium formate and (B) 0.1% FA in ACN:MeOH (1:1, v/v), delivered at a flow rate of 0.400 mL/min with an injection volume of 0.1 µL. The gradient elution program was set as follows: 0.0 min (5% B); 1.0 min (5% B); 12.9 min (20% B); 13.0 min (95% B); 14.0 min (95% B); 14.1 min (5% B); 15.0 (5% B).

Samples were analysed in positive ion mode using multiple reaction monitoring (MRM). The MS source parameters were operated under the following conditions: drying gas temperature, 300 °C; drying gas flow, 8 L/min; nebulizer pressure, 35 psi; sheath gas temperature, 300 °C; sheath gas flow, 11 L/min; capillary voltage, 3500 V; and nozzle voltage, 0 V.

## Analysis of DNS derivatized amino acids standards

The analysis of all 20 proteinogenic amino acids using the commercially available reagent dansyl chloride (DNS) was performed on the same UHPLC-MS system described in Section 2.1, maintaining an identical flow rate, column temperature, and injection volume. Chromatographic separation was achieved on an ASTRA C18-AQ UHPLC column (100 × 2.1 mm; 2.0 µm, Chromservis). The gradient elution profile was set as follows: 0 min (35% B); 1.0 min (35%B); 12.9 min (60% B); 13 min (95% B); 14.0 min (95% B); 14.1 min (35% B); 15.0 min (35% B).

Samples were analysed in positive ion mode using multiple reaction monitoring (MRM). The MS source parameters were operated under the following conditions: drying gas temperature, 200 °C; drying gas flow, 6 L/min; nebulizer pressure, 15 psi; sheath gas temperature, 300 °C; sheath gas flow, 11 L/min; capillary voltage, 2500 V; and nozzle voltage, 0 V.

# Storage stability of derivatization agents

**Table S2**: Storage stability of selected representatives of sulfonyl and carbonyl chloride, and NHS ester.

| Stability: | **Time [day]** | **5-SiQ-SO_2_Cl** | **3-CP-COCl** | **5-CiQ-COCl** | **6-CiQ-NHS** | **6-CQ-NHS** | **8-CQ-NHS** |
| --- | --- | --- | --- | --- | --- | --- | --- |
| Freezer (-20 °C) | 0 | 100.00 ± 0.00 | 100.00 ± 0.00 | 100.00 ± 0.00 | 100.00 ± 0.00 | 100.00 ± 0.00 | 100.00 ± 0.00 |
|  | 3 | 42.50 ± 3.10 | 1.50 ± 0.50 | 3.80 ± 1.20 | 99.80 ± 0.20 | 99.60 ± 0.30 | 98.40 ± 0.60 |
|  | 7 | 4.20 ± 0.80 | 0.00 ± 0.00 | 0.00 ± 0.00 | 99.50 ± 0.40 | 99.10 ± 0.50 | 96.20 ± 1.10 |
|  | 30 | 0.00 ± 0.00 | 0.00 ± 0.00 | 0.00 ± 0.00 | 99.10 ± 0.70 | 98.95 ± 0.82 | 79.50 ± 2.43 |
|  | 180 | 0.00 ± 0.00 | 0.00 ± 0.00 | 0.00 ± 0.00 | 98.80 ± 1.20 | 96.10 ± 1.15 | 32.30 ± 4.15 |
|  | 365 | 0.00 ± 0.00 | 0.00 ± 0.00 | 0.00 ± 0.00 | 98.60 ± 1.05 | 94.80 ± 1.80 | 8.90 ± 2.30 |

# Solvent stability of derivatization agents

**Table S3**: Stability of sulfonyl chloride and carbonyl chloride-based agents in ACN and 1:1 ACN:tetraborate buffer mixture.

| Stability in: | **Time [hrs]** | **5-SiQ-SO_2_Cl** | **3-CP-COCl** | **5-CiQ-COCl** | **6-CiQ-COCl** | **6-CQ-COCl** |
| --- | --- | --- | --- | --- | --- | --- |
| ACN | 0 | 100.00 ± 0.00 | 100.00 ± 0.00 | 100.00 ± 0.00 | 100.00 ± 0.00 | 100.00 ± 0.00 |
|  | 0.5 | 100.00 ± 0.00 | 59.38 ± 12.72 | 51.27 ± 3.02 | 18.20 ± 2.38 | 97.80 ± 3.80 |
|  | 1 | 98.74 ± 2.18 | 55.93 ± 0.17 | 17.90 ± 3.41 | 11.62 ± 0.23 | 52.25 ± 4.63 |
|  | 2 | 100.00 ± 0.00 | 56.82 ± 5.26 | 3.84 ± 0.49 | 9.54 ± 2.81 | 5.55 ± 0.62 |
|  | 4 | 100.00 ± 0.00 | 54.48 ± 4.70 | 2.93 ± 0.10 | 9.92 ± 1.20 | 0.28 ± 0.15 |
|  | 6 | 100.00 ± 0.00 | 50.00 ± 10.66 | 2.93 ± 0.71 | 8.94 ± 5.99 | 0.07 ± 0.04 |
|  | 24 | 100.00 ± 0.00 | 2.94 ± 0.30 | 1.09 ± 1.11 | 5.47 ± 5.11 | 0.26 ± 0.24 |
| ACN:buffer | 0 | 100.00 ± 0.00 | 100.00 ± 0.00 | 100.00 ± 0.00 | 100.00 ± 0.00 | 100.00 ± 0.00 |
|  | 0.5 | 40.80 ± 0.48 | 0.86 ± 0.45 | 34.77 ± 2.51 | 50.31 ± 5.25 | 26.22 ± 1.71 |
|  | 1 | 12.55 ± 0.98 | 3.32 ± 3.79 | 24.03 ± 1.75 | 26.87 ± 6.26 | 0.48 ± 0.42 |
|  | 2 | 0.62 ± 0.71 | 4.40 ± 3.61 | 7.19 ± 2.25 | 1.52 ± 1.42 | 8.38 ± 0.08 |
|  | 4 | 0.12 ± 0.08 | 0.00 ± 0.00 | 7.09 ± 1.90 | 1.21 ± 1.16 | 0.46 ± 0.45 |
|  | 6 | 0.30 ± 0.16 | 0.00 ± 0.00 | 7.60 ± 3.22 | 0.70 ± 0.08 | 0.45 ± 0.37 |
|  | 24 | 0.15 ± 0.08 | 0.00 ± 0.00 | 0.00 ± 0.00 | 0.45 ± 0.41 | 0.11 ± 0.09 |

**Table S4**: Stability of isoquinoline NHS ester-based agents in ACN and 1:1 ACN:tetraborate buffer mixture

| Stability in: | **Time [hrs]** | **3-CP-NHS** | **1-CiQ-NHS** | **3-CiQ-NHS** | **5-CiQ-NHS** | **6-CiQ-NHS** |
| --- | --- | --- | --- | --- | --- | --- |
| ACN | 0 | 100.00 ± 0.00 | 100.00 ± 0.00 | 100.00 ± 0.00 | 100.00 ± 0.00 | 100.00 ± 0.00 |
|  | 0.5 | 99.89 ± 0.18 | 98.27 ± 3.00 | 99.68 ± 0.32 | 99.38 ± 1.07 | 99.41 ± 1.03 |
|  | 1 | 100.00 ± 0.00 | 97.91 ± 3.63 | 99.37 ± 1.05 | 100.00 ± 0.00 | 100.00 ± 0.00 |
|  | 2 | 99.97 ± 0.05 | 98.07 ± 3.34 | 99.73 ± 0.47 | 99.48 ± 0.90 | 100.00 ± 0.00 |
|  | 4 | 100.00 ± 0.00 | 98.19 ± 3.13 | 98.44 ± 1.87 | 99.47 ± 0.92 | 99.20 ± 1.39 |
|  | 6 | 100.00 ± 0.00 | 98.09 ± 3.30 | 100.00 ± 0.00 | 99.92 ± 0.14 | 97.62 ± 4.12 |
|  | 12 | 100.00 ± 0.00 | 99.02 ± 0.86 | 99.24 ± 0.91 | 92.49 ± 0.22 | 98.73 ± 1.58 |
|  | 24 | 100.00 ± 0.00 | 98.61 ± 2.40 | 100.00 ± 0.00 | 86.64 ± 3.71 | 94.69 ± 6.51 |
| ACN:buffer | 0 | 100.00 ± 0.00 | 100.00 ± 0.00 | 100.00 ± 0.00 | 100.00 ± 0.00 | 100.00 ± 0.00 |
|  | 0.5 | 75.92 ± 4.76 | 78.82 ± 9.77 | 100.00 ± 0.00 | 75.10 ± 3.52 | 69.84 ± 3.07 |
|  | 1 | 71.60 ± 4.56 | 65.43 ± 10.16 | 100.00 ± 0.00 | 64.71 ± 4.03 | 62.02 ± 2.59 |
|  | 2 | 67.64 ± 3.35 | 55.36 ± 11.17 | 96.60 ± 0.92 | 58.44 ± 2.43 | 58.49 ± 2.62 |
|  | 4 | 59.96 ± 4.03 | 37.31 ± 10.23 | 91.50 ± 3.27 | 47.27 ± 4.00 | 37.92 ± 5.28 |
|  | 6 | 55.68 ± 4.79 | 30.12 ± 8.88 | 80.57 ± 4.37 | 43.02 ± 1.32 | 33.09 ± 4.22 |
|  | 12 | 46.24 ± 4.15 | 9.80 ± 5.26 | 63.27 ± 5.60 | 28.48 ± 0.97 | 24.01 ± 2.81 |
|  | 24 | 33.96 ± 4.41 | 0.27 ± 0.18 | 42.17 ± 5.09 | 12.85 ± 1.28 | 10.48 ± 1.31 |

**Table S5**: Stability of quinoline NHS ester agents in ACN and 1:1 ACN:tetraborate buffer mixture

| Stability in: | **Time [hrs]** | **2-CQ-NHS** | **3-CQ-NHS** | **4-CQ-NHS** | **5-CQ-NHS** | **6-CQ-NHS** | **7-CQ-NHS** |
| --- | --- | --- | --- | --- | --- | --- | --- |
| ACN | 0 | 100.00 ± 0.00 | 100.00 ± 0.00 | 100.00 ± 0.00 | 100.00 ± 0.00 | 100.00 ± 0.00 | 100.00 ± 0.00 |
|  | 0.5 | 100.00 ± 0.00 | 99.78 ± 0.38 | 100.00 ± 0.00 | 100.00 ± 0.00 | 100.00 ± 0.00 | 100.00 ± 0.00 |
|  | 1 | 100.00 ± 0.00 | 99.63 ± 0.65 | 100.00 ± 0.00 | 99.97 ± 0.06 | 100.00 ± 0.00 | 99.83 ± 0.29 |
|  | 2 | 99.98 ± 0.03 | 99.38 ± 0.94 | 99.98 ± 0.03 | 100.00 ± 0.00 | 100.00 ± 0.00 | 99.65 ± 0.60 |
|  | 4 | 100.00 ± 0.00 | 99.02 ± 1.14 | 100.00 ± 0.00 | 100.00 ± 0.00 | 100.00 ± 0.00 | 100.00 ± 0.00 |
|  | 6 | 100.00 ± 0.00 | 97.01 ± 1.84 | 100.00 ± 0.00 | 100.00 ± 0.00 | 100.00 ± 0.00 | 100.00 ± 0.00 |
|  | 12 | 99.88 ± 0.21 | 97.42 ± 1.61 | 100.00 ± 0.00 | 100.00 ± 0.00 | 100.00 ± 0.00 | 100.00 ± 0.00 |
|  | 24 | 100.00 ± 0.00 | 98.24 ± 2.29 | 100.00 ± 0.00 | 100.00 ± 0.00 | 100.00 ± 0.00 | 100.00 ± 0.00 |
| ACN:buffer | 0 | 100.00 ± 0.00 | 100.00 ± 0.00 | 100.00 ± 0.00 | 100.00 ± 0.00 | 100.00 ± 0.00 | 100.00 ± 0.00 |
|  | 0.5 | 89.94 ± 1.12 | 55.01 ± 2.10 | 57.56 ± 1.94 | 86.48 ± 0.87 | 82.41 ± 9.19 | 74.85 ± 2.58 |
|  | 1 | 85.13 ± 1.31 | 44.58 ± 3.28 | 48.23 ± 5.30 | 71.35 ± 8.06 | 71.80 ± 5.90 | 46.28 ± 5.13 |
|  | 2 | 79.23 ± 2.51 | 37.75 ± 4.19 | 46.52 ± 1.30 | 67.97 ± 4.19 | 62.69 ± 3.57 | 39.73 ± 1.05 |
|  | 4 | 70.19 ± 2.96 | 28.84 ± 5.56 | 34.33 ± 1.40 | 63.18 ± 1.08 | 50.86 ± 2.54 | 30.48 ± 6.90 |
|  | 6 | 61.91 ± 1.12 | 24.31 ± 6.22 | 27.83 ± 0.47 | 53.96 ± 5.48 | 46.23 ± 1.89 | 28.33 ± 5.77 |
|  | 12 | 42.20 ± 3.56 | 15.59 ± 4.36 | 16.32 ± 1.55 | 48.47 ± 1.58 | 34.78 ± 2.21 | 18.48 ± 2.58 |
|  | 24 | 22.75 ± 2.31 | 0.22 ± 0.11 | 6.03 ± 2.53 | 39.92 ± 6.25 | 23.74 ± 1.26 | 14.03 ± 6.07 |

|  |  |  |  |  |  |  |  |  |  |  |  |
| --- | --- | --- | --- | --- | --- | --- | --- | --- | --- | --- | --- |

**Table S6**: Stability of reference agent – DNS in ACN and 1:1 ACN:tetraborate buffer mixture

| Stability in: | **Time [hrs]** | **DNS** | Stability in: | **Time [hrs]** | **DNS** |
| --- | --- | --- | --- | --- | --- |
| ACN | 0 | 100.00 ± 0.00 | ACN:buffer | 0 | 100.00 ± 0.00 |
|  | 0.5 | 100.00 ± 0.00 |  | 0.5 | 98.47 ± 1.74 |
|  | 1 | 98.28 ± 2.43 |  | 1 | 96.86 ± 3.41 |
|  | 2 | 100.00 ± 0.00 |  | 2 | 77.93 ± 2.98 |
|  | 4 | 100.00 ± 0.00 |  | 4 | 43.80 ± 7.54 |
|  | 6 | 100.00 ± 0.00 |  | 6 | 26.09 ± 2.31 |
|  | 12 | 100.00 ± 0.00 |  | 12 | 20.58 ± 1.24 |
|  | 24 | 100.00 ± 0.00 |  | 24 | 14.96 ± 5.13 |

# Absorbance and fluorescence possibilities

**Table S7**: Comparison of absorbance and fluorescence intensities of the derivatization agents.

| **Derivatization agent** | **UV** | **FLD** |
| --- | --- | --- |
| 3-CP-NHS | 934.7 ± 8.5 | 7.3 ± 0.3 |
| 1-CiQ-NHS | 495.6 ± 4.8 | 1335.1 ± 15.6 |
| 3-CiQ-NHS | 1446.9 ± 32.5 | 1389.7 ± 20.4 |
| 5-CiQ-NHS | 1556.9 ± 28.5 | 3647.5 ± 35.5 |
| 6-CiQ-NHS | 1712.7 ± 34.2 | 15679.3 ± 55.6 |
| 2-CQ-NHS | 1544.3 ± 25.4 | 313.4 ± 4.8 |
| 3-CQ-NHS | 2166.7 ± 42.5 | 38.5 ± 1.2 |
| 4-CQ-NHS | 1026.1 ± 18.5 | 318.3 ± 15.0 |
| 5-CQ-NHS | 1196.3 ± 23.3 | 76.9 ± 1.2 |
| 6-CQ-NHS | 2141.0 ± 15.9 | 139.0 ± 0.8 |
| 7-CQ-NHS | 1861.0 ± 10.8 | 15.8 ± 0.3 |
| 8-CQ-NHS | 464.3 ± 5.5 | 46.6 ± 3.9 |

# MRM fragmentation and MS behavior of derivatized amino acids

**Table S8**: Summary of MRM fragmentation data of derivatized amino acids by various derivatization agents.

| **Derivatization agent** | **Amino acid** | **Precursor ion [M+H]^+^** | **Product ion Quantifier** | **Quantifier CE (eV)** | **Product ion Qualifier** | **Qualifier CE (eV)** |
| --- | --- | --- | --- | --- | --- | --- |
| 5-SiQ-SO_2_Cl | Ala | 281.0 | 129.0 | 40 | 194.0 | 24 |
|  | Cys | 313.1 | 129.1 | 40 | 226.0 | 16 |
|  | His | 347.0 | 301.0 | 20 | 129.0 | 56 |
|  | nVal | 309.8 | 129.4 | 40 | 129.4 | 24 |
|  | Ser | 297.0 | 129.0 | 40 | 251.0 | 20 |
|  | Tyr | 373.0 | 129.0 | 48 | 327.0 | 28 |
| 3-CP-COCl | Ala | 195.1 | 149.2 | 16 | 79.2 | 52 |
|  | Cys | 332.1 | 106.1 | 20 | 78.2 | 60 |
|  | His | 261.1 | 78.2 | 56 | 215.4 | 16 |
|  | nVal | 223.4 | 177.4 | 16 | 134.0 | 28 |
|  | Ser | 211.1 | 79.2 | 40 | 135.0 | 4 |
|  | Tyr | 287.1 | 79.2 | 52 | 78.2 | 36 |
| 5-CiQ-COCl | Ala | 245.1 | 129.0 | 48 | 199.1 | 24 |
|  | Cys | 432.1 | 129.1 | 68 | 190.0 | 32 |
|  | His | 311.1 | 128.0 | 52 | 155.9 | 28 |
|  | nVal | 274.4 | 228.1 | 24 | 129.9 | 52 |
|  | Ser | 261.1 | 129.0 | 44 | 215.0 | 24 |
|  | Tyr | 337.1 | 129.0 | 56 | 291.1 | 28 |
| 6-CiQ-COCl | Ala | 245.1 | 129.0 | 48 | 199.1 | 20 |
|  | Cys | 432.1 | 190.1 | 24 | 129.1 | 60 |
|  | His | 311.1 | 156.0 | 28 | 128.0 | 48 |
|  | nVal | 274.0 | 228.1 | 24 | 129.5 | 52 |
|  | Ser | 261.1 | 129.0 | 52 | 151.2 | 20 |
|  | Tyr | 337.1 | 129.0 | 56 | 293.2 | 24 |
| 6-CQ-COCl | Ala | 245.1 | 199.1 | 24 | 129.0 | 48 |
|  | Cys | 432.1 | 156.1 | 24 | 128.6 | 60 |
|  | His | 311.1 | 156.2 | 28 | 128.2 | 52 |
|  | nVal | 274.0 | 128.9 | 48 | 228.3 | 24 |
|  | Ser | 261.1 | 129.0 | 52 | 215.1 | 24 |
|  | Tyr | 337.1 | 129.0 | 52 | 293.2 | 24 |
| 3-CP-NHS | Ala | 195.1 | 149.2 | 16 | 79.2 | 52 |
|  | Cys | 332.1 | 106.1 | 20 | 78.2 | 60 |
|  | His | 261.1 | 215.4 | 16 | 78.2 | 56 |
|  | nVal | 223.4 | 177.4 | 16 | 134.0 | 28 |
|  | Ser | 211.1 | 79.2 | 40 | 135.0 | 4 |
|  | Tyr | 287.1 | 79.2 | 52 | 78.2 | 36 |
| 1-CiQ-NHS | Ala | 245.2 | 156.3 | 16 | 198.8 | 12 |
|  | Cys | 432.2 | 243.1 | 20 | 156.3 | 24 |
|  | His | 311.1 | 265.2 | 16 | 156.3 | 24 |
|  | nVal | 274.3 | 129.1 | 36 | 228.0 | 16 |
|  | Ser | 261.1 | 156.3 | 20 | 215.2 | 16 |
|  | Tyr | 337.2 | 291.1 | 16 | 156.3 | 24 |
| 3-CiQ-NHS | Ala | 245.2 | 198.8 | 12 | 156.3 | 20 |
|  | Arg | 330.2 | 156.2 | 32 | 128.3 | 52 |
|  | Asn | 288.3 | 271.,1 | 12 | 242.2 | 16 |
|  | Asp | 289.2 | 243.1 | 12 | 128.1 | 40 |
|  | Cys | 432.2 | 243.2 | 20 | 156.3 | 32 |
|  | Gln | 302.3 | 156.2 | 20 | 128.2 | 48 |
|  | Glu | 303.2 | 156.2 | 20 | 128.2 | 36 |
|  | Gly | 231.1 | 156.2 | 16 | 128.2 | 32 |
|  | His | 311.1 | 265.2 | 16 | 156.3 | 28 |
|  | ILe | 287.2 | 241.2 | 12 | 128.0 | 44 |
|  | Leu | 287.3 | 241.1 | 16 | 128.3 | 44 |
|  | Lys | 457.3 | 239.2 | 24 | 128.1 | 60 |
|  | Met | 305.2 | 210.9 | 20 | 128.2 | 40 |
|  | nVal | 274.0 | 228.0 | 12 | 129.2 | 36 |
|  | Phe | 321.2 | 275.3 | 16 | 128.1 | 40 |
|  | Pro | 271.3 | 156.3 | 20 | 128.1 | 40 |
|  | Ser | 261.1 | 215.2 | 12 | 156.3 | 20 |
|  | Thr | 275.1 | 257.2 | 12 | 128.1 | 36 |
|  | Trp | 360.5 | 296.2 | 24 | 128.2 | 52 |
|  | Tyr | 337.2 | 291.1 | 16 | 156.3 | 24 |
|  | Val | 273.2 | 227.2 | 12 | 128.1 | 36 |
| 5-CiQ-NHS | Ala | 245.1 | 129.0 | 48 | 199.1 | 24 |
|  | Cys | 432.1 | 129.1 | 68 | 190.0 | 32 |
|  | His | 311.1 | 128.0 | 52 | 155.9 | 28 |
|  | nVal | 274.4 | 129.9 | 52 | 228.1 | 24 |
|  | Ser | 261.1 | 129.0 | 44 | 215.0 | 24 |
|  | Tyr | 337.1 | 129.0 | 56 | 291.1 | 28 |
| 2-CQ-NHS | Ala | 245.2 | 156.3 | 20 | 199.2 | 16 |
|  | Cys | 432.3 | 243.1 | 24 | 156.3 | 28 |
|  | His | 311.1 | 265.2 | 16 | 156.3 | 28 |
|  | nVal | 274.2 | 129.1 | 36 | 228.1 | 16 |
|  | Ser | 161.1 | 156.3 | 20 | 215.2 | 12 |
|  | Tyr | 337.2 | 291.1 | 16 | 156.3 | 20 |
| 3-CQ-NHS | Ala | 245.2 | 199.1 | 20 | 129.1 | 44 |
|  | Arg | 330.2 | 156.3 | 28 | 128.2 | 56 |
|  | Asn | 288.3 | 156.2 | 20 | 128.2 | 44 |
|  | Asp | 289.2 | 199.1 | 24 | 128.9 | 48 |
|  | Cys | 432.3 | 190.2 | 32 | 156.3 | 24 |
|  | Gln | 302.6 | 285.1 | 4 | 156.3 | 28 |
|  | Glu | 303.2 | 156.2 | 28 | 129.1 | 56 |
|  | Gly | 231.1 | 185.0 | 24 | 128.9 | 48 |
|  | His | 311.1 | 265.2 | 16 | 156.3 | 28 |
|  | ILe | 287.2 | 241.2 | 20 | 158.0 | 32 |
|  | Leu | 287.2 | 241.2 | 24 | 129.3 | 52 |
|  | Lys | 457.1 | 156.2 | 36 | 128.2 | 60 |
|  | Met | 305.2 | 211.1 | 32 | 156.2 | 20 |
|  | nVal | 274.0 | 228.1 | 20 | 129.2 | 48 |
|  | Phe | 321.4 | 275.1 | 24 | 129.2 | 56 |
|  | Pro | 271.3 | 157.9 | 28 | 129.7 | 44 |
|  | Ser | 261.1 | 215.1 | 20 | 129.1 | 52 |
|  | Thr | 275.1 | 229.1 | 24 | 129.0 | 60 |
|  | Trp | 360.5 | 156.2 | 24 | 128.3 | 48 |
|  | Tyr | 337.2 | 291.1 | 24 | 129.1 | 60 |
|  | Val | 273.2 | 227.2 | 20 | 129.1 | 60 |
| 4-CQ-NHS | Ala | 245.2 | 171.2 | 24 | 199.1 | 20 |
|  | Cys | 432.2 | 129.2 | 56 | 156.3 | 20 |
|  | His | 311.1 | 156.3 | 28 | 265.2 | 16 |
|  | nVal | 274.2 | 200.1 | 28 | 228.1 | 20 |
|  | Ser | 261.1 | 128.8 | 56 | 215.1 | 24 |
|  | Tyr | 337.2 | 2129.1 | 52 | 291.1 | 20 |
| 5-CQ-NHS | Ala | 245.2 | 129.1 | 44 | 198.8 | 44 |
|  | Cys | 432.2 | 156.3 | 20 | 129.1 | 60 |
|  | His | 311.1 | 156.3 | 28 | 265.3 | 12 |
|  | nVal | 274.0 | 129.9 | 56 | 228.0 | 24 |
|  | Ser | 261.1 | 129.1 | 48 | 215.2 | 24 |
|  | Tyr | 337.2 | 129.1 | 60 | 156.3 | 28 |
| 6-CQ-NHS | Ala | 245.1 | 199.1 | 24 | 129.0 | 48 |
|  | Arg | 330.5 | 156.2 | 36 | 128.3 | 52 |
|  | Asn | 288.2 | 185.2 | 28 | 129.2 | 56 |
|  | Asp | 289.2 | 245.1 | 20 | 129.1 | 60 |
|  | Cys | 432.1 | 156.1 | 24 | 128.6 | 60 |
|  | Gln | 302.4 | 156.2 | 28 | 128.8 | 60 |
|  | Glu | 303.2 | 174.2 | 28 | 129.1 | 56 |
|  | Gly | 231.2 | 185.3 | 24 | 129.1 | 40 |
|  | His | 311.1 | 156.2 | 28 | 128.2 | 52 |
|  | ILe | 287.3 | 185.3 | 8 | 129.1 | 56 |
|  | Leu | 287.3 | 241.2 | 24 | 129.2 | 56 |
|  | Lys | 457.3 | 156.3 | 32 | 128.2 | 60 |
|  | Met | 305.5 | 211.2 | 28 | 129.1 | 48 |
|  | nVal | 274.0 | 228.3 | 24 | 128.9 | 48 |
|  | Phe | 321.4 | 156.2 | 8 | 129.5 | 52 |
|  | Pro | 271.3 | 227.5 | 20 | 128.2 | 44 |
|  | Ser | 261.1 | 215.1 | 24 | 129.0 | 52 |
|  | Thr | 275.4 | 231.2 | 16 | 129.1 | 52 |
|  | Trp | 360.5 | 314.2 | 28 | 129.2 | 56 |
|  | Tyr | 337.1 | 293.2 | 24 | 129.0 | 52 |
|  | Val | 273.2 | 227.1 | 24 | 129.1 | 48 |
| 7-CQ-NHS | Ala | 245.2 | 129.1 | 52 | 198.8 | 24 |
|  | Cys | 432.2 | 156.3 | 32 | 259.1 | 16 |
|  | His | 311.1 | 156.3 | 24 | 265.2 | 16 |
|  | nVal | 274.3 | 228.2 | 24 | 129.5 | 48 |
|  | Ser | 261.1 | 129.1 | 48 | 156.3 | 28 |
|  | Tyr | 337.2 | 129.1 | 52 | 156.3 | 24 |
| 8-CQ-NHS | Ala | 245.2 | 156.2 | 24 | 199.1 | 12 |
|  | Cys | 277.3 | 156.3 | 20 | 231.1 | 8 |
|  | His | 311.1 | 156.2 | 32 | 265.1 | 16 |
|  | nVal | 273.7 | 156.3 | 24 | 128.5 | 48 |
|  | Ser | 261.1 | 156.3 | 24 | 215.2 | 12 |
|  | Tyr | 337.2 | 156.3 | 28 | 291.2 | 16 |
| DNS | Ala | 323.2 | 157.1 | 28 | 170.2 | 16 |
|  | Arg | 408.5 | 170.2 | 36 | 129.0 | 52 |
|  | Asn | 366.4 | 252.2 | 12 | 170.2 | 24 |
|  | Asp | 367.1 | 157.1 | 32 | 170.2 | 20 |
|  | Gln | 380.5 | 170.2 | 32 | 234.2 | 12 |
|  | Glu | 381.2 | 170.2 | 24 | 157.1 | 36 |
|  | Gly | 309.2 | 157.1 | 32 | 170.2 | 20 |
|  | His | 389.2 | 170.2 | 36 | 110.0 | 28 |
|  | ILe | 365.3 | 157.1 | 36 | 170.1 | 20 |
|  | Leu | 365.1 | 157.1 | 32 | 234.0 | 20 |
|  | Lys | 613.3 | 170.2 | 48 | 317.4 | 28 |
|  | Met | 383.2 | 157.1 | 40 | 170.2 | 20 |
|  | nVal | 351.1 | 157.1 | 32 | 170.2 | 30 |
|  | Phe | 399.3 | 157.1 | 32 | 170.2 | 24 |
|  | Pro | 349.2 | 170.2 | 24 | 157.1 | 36 |
|  | Ser | 339.1 | 157.2 | 32 | 170.2 | 20 |
|  | Thr | 353.4 | 157.1 | 36 | 170.2 | 20 |
|  | Trp | 438.3 | 170.2 | 24 | 159.4 | 28 |
|  | Tyr | 648.2 | 170.2 | 48 | 369.0 | 28 |
|  | Val | 351.6 | 157.1 | 32 | 170.2 | 20 |
|  | Ala-[d_4_] | 327.2 | 158.1 | 28 | 170.2 | 20 |
|  | Arg-[d_7_] | 415.3 | 154.6 | 56 | 170.4 | 40 |
|  | Asn-[d_3_] | 368.5 | 170.4 | 8 | 233.5 | 12 |
|  | Asp-[d_3_] | 369.8 | 157.7 | 36 | 251.6 | 16 |
|  | Gln-[d_5_] | 384.9 | 170.3 | 28 | 234.1 | 16 |
|  | Glu-[d_5_] | 385.8 | 170.3 | 24 | 369.2 | 8 |
|  | Gly-[d_2_] | 310.9 | 157.7 | 32 | 170.3 | 20 |
|  | His-[d_5_] | 393.8 | 170.4 | 28 | 155.0 | 60 |
|  | Ile-[d_10_] | 374.9 | 157.8 | 32 | 170.0 | 24 |
|  | Leu-[d_10_] | 374.9 | 157.6 | 32 | 170.1 | 24 |
|  | Phe-[d_8_] | 407.2 | 170.1 | 24 | 157.9 | 32 |
|  | Pro-[d_7_] | 356.2 | 170.1 | 24 | 157.9 | 32 |
|  | Ser-[d_3_] | 342.5 | 158.3 | 32 | 170.4 | 20 |
|  | Thr-[d_5_] | 358.2 | 157.2 | 40 | 170.0 | 20 |
|  | Val-[d_8_] | 359.5 | 158.3 | 32 | 170.2 | 24 |

**Table S9**: MS responses of each derivatized amino acids with various derivatization agents

| **Agent** | **Normalized response [AUC_analyte_/AUC_caffeine_]** | | | | |
| --- | --- | --- | --- | --- | --- |
|  | **Ala** | **Cys** | **His** | **Ser** | **Tyr** |
| 5-SiQ-SO_2_Cl | 0.558 ± 0.02 | 0.001 ± 0.00 | 0.132 ± 0.01 | 0.163 ± 0.01 | 0.010 ± 0.00 |
| 3-CP-COCl | 0.186 ± 0.02 | 0.127 ± 0.03 | 0.347 ± 0.02 | 0.027 ± 0.00 | 0.048 ± 0.01 |
| 5-CiQ-COCl | 0.607 ± 0.12 | 0.168 ± 0.06 | 0.409 ± 0.06 | 0.774 ± 0.21 | 0.468 ± 0.02 |
| 6-CiQ-COCl | 0.506 ± 0.05 | 0.260 ± 0.04 | 0.528 ± 0.03 | 0.623 ± 0.07 | 0.429 ± 0.04 |
| 6-CQ-COCl | 0.583 ± 0.03 | 0.418 ± 0.08 | 1.249 ± 0.11 | 0.448 ± 0.03 | 0.023 ± 0.02 |
| 3-CP-NHS | 0.598 ± 0.06 | 0.295 ± 0.04 | 0.580 ± 0.03 | 0.048 ± 0.01 | 0.055 ± 0.01 |
| 1-CiQ-NHS | 0.231 ± 0.01 | 0.131 ± 0.02 | 0.925 ± 0.09 | 0.057 ± 0.01 | 0.059 ± 0.00 |
| 3-CiQ-NHS | 1.262 ± 0.10 | 0.404 ± 0.08 | 1.110 ± 0.14 | 0.308 ± 0.05 | 0.560 ± 0.09 |
| 5-CiQ-NHS | 1.483 ± 0.06 | 0.483 ± 0.10 | 0.367 ± 0.06 | 0.437 ± 0.08 | 0.338 ± 0.04 |
| 6-CiQ-NHS | 0.969 ± 0.05 | 0.385 ± 0.06 | 0.827 ± 0.11 | 0.522 ± 0.06 | 0.868 ± 0.10 |
| 2-CQ-NHS | 0.410 ± 0.03 | 0.179 ± 0.05 | 1.062 ± 0.08 | 0.073 ± 0.01 | 0.069 ± 0.01 |
| 3-CQ-NHS | 0.660 ± 0.00 | 0.769 ± 0.07 | 0.817 ± 0.10 | 0.171 ± 0.04 | 0.209 ± 0.05 |
| 4-CQ-NHS | 0.276 ± 0.02 | 0.141 ± 0.01 | 0.274 ± 0.00 | 0.100 ± 0.01 | 0.093 ± 0.01 |
| 5-CQ-NHS | 1.456 ± 0.01 | 0.448 ± 0.04 | 0.701 ± 0.06 | 0.931 ± 0.01 | 0.369 ± 0.07 |
| 6-CQ-NHS | 0.549 ± 0.04 | 0.263 ± 0.03 | 0.916 ± 0.21 | 0.142 ± 0.00 | 0.357 ± 0.07 |
| 7-CQ-NHS | 1.538 ± 0.02 | 0.683 ± 0.08 | 0.710 ± 0.07 | 0.293 ± 0.03 | 0.449 ± 0.06 |
| 8-CQ-NHS | 1.169 ± 0.15 | 0.040 ± 0.00 | 2.106 ± 0.07 | 0.284 ± 0.05 | 0.771 ± 0.10 |
| DNS | 0.862 ± 0.10 | 0.007 ± 0.00 | 0.476 ± 0.08 | 0.470 ± 0.06 | 1.133 ± 0.07 |

# Derivatization kinetic profiles

**Table S10**: Kinetic profiles of serine derivatization with prepared derivatization agents.

| **Derivatization agent** | **Reaction time [min]** | | | | | |
| --- | --- | --- | --- | --- | --- | --- |
|  | **0** | **1** | **5** | **15** | **30** | **60** |
| 5-SiQ-SO_2_Cl | 0.000 ± 0.00 | 0.026 ± 0.01 | 0.107 ± 0.01 | 0.140 ± 0.01 | 0.121 ± 0.001 | 0.119 ± 0.00 |
| 3-CP-COCl | 0.000 ± 0.00 | 0.060 ± 0.00 | 0.064 ± 0.00 | 0.065 ± 0.00 | 0.056 ± 0.00 | 0.048 ± 0.00 |
| 5-CiQ-COCl | 0.000 ± 0.00 | 0.358 ± 0.02 | 0.356 ± 0.01 | 0.383 ± 0.02 | 0.367 ± 0.03 | 0.288 ± 0.01 |
| 6-CiQ-COCl | 0.000 ± 0.00 | 0.576 ± 0.01 | 0.641 ± 0.01 | 0.631 ± 0.03 | 0.642 ± 0.03 | 0.652 ± 0.03 |
| 6-CQ-COCl | 0.000 ± 0.00 | 0.388 ± 0.02 | 0.382 ± 0.02 | 0.394 ± 0.02 | 0.397 ± 0.01 | 0.363 ± 0.01 |
| 3-CP-NHS | 0.000 ± 0.00 | 0.010 ± 0.00 | 0.010 ± 0.00 | 0.020 ± 0.00 | 0.030 ± 0.00 | 0.030 ± 0.00 |
| 1-CiQ-NHS | 0.000 ± 0.00 | 0.150 ± 0.05 | 0.381 ± 0.01 | 0.502 ± 0.05 | 0.503 ± 0.05 | 0.483 ± 0.05 |
| 3-CiQ-NHS | 0.000 ± 0.00 | 0.150 ± 0.01 | 0.440 ± 0.04 | 0.611 ± 0.05 | 0.578 ± 0.04 | 0.600 ± 0.04 |
| 5-CiQ-NHS | 0.000 ± 0.00 | 0.003 ± 0.00 | 0.017 ± 0.00 | 0.046 ± 0.01 | 0.073 ± 0.01 | 0.137 ± 0.02 |
| 6-CiQ-NHS | 0.000 ± 0.00 | 0.157 ± 0.09 | 0.416 ± 0.07 | 0.961 ± 0.02 | 1.150 ± 0.02 | 1.280 ± 0.04 |
| 2-CQ-NHS | 0.000 ± 0.00 | 0.740 ± 0.02 | 0.790 ± 0.01 | 0.760 ± 0.01 | 0.760 ± 0.01 | 0.750 ± 0.01 |
| 3-CQ-NHS | 0.000 ± 0.00 | 0.177 ± 0.02 | 0.544 ± 0.05 | 0.820 ± 0.07 | 0.927 ± 0.08 | 0.959 ± 0.08 |
| 4-CQ-NHS | 0.000 ± 0.00 | 0.122 ± 0.06 | 0.374 ± 0.02 | 0.635 ± 0.03 | 0.813 ± 0.04 | 0.921 ± 0.05 |
| 5-CQ-NHS | 0.000 ± 0.00 | 0.110 ± 0.08 | 0.530 ± 0.04 | 1.348 ± 0.09 | 1.877 ± 0.05 | 2.144 ± 0.03 |
| 6-CQ-NHS | 0.000 ± 0.00 | 0.009 ± 0.01 | 0.047 ± 0.01 | 0.095 ± 0.01 | 0.142 ± 0.02 | 0.182 ± 0.02 |
| 7-CQ-NHS | 0.000 ± 0.00 | 0.090 ± 0.06 | 0.320 ± 0.02 | 0.620 ± 0.04 | 0.800 ± 0.04 | 0.910 ± 0.04 |
| 8-CQ-NHS | 0.000 ± 0.00 | 0.117 ± 0.04 | 0.551 ± 0.01 | 1.467 ± 0.05 | 2.258 ± 0.07 | 2.527 ± 0.09 |

# Chromatographic retention behavior of derivatives

**Table S11**: Comparison of retention times [min] of derivatized amino acids using different agents.

| **Derivatized product** | **Derivatization agent** | **Retention time of derivatized amino acids [min]** | | | | |
| --- | --- | --- | --- | --- | --- | --- |
|  |  | **Ala** | **Ser** | **His** | **Cys** | **Tyr** |
| Sulfonamide | 3-SP-SO_2_Cl | - | - | - | - | - |
|  | 5-SiQ-SO_2_Cl | 2.5 | 2.2 | 2.0 | 2.3 | 2.6 |
| Amide | 3-CP-NHS | 1.2 | 0.6 | 0.5 | 2.5 | 2.3 |
|  | 1-CiQ-NHS | 3.1 | 2.7 | 2.4 | 3.9 | 3.3 |
|  | 3-CiQ-NHS | 3.2 | 2.8 | 2.5 | 3.9 | 3.4 |
|  | 5-CiQ-NHS | 1.4 | 0.6 | 0.5 | 2.7 | 2.3 |
|  | 6-CiQ-NHS | 2.1 | 0.9 | 0.7 | 2.9 | 2.5 |
|  | 2-CQ-NHS | 3.3 | 2.9 | 2.6 | 4.1 | 3.4 |
|  | 3-CQ-NHS | 2.7 | 2.4 | 2.2 | 3.5 | 3.0 |
|  | 4-CQ-NHS | 2.4 | 1.8 | 1.3 | 3.3 | 2.7 |
|  | 5-CQ-NHS | 2.0 | 0.9 | 0.7 | 3.1 | 2.5 |
|  | 6-CQ-NHS | 2.3 | 1.6 | 1.2 | 3.2 | 2.7 |
|  | 7-CQ-NHS | 2.5 | 2.0 | 1.9 | 3.3 | 2.8 |
|  | 8-CQ-NHS | 3.1 | 2.5 | 2.4 | 3.2 | 3.2 |

# pH influence on derivatization

**Table S12**: Effect of pH on the derivatization efficiency of the 6-CiQ-NHS agent.

| **pH** | **Ala** | **Cys** | **His** | **Ser** | **Tyr** |
| --- | --- | --- | --- | --- | --- |
| 3.0 | 10.9 ± 1.0 | 9.2 ± 0.5 | 6.8 ± 1.8 | 6.8 ± 0.9 | 4.5 ± 1.0 |
| 5.0 | 21.0 ± 3.8 | 67.4 ± 10.5 | 13.6 ± 2.5 | 9.2 ± 1.6 | 6.5 ± 2.4 |
| 6.0 | 80.7 ± 2.2 | 118.9 ± 20.5 | 156.1 ± 56.9 | 26.1 ± 5.4 | 84.4 ± 23.5 |
| 6.5 | 232.1 ± 18.5 | 1107.6 ± 106.8 | 220.2 ± 50.8 | 130.6 ± 20.5 | 313.9 ± 86.2 |
| 7.0 | 658.5 ± 22.8 | 9491.5 ± 1078.5 | 531.5 ± 84.9 | 660.1 ± 156.4 | 1153.6 ± 348.7 |
| 7.5 | 2204.9 ± 387.5 | 52916.8 ± 4987.2 | 1621.8 ± 327.9 | 1736.4 ± 519.5 | 4410.7 ± 1057.6 |
| 8.0 | 4655.2 ± 589.2 | 65927.6 ± 15783.2 | 3127.4 ± 847.8 | 3762.8 ± 847.5 | 8252.1 ± 864.3 |
| 8.5 | 74546.6 ± 20580.1 | 813347.6 ± 30475.2 | 21240.6 ± 4781.6 | 94165.5 ± 11794.2 | 202456.1 ± 21498.2 |
| 9.0 | 376266.0 ± 31789.3 | 1005884.5 ± 105647.3 | 85117.4 ± 5784.3 | 358349.2 ± 28149.1 | 744208.5 ± 36479.2 |
| 9.5 | 232372.2 ± 22576.0 | 876164.1 ± 65647.6 | 57176.6 ± 8876.2 | 255455.1 ± 18479.2 | 544568.5 ± 40257.5 |
| 10.0 | 160990.1 ± 15879.4 | 680416.6 ± 82746.2 | 14395.7 ± 5798.2 | 61767.8 ± 8497.3 | 91042.3 ± 14983.3 |

# Effects of the temperature on the derivatization reaction

**Table S13**: Data for effect of temperature on derivatization reaction, example on 6-CiQ-amino acid derivatives.

| **Temperature (°C)** | **Ala** | **Cys** | **His** | **Ser** | **Tyr** |
| --- | --- | --- | --- | --- | --- |
| 25 °C | 122174 ± 7630 | 131226 ± 27324 | 18033 ± 1385 | 57723 ± 3864 | 89238 ± 3383 |
| 40 °C | 86267 ± 3965 | 105000 ± 9467 | 10702 ± 313 | 34556 ± 1633 | 50813 ± 2857 |
| 60 °C | 185917 ± 294 | 73220 ± 9269 | 17365 ± 1385 | 54065 ± 3369 | 81670 ± 6581 |


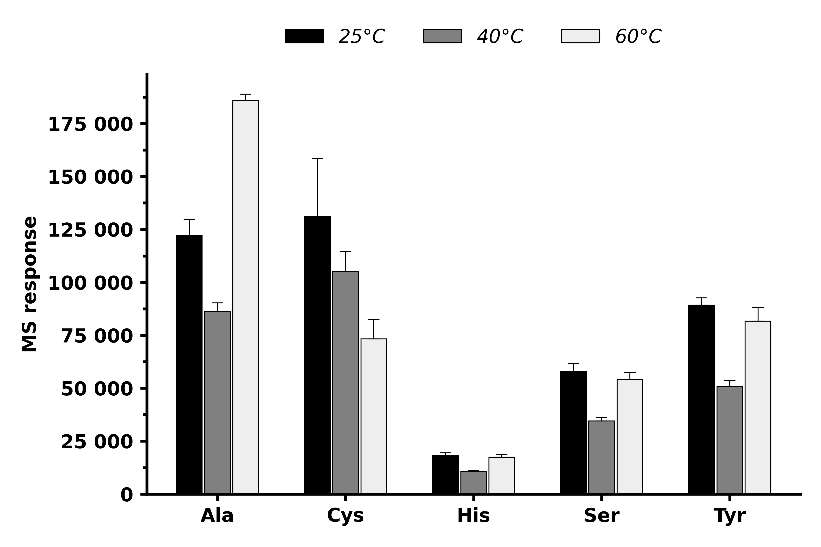


**Figure S20**: Effect of temperature on efficacy of the derivatization, example on 6-CiQ-amino acid derivatives.

# Chromatographic separation


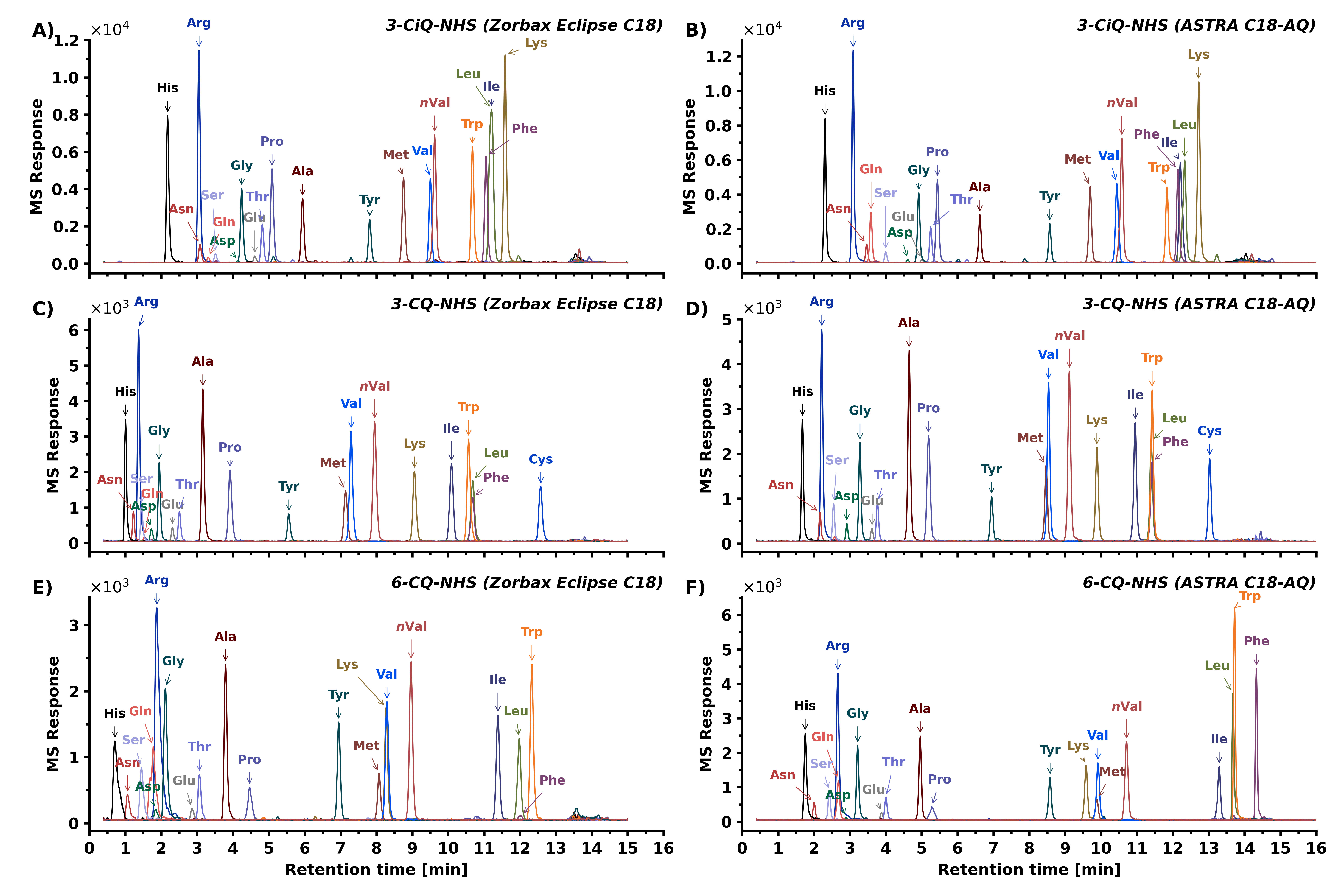


**Figure S21**: Chromatographic separation of 3-CiQ-NHS amino acid derivatives on Zorbax Eclipse C18 (A) and ASTRA C18-AQ (B) columns, 3-CQ-NHS amino acid derivatives on Zorbax Eclipse C18 (C) and ASTRA C18-AQ (D), and 6-CQ-NHS amino acid derivatives on Zorbax Eclipse C18 (E) and ASTRA C18-AQ (F) columns.

# Stability of derivatized products

**Table S14**: Stability of 6-CiQ-derivatized amino acid standards in the autosampler.

|  | **Ala** | | **Arg** | | **Asn** | | **Asp** | | **Cys** | |
| --- | --- | --- | --- | --- | --- | --- | --- | --- | --- | --- |
| **Time [hrs]** | **AVG** | **SD** | **AVG** | **SD** | **AVG** | **SD** | **AVG** | **SD** | **AVG** | **SD** |
| **0** | 100 | 0.00 | 100 | 0.00 | 100 | 0.00 | 100 | 0.00 | 100 | 0.00 |
| **4** | 88 | 1.63 | 98 | 2.93 | 91 | 2.80 | 89 | 4.96 | 100 | 0.69 |
| **8** | 88 | 2.51 | 89 | 3.23 | 87 | 0.33 | 88 | 5.88 | 100 | 0.00 |
| **12** | 87 | 2.93 | 86 | 0.86 | 81 | 1.33 | 85 | 2.02 | 98 | 3.30 |
| **24** | 86 | 1.99 | 87 | 0.88 | 84 | 2.63 | 82 | 3.36 | 95 | 2.20 |
|  |  |  |  |  |  |  |  |  |  |  |
|  | **Gln** | | **Glu** | | **Gly** | | **His** | | **Ile** | |
| **Time [hrs]** | **AVG** | **SD** | **AVG** | **SD** | **AVG** | **SD** | **AVG** | **SD** | **AVG** | **SD** |
| **0** | 100 | 0.00 | 100 | 0.00 | 100 | 0.00 | 100 | 0.00 | 100 | 0.00 |
| **4** | 96 | 1.50 | 91 | 5.75 | 95 | 2.81 | 92 | 1.84 | 98 | 2.46 |
| **8** | 91 | 2.66 | 83 | 4.80 | 90 | 1.37 | 87 | 1.72 | 96 | 3.51 |
| **12** | 83 | 5.60 | 78 | 4.17 | 88 | 2.26 | 85 | 2.85 | 99 | 1.26 |
| **24** | 86 | 2.64 | 76 | 4.39 | 90 | 0.10 | 82 | 2.09 | 97 | 2.48 |
|  |  |  |  |  |  |  |  |  |  |  |
|  | **Leu** | | **Lys** | | **Met** | | **Phe** | | **Pro** | |
| **Time [hrs]** | **AVG** | **SD** | **AVG** | **SD** | **AVG** | **SD** | **AVG** | **SD** | **AVG** | **SD** |
| **0** | 100 | 0.00 | 100 | 0.00 | 100 | 0.00 | 100 | 0.00 | 100 | 0.00 |
| **4** | 97 | 2.71 | 97 | 4.01 | 100 | 0.00 | 97 | 3.18 | 86 | 3.99 |
| **8** | 94 | 1.61 | 94 | 0.68 | 92 | 3.03 | 95 | 4.40 | 84 | 2.86 |
| **12** | 95 | 0.26 | 93 | 2.88 | 91 | 5.86 | 96 | 2.90 | 81 | 3.10 |
| **24** | 95 | 1.96 | 89 | 2.74 | 99 | 1.57 | 97 | 3.34 | 79 | 1.76 |
|  |  |  |  |  |  |  |  |  |  |  |
|  | **Ser** | | **Thr** | | **Trp** | | **Tyr** | | **Val** | |
| **Time [hrs]** | **AVG** | **SD** | **AVG** | **SD** | **AVG** | **SD** | **AVG** | **SD** | **AVG** | **SD** |
| **0** | 100 | 0.00 | 100 | 0.00 | 100 | 0.00 | 100 | 0.00 | 100 | 0.00 |
| **4** | 93 | 3.73 | 92 | 1.78 | 98 | 2.45 | 96 | 0.96 | 98 | 0.87 |
| **8** | 86 | 4.28 | 89 | 0.53 | 97 | 3.37 | 93 | 3.07 | 90 | 2.88 |
| **12** | 83 | 3.71 | 87 | 0.04 | 94 | 4.98 | 89 | 5.22 | 91 | 1.26 |
| **24** | 84 | 3.50 | 87 | 2.03 | 99 | 1.59 | 90 | 2.38 | 93 | 2.76 |

**Table S15**: Stability of 6-CiQ-derivatized deuterated amino acid standards in the autosampler.

|  | **Ala-[d_4_]** | | **Arg-[d_7_]** | | **Asn-[d_3_]** | | **Asp-[d_3_]** | | **Cys-[d_2_]** | |
| --- | --- | --- | --- | --- | --- | --- | --- | --- | --- | --- |
| **Time [hrs]** | **AVG** | **SD** | **AVG** | **SD** | **AVG** | **SD** | **AVG** | **SD** | **AVG** | **SD** |
| **0** | 100 | 0.00 | 100 | 0.00 | 100 | 0.00 | 100 | 0.00 | 100 | 0.00 |
| **4** | 100 | 0.34 | 95 | 2.78 | 87 | 0.85 | 93 | 2.25 | 91 | 3.08 |
| **8** | 93 | 1.43 | 90 | 2.60 | 88 | 0.13 | 92 | 2.56 | 91 | 0.32 |
| **12** | 90 | 1.32 | 89 | 1.67 | 86 | 4.78 | 87 | 5.07 | 87 | 0.95 |
| **24** | 86 | 1.55 | 84 | 0.85 | 83 | 1.07 | 81 | 3.75 | 86 | 4.18 |
|  |  |  |  |  |  |  |  |  |  |  |
|  | **Gln-[d_5_]** | | **Glu-[d_5_]** | | **Gly-[d_2_]** | | **His-[d_5_]** | | **Ile-[d_10_]** | |
| **Time [hrs]** | **AVG** | **SD** | **AVG** | **SD** | **AVG** | **SD** | **AVG** | **SD** | **AVG** | **SD** |
| **0** | 100 | 0.00 | 100 | 0.00 | 100 | 0.00 | 100 | 0.00 | 100 | 0.00 |
| **4** | 95 | 0.47 | 93 | 1.83 | 100 | 0.00 | 95 | 3.35 | 97 | 2.39 |
| **8** | 90 | 1.15 | 88 | 3.36 | 91 | 3.19 | 90 | 1.52 | 97 | 3.42 |
| **12** | 88 | 0.53 | 85 | 2.38 | 87 | 1.46 | 88 | 4.17 | 96 | 2.08 |
| **24** | 83 | 1.26 | 77 | 1.18 | 79 | 0.31 | 86 | 1.19 | 92 | 2.29 |
|  |  |  |  |  |  |  |  |  |  |  |
|  | **Leu-[d_10_]** | | **Lys-[d_8_]** | | **Met-[d_8_]** | | **Phe-[d_8_]** | | **Pro-[d_7_]** | |
| **Time [hrs]** | **AVG** | **SD** | **AVG** | **SD** | **AVG** | **SD** | **AVG** | **SD** | **AVG** | **SD** |
| **0** | 100 | 0.00 | 100 | 0.00 | 100 | 0.00 | 100 | 0.00 | 100 | 0.00 |
| **4** | 99 | 0.89 | 100 | 0.00 | 97 | 1.45 | 93 | 3.24 | 91 | 1.18 |
| **8** | 98 | 1.33 | 100 | 0.00 | 94 | 1.69 | 94 | 4.21 | 91 | 2.40 |
| **12** | 98 | 1.83 | 87 | 3.31 | 95 | 0.76 | 91 | 0.36 | 88 | 0.57 |
| **24** | 96 | 1.97 | 82 | 5.72 | 92 | 2.46 | 89 | 3.09 | 85 | 3.42 |
|  |  |  |  |  |  |  |  |  |  |  |
|  | **Ser-[d_3_]** | | **Thr-[d_5_]** | | **Trp-[d_8_]** | | **Tyr-[d_7_]** | | **Val-[d_8_]** | |
| **Time [hrs]** | **AVG** | **SD** | **AVG** | **SD** | **AVG** | **SD** | **AVG** | **SD** | **AVG** | **SD** |
| **0** | 100 | 0.00 | 100 | 0.00 | - | - | 100 | 0.00 | 100 | 0.00 |
| **4** | 97 | 3.73 | 98 | 2.53 | - | - | 97 | 3.21 | 99 | 0.70 |
| **8** | 90 | 4.28 | 90 | 1.37 | - | - | 97 | 3.55 | 98 | 195 |
| **12** | 88 | 3.71 | 90 | 0.50 | - | - | 94 | 2.68 | 98 | 2.93 |
| **24** | 87 | 3.50 | 87 | 3.05 | - | - | 90 | 3.50 | 95 | 1.41 |

**Table S16**: Stability of 6-CiQ-derivatized amino acid standards in – 80°C freezer.

|  | **Ala** | | **Arg** | | **Asn** | | **Asp** | | **Cys** | |
| --- | --- | --- | --- | --- | --- | --- | --- | --- | --- | --- |
| **Time [hrs]** | **AVG** | **SD** | **AVG** | **SD** | **AVG** | **SD** | **AVG** | **SD** | **AVG** | **SD** |
| **0** | 100 | 0.00 | 100 | 0.00 | 100 | 0.00 | 100 | 0.00 | 100 | 0.00 |
| **24** | 89 | 2.01 | 83 | 2.40 | 85 | 1.81 | 87 | 0.13 | 100 | 0.00 |
|  |  |  |  |  |  |  |  |  |  |  |
|  | **Gln** | | **Glu** | | **Gly** | | **His** | | **Ile** | |
| **Time [hrs]** | **AVG** | **SD** | **AVG** | **SD** | **AVG** | **SD** | **AVG** | **SD** | **AVG** | **SD** |
| **0** | 100 | 0.00 | 100 | 0.00 | 100 | 0.00 | 100 | 0.00 | 100 | 0.00 |
| **24** | 90 | 0.87 | 81 | 3.59 | 87 | 2.48 | 82 | 3.36 | 100 | 0.34 |
|  |  |  |  |  |  |  |  |  |  |  |
|  | **Leu** | | **Lys** | | **Met** | | **Phe** | | **Pro** | |
| **Time [hrs]** | **AVG** | **SD** | **AVG** | **SD** | **AVG** | **SD** | **AVG** | **SD** | **AVG** | **SD** |
| **0** | 100 | 0.00 | 100 | 0.00 | 100 | 0.00 | 100 | 0.00 | 100 | 0.00 |
| **24** | 98 | 1.80 | 90 | 0.13 | 100 | 0.00 | 96 | 1.99 | 75 | 1.58 |
|  |  |  |  |  |  |  |  |  |  |  |
|  | **Ser** | | **Thr** | | **Trp** | | **Tyr** | | **Val** | |
| **Time [hrs]** | **AVG** | **SD** | **AVG** | **SD** | **AVG** | **SD** | **AVG** | **SD** | **AVG** | **SD** |
| **0** | 100 | 0.00 | 100 | 0.00 | 100 | 0.00 | 100 | 0.00 | 100 | 0.00 |
| **24** | 81 | 3.76 | 88 | 1.75 | 100 | 0.00 | 91 | 0.64 | 94 | 2.11 |

**Table S17**: Stability of 6-CiQ-derivatized deuterated amino acid standards in – 80°C freezer.

|  | **Ala-[d_4_]** | | **Arg-[d_7_]** | | **Asn-[d_3_]** | | **Asp-[d_3_]** | | **Cys-[d_2_]** | |
| --- | --- | --- | --- | --- | --- | --- | --- | --- | --- | --- |
| **Time [hrs]** | **AVG** | **SD** | **AVG** | **SD** | **AVG** | **SD** | **AVG** | **SD** | **AVG** | **SD** |
| **0** | 100 | 0.00 | 100 | 0.00 | 100 | 0.00 | 100 | 0.00 | 100 | 0.00 |
| **24** | 90 | 2.07 | 88 | 2.76 | 79 | 2.72 | 79 | 2.42 | 89 | 0.67 |
|  |  |  |  |  |  |  |  |  |  |  |
|  | **Gln-[d_5_]** | | **Glu-[d_5_]** | | **Gly-[d_2_]** | | **His-[d_5_]** | | **Ile-[d_10_]** | |
| **Time [hrs]** | **AVG** | **SD** | **AVG** | **SD** | **AVG** | **SD** | **AVG** | **SD** | **AVG** | **SD** |
| **0** | 100 | 0.00 | 100 | 0.00 | 100 | 0.00 | 100 | 0.00 | 100 | 0.00 |
| **24** | 86 | 1.97 | 83 | 2.59 | 80 | 2.24 | 91 | 2.88 | 95 | 2.18 |
|  |  |  |  |  |  |  |  |  |  |  |
|  | **Leu-[d_10_]** | | **Lys-[d_8_]** | | **Met-[d_8_]** | | **Phe-[d_8_]** | | **Pro-[d_7_]** | |
| **Time [hrs]** | **AVG** | **SD** | **AVG** | **SD** | **AVG** | **SD** | **AVG** | **SD** | **AVG** | **SD** |
| **0** | 100 | 0.00 | 100 | 0.00 | 100 | 0.00 | 100 | 0.00 | 100 | 0.00 |
| **24** | 98 | 0.79 | 78 | 3.12 | 94 | 3.03 | 91 | 0.81 | 85 | 2.59 |
|  |  |  |  |  |  |  |  |  |  |  |
|  | **Ser-[d_3_]** | | **Thr-[d_5_]** | | **Trp-[d_8_]** | | **Tyr-[d_7_]** | | **Val-[d_8_]** | |
| **Time [hrs]** | **AVG** | **SD** | **AVG** | **SD** | **AVG** | **SD** | **AVG** | **SD** | **AVG** | **SD** |
| **0** | 100 | 0.00 | 100 | 0.00 | - | - | 100 | 0.00 | 100 | 0.00 |
| **24** | 92 | 0.83 | 90 | 1.44 | - | - | 94 | 2.62 | 99 | 0.89 |

**Table S18**: Stability of 6-CiQ-derivatized amino acid standards after evaporation and reconstitution.

|  | **Ala** | | **Arg** | | **Asn** | | **Asp** | | **Cys** | |
| --- | --- | --- | --- | --- | --- | --- | --- | --- | --- | --- |
| **Time [hrs]** | **AVG** | **SD** | **AVG** | **SD** | **AVG** | **SD** | **AVG** | **SD** | **AVG** | **SD** |
| **0** | 100 | 0.00 | 100 | 0.00 | 100 | 0.00 | 100 | 0.00 | 100 | 0.00 |
| **24** | 100 | 0.00 | 100 | 0.00 | 100 | 0.00 | 98 | 3.22 | 100 | 0.00 |
|  |  |  |  |  |  |  |  |  |  |  |
|  | **Gln** | | **Glu** | | **Gly** | | **His** | | **Ile** | |
| **Time [hrs]** | **AVG** | **SD** | **AVG** | **SD** | **AVG** | **SD** | **AVG** | **SD** | **AVG** | **SD** |
| **0** | 100 | 0.00 | 100 | 0.00 | 100 | 0.00 | 100 | 0.00 | 100 | 0.00 |
| **24** | 99 | 0.29 | 82 | 3.35 | 100 | 0.00 | 99 | 2.01 | 100 | 0.00 |
|  |  |  |  |  |  |  |  |  |  |  |
|  | **Leu** | | **Lys** | | **Met** | | **Phe** | | **Pro** | |
| **Time [hrs]** | **AVG** | **SD** | **AVG** | **SD** | **AVG** | **SD** | **AVG** | **SD** | **AVG** | **SD** |
| **0** | 100 | 0.00 | 100 | 0.00 | 100 | 0.00 | 100 | 0.00 | 100 | 0.00 |
| **24** | 99 | 2.44 | 100 | 0.14 | 87 | 2.53 | 100 | 0.52 | 82 | 1.39 |
|  |  |  |  |  |  |  |  |  |  |  |
|  | **Ser** | | **Thr** | | **Trp** | | **Tyr** | | **Val** | |
| **Time [hrs]** | **AVG** | **SD** | **AVG** | **SD** | **AVG** | **SD** | **AVG** | **SD** | **AVG** | **SD** |
| **0** | 100 | 0.00 | 100 | 0.00 | 100 | 0.00 | 100 | 0.00 | 100 | 0.00 |
| **24** | 100 | 0.00 | 100 | 0.00 | 100 | 0.00 | 98 | 3.44 | 99 | 0.76 |

**Table S19**: Stability of 6-CiQ-derivatized deuterated amino acid standards after evaporation and reconstitution.

|  | **Ala-[d_4_]** | | **Arg-[d_7_]** | | **Asn-[d_3_]** | | **Asp-[d_3_]** | | **Cys-[d_2_]** | |
| --- | --- | --- | --- | --- | --- | --- | --- | --- | --- | --- |
| **Time [hrs]** | **AVG** | **SD** | **AVG** | **SD** | **AVG** | **SD** | **AVG** | **SD** | **AVG** | **SD** |
| **0** | 100 | 0.00 | 100 | 0.00 | 100 | 0.00 | 100 | 0.00 | 100 | 0.00 |
| **24** | 96 | 2.71 | 98 | 0.70 | 80 | 3.80 | 90 | 2.75 | 85 | 2.77 |
|  |  |  |  |  |  |  |  |  |  |  |
|  | **Gln-[d_5_]** | | **Glu-[d_5_]** | | **Gly-[d_2_]** | | **His-[d_5_]** | | **Ile-[d_10_]** | |
| **Time [hrs]** | **AVG** | **SD** | **AVG** | **SD** | **AVG** | **SD** | **AVG** | **SD** | **AVG** | **SD** |
| **0** | 100 | 0.00 | 100 | 0.00 | 100 | 0.00 | 100 | 0.00 | 100 | 0.00 |
| **24** | 88 | 2.42 | 85 | 2.18 | 93 | 1.03 | 99 | 1.73 | 89 | 1.86 |
|  |  |  |  |  |  |  |  |  |  |  |
|  | **Leu-[d_10_]** | | **Lys-[d_8_]** | | **Met-[d_8_]** | | **Phe-[d_8_]** | | **Pro-[d_7_]** | |
| **Time [hrs]** | **AVG** | **SD** | **AVG** | **SD** | **AVG** | **SD** | **AVG** | **SD** | **AVG** | **SD** |
| **0** | 100 | 0.00 | 100 | 0.00 | 100 | 0.00 | 100 | 0.00 | 100 | 0.00 |
| **24** | 98 | 1.93 | 90 | 4.07 | 80 | 2.06 | 80 | 0.09 | 81 | 3.82 |
|  |  |  |  |  |  |  |  |  |  |  |
|  | **Ser-[d_3_]** | | **Thr-[d_5_]** | | **Trp-[d_8_]** | | **Tyr-[d_7_]** | | **Val-[d_8_]** | |
| **Time [hrs]** | **AVG** | **SD** | **AVG** | **SD** | **AVG** | **SD** | **AVG** | **SD** | **AVG** | **SD** |
| **0** | 100 | 0.00 | 100 | 0.00 | - | - | 100 | 0.00 | 100 | 0.00 |
| **24** | 86 | 2.80 | 90 | 1.07 | - | - | 89 | 0.32 | 100 | 0.60 |

# Linearity in presence and absence of matrix for 6-CiQ-NHS derivatization

**
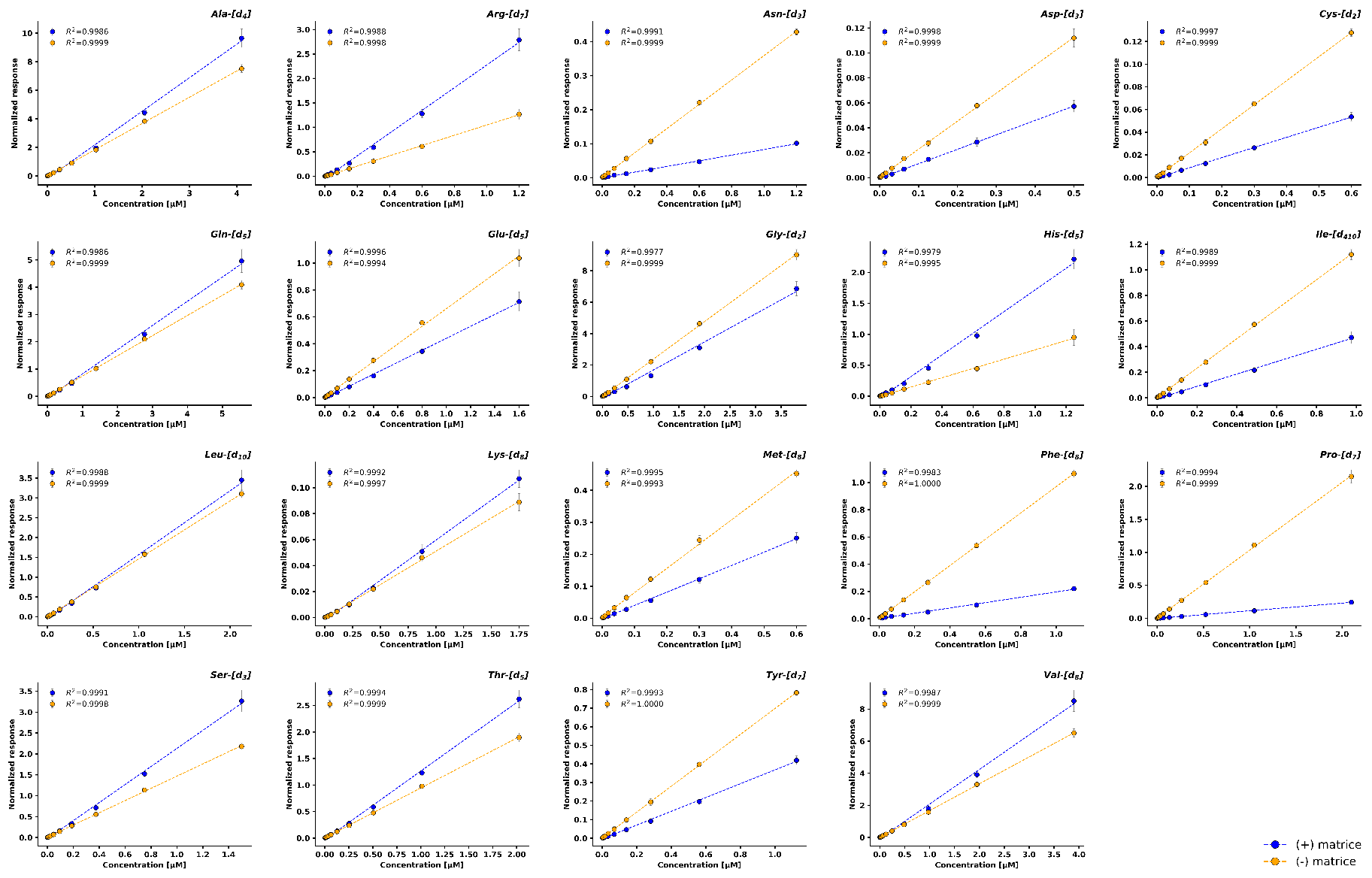
**

**Figure S22**: Comparison of calibration curves with and without matrix for 6-CiQ-NHS derivatized deuterated amino acids highlighting response differences.

# Calibration curves for amino acids quantification





**Figure S23**: Calibration curves for quantification of amino acids in the sample
